# The Role of Willpower in Major Depressive Disorder: An fMRI Study

**DOI:** 10.1002/brb3.70921

**Published:** 2025-10-20

**Authors:** Burak Yulug, Burak Uygur, Dila Sayman, Seyda Cankaya, Behcet Ayyıldız, Sevilay Ayyıldız, Ece Ozdemir Oktem, Aynur Akturk, Ahmet Ozsimsek, Ali Behram Salar, Mehmet Ozansoy, Lutfu Hanoglu, Ozlem Altay, Halil Aziz Velioglu, Adil Mardinoglu

**Affiliations:** ^1^ Department of Neurology and Clinical Neuroscience Alanya Alaaddin Keykubat University Alanya Turkey; ^2^ Department of Psychiatry Alanya Alaaddin Keykubat University Alanya Turkey; ^3^ Anatomy PhD Program, Graduate School of Health Sciences Kocaeli University Kocaeli Turkey; ^4^ Department of Neuroradiology, School of Medicine Technical University of Munich Munich Germany; ^5^ TUM‐NIC Neuroimaging Center, School of Medicine Technical University of Munich Munich Germany; ^6^ Functional Imaging and Cognitive‐Affective Neuroscience Lab (fINCAN), Health Sciences and Technology Research Institute (SABITA) Istanbul Medipol University Istanbul Turkey; ^7^ Department of Physiology Bahcesehir University Istanbul Turkey; ^8^ Department of Neurology Istanbul Medipol University Istanbul Turkey; ^9^ KTH Royal Institute of Science and Technology Stockholm Sweden; ^10^ Center for Psychiatric Neuroscience Feinstein Institutes for Medical Research Manhasset New York USA

**Keywords:** depression, dorsal DMN, fMRI, frontal pole, putamen, self‐directness

## Abstract

**Introduction:**

The brain network correlates of personality traits in major depressive disorder (MDD) have not yet been investigated. Furthermore, it is still unclear whether personality traits relate to the depressive episode.

**Methods:**

This study assessed network properties, depression severity, and personality traits in patients with MDD (*n* = 25) compared with age‐ and sex‐matched healthy controls (*n* = 22). We performed TCI questionnaire which assesses novelty seeking (NS, an urge to explore new experiences with heightened emotional responses), harm avoidance (HA, the tendency to hold back when faced with unpleasant situations), reward dependence (RD, a tendency to seek and value rewards rooted in social recognition), persistence (P, an individual's ability to remain focused and driven toward goals despite encountering challenges), self‐directness (SD, an expression of willpower that enables individuals to adapt their behavior to situational demands while remaining focused on their personal goals and values), cooperativeness (C, a behavioral trait reflecting a person's general approach to others; ranging from friendly and cooperative to hostile), and self‐transcendence (ST, lessening of self‐centeredness, allowing for expanded empathy) traits of participants.

**Results:**

MDD patients with distinctive character traits exhibited significant differences in terms of depression diagnosis and severity of Hamilton Depression Rating Scale scores compared to the controls. The MDD patients also exhibited reduced resting‐state network activity between the posterior default mode network, right putamen, and right frontal pole, while SD was significantly less frequently diagnosed in MDD patients. In evaluating the network correlates, differences in the SD traits were significantly associated with critical brain network alterations that were not evident in other traits.

**Discussion:**

To the best of our knowledge, this is the first study to provide preliminary evidence of an abnormal connectome in the SD trait in MDD, thus providing convincing evidence for personalized antidepressant treatment strategies in MDD. A small sample size and our depression group being not drug‐naive were our limitation for this research.

## Graphical abstract

Individuals with self‐directness (SD) traits exhibited significantly decreased resting‐state functional connectivity of the dorsal DMN with the right frontal pole and putamen. Also, SD was the only personality trait that was significantly correlated with depression severity and putaminal network activity.

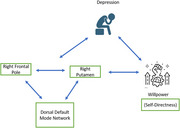

## 1 Introduction

For more than a century, psychiatrists have disagreed over the connection between personality features and major depressive disorder (MDD) (Zaninotto et al. 2015). This hypothesis was initially controversial but has gained support in recent years following the development of the psychobiological model of personality by Cloninger (1994) which classified an individual's fundamental temperamental and personality traits into various categories. However, although temperament relates to biologically based and stable individual characteristics, character has been observed to be alterable due to its link to socialization, making it difficult to make a clear distinction between the two constructs in real‐life situations. A substantial body of research supports these findings by indicating that personality traits all possess multivariate properties and interact with one another over the course of a healthy life (Gusnard et al. 2003; Matsudaira and Kitamura 2006). In addition, several studies have reported that patients with MDD exhibit higher levels of temperament and character features, such as harm avoidance (HA), and lower levels of self‐directedness (SD) (an expression of willpower that enables individuals to adapt their behavior to situational demands while remaining focused on their personal goals and values, goal‐directed behavior) than healthy controls (Cloninger 1994; Garcia et al. 2017).

Despite these promising findings, however, it is still unclear exactly how the temperament and character dimensions are associated with depressive disorder. This is because temperament and character differ in terms of the underlying types of neurobiological mechanisms and the way they are modulated by the human brain (Celikel et al. 2009; Cloninger 2004; Zaninotto et al. 2015). For instance, temperament which can be defined in psychology as genetic predispositions that shape a person's emotional reactions and behavioral tendencies has been reported to be controlled by various neurotransmitter systems of the brain such as serotonin and oxytocin, and density of the neuroreceptors, both of which contribute to shaping emotional and behavioral responses on an individual level (Andari et al. 2014; Farde et al. 2018). Temperament also exhibits a considerable correlation with neurobiologically based behavioral patterns compared to character traits (Andari et al. 2014; Cloninger 2004; A. Josefsson et al. 2007). Moreover, temperament characteristics are highly linked to structural changes, such as volume, thickness, blood flow alterations, in the frontal, temporal, cerebellar, limbic, thalamic, and basal ganglia areas (Andari et al. 2014; Farde et al. 2018; Johnson et al. 1999; Laricchiuta et al. 2014). There is also a substantial overlap between brain regions involved in temperament characteristics and those involved in character dimensions, although they appear to function separately (Kaasinen et al. 2005; Turner et al. 2003). Further clarify, each individual's character dimensions are shaped by temperament traits that are genetically diverse and influenced by individual structural variations.

Despite providing useful morphometric data, these previous studies were far from specific to a particular brain region and failed to provide a dynamic model for describing the underlying neural correlates of personality. Another handicap is that these studies were primarily conducted among healthy individuals and are, therefore, not relevant to the pathogenesis of depressive disorder.

Recent advances in brain imaging and analysis techniques (such as functional magnetic resonance imaging [fMRI] and graph analysis) have made it possible to identify the dynamic interaction between brain regions and to acquire new dynamic insights into the mechanisms of depression and emotional regulation. Particularly noteworthy in this context is the role of resting‐state dynamics in depressive disorder and personality, both of which rely on the maintenance of brain circuits in a “ready state” as opposed to “continuous” mental activity. Within this framework, only a handful of studies have investigated the connections between the brain and personality on the network level in healthy individuals, revealing significant dynamic changes in the brain beyond what had been determined for the same regions by conventional structural neuroimaging studies.

One exciting study by Li et al. revealed that activity in the brain network during salience detection and attention regulation is essential for certain individual temperament features (Li et al. 2017). Additional neuroimaging studies confirmed that HA and novelty seeking (NS) are connected not only to a particular localized activity in the brain (thalamus, prefrontal cortex, and the limbic and cerebellar areas) but also to the functional connectivity of brain regions in depression (insula, cingulate gyrus, and dorsolateral prefrontal cortex) (Markett et al. 2013).

The role of specific network activity has also been confirmed in healthy individuals. For example, SD has been linked to a specific serotonergic activity in the posterior cingulate region (post‐default mode network [DMN]), medial frontal gyrus, and superior temporal gyrus in healthy individuals (Tuominen et al. 2013). A recent study by Takeuchi et al. also observed that SD was significantly positively correlated with verbal creativity and divergent thinking (Takeuchi et al. 2015), which acts as a buffer against depression (Zuo et al. 2021), and was associated with critical activity in the right putamen. The SD trait protects against depression, possibly due to its effect on the emotional self‐regulation system, and its protective effect is therefore lower in MDD (Legault and Inzlicht 2013; Muñoz‐Navarro et al. 2022). Given that poor SD is characterized by a reduced capacity to control impulses and regulate one's ego power, resulting in insufficient self‐control, lack of direction, and absence of self‐acceptance (Evren and Bozkurt 2016). It is not surprising that the SD trait may have a significant impact on the development of depression. Research conducted by Tse and others has demonstrated that people with low SD are more liable to depression. It is also mentioned in the same study that people with low SD would be less of an expert in obtaining social rewards that could build up their self‐esteem and capability to decrease stress, which is in line with the social reinforcement theory of depression (Tse et al. 2011). Richter and Eisemann (2002) suggested that personality traits in depressive patients may alter early in childhood in some patients and can be a predictor of a beginning of depression. This raises an intriguing question whether the opposite pattern may also hold true for preventing the development of depression. Within that context, there have been a few studies that suggest that SD may offer protection against depression according to the reinforcement theory of depression, a process that plays a crucial role in the response to antidepressant treatment (Tse et al. 2011). For instance, several studies indicated a significant relationship between willpower, depression, and anxiety (Alqahtani et al. 2018; Kahn et al. 2013; Manongi et al. 2020; Talaei‐Khoei et al. 2017; Wang et al. 2017). In a reverse pattern, in explaining the effect of cognitive, emotional regulation strategies on affection and anxiety, it can be concluded that since cognition, affection, and behavior are in close interaction with each other (Tabei et al. 2019). The cognitive emotion regulation can change the function of cognitive systems (including memory, attention, and consciousness) by controlling the attention and cognitive outcomes of emotion (Karatzias et al. 2018) and then regulate the emotion which in turn might result with decreased depressive symptoms and physical and social survival of the person (Curtiss et al. 2017). From a neurobiological point of view, this is consistent with earlier work of Mayberg suggesting that cortico‐limbic axis regulation might be responsible for the development of depression wherein strategies focusing on decreasing limbic metabolism and/or increasing the cortical functions might result in an antidepressant response (Mayberg et al. 2000).

Despite these encouraging results in healthy individuals, to the best of our knowledge, there has been no detailed investigation of the brain network correlates of personality characteristics in MDD patients and healthy controls.

In order to fill this gap, the primary aim of the current study was to detect any difference in temperament and character dimensions between depressive patients and healthy controls and to establish whether any association exists between personality traits and the severity of depression. The secondary aim was to examine the brain network correlates of the personality traits in depressive patients, especially those who presented with different severity levels, and a control group. To the best of our knowledge, this is the first study to directly compare network properties, personality traits, and initial depression severity in MDD patients and healthy controls.

## 2 Methods

### 2.1 Participants

For this cross‐sectional study, 25 depressive outpatients (age between 21 and 55 years) and 22 healthy controls (age between 18 and 59 years) patients were recruited by their clinicians from the Alanya Alaaddin Keykubat University Hospital, Turkey. No participants were excluded from the study. Participants were native Turkish speakers (Supporting Information). Turkish TCI (Temperament and Character Inventory) questionnaire which requires participants to answer right or wrong to 240 questions and assesses participants’ personality traits (NS, HA, persistence [P], SD, self‐transcendence, cooperativeness [C], reward dependence) was completed by all participants. The Turkish TCI questionnaire was read and answered by solely participants without any interruptions by any observer. There was no significant difference in groups in terms of age and gender (Table 1). Five males and twenty females were in the depression group, and nine males and thirteen females were in the control group. All patients met the Diagnostic and Statistical Manual of Mental Disorders, Fifth Edition (DSM‐V) for MDD. Diagnoses were confirmed by experienced psychiatrists using the Hamilton Depression Rating Scale (HDRS). This was applied to determine the mean depression scores for each temperament and character group. Exclusion criteria for all patients were: (a) a score of less than 23 on the Mini‐Mental State Examination (MMSE) (Folstein et al. 1975), (b) a history of head trauma or stroke, or current or previous substance abuse/dependence, (c) clinical evidence of other major psychiatric or neurological disorders, and (d) use of prescribed anti‐dementia medications. The study protocol was approved by the Ethics Committee of Istanbul Medipol University (report no. 10840098‐604.01.01‐E.19402), and all patients provided written informed consent after a complete examination of the study.

**TABLE 1:** General descriptive statistics between depression and control groups.

Variables	Depression (*n* = 25)	Control (*n* = 21)	*p* value
Education year (median [IQR])	11.48 (6)	14.476 (4)	0.021^a^
Age (mean ± SD)	34.16 ± 10.411	34.091 ± 11.191	0.983
HDRS (median [IQR])	9.2 (5)	3.136 (6)	< 0.001^a^
Novelty seeking (NS) (mean ± SD)	18.8 ± 5.57	15.27 ± 4.06	0.018^a^
Harm avoidance (HA) (mean ± SD)	23.88 ± 6.97	19.41 ± 7.14	0.035^a^
Reward dependence (RD) (mean ± SD)	12.16 ± 4.5	12.55 ± 3.61	0.750
Persistence (P) (mean ± SD)	4.36 ± 1.93	5.77 ± 1.63	0.010^a^
Self‐directness (SD) (mean ± SD)	19.56 ± 6.53	27.5 ± 6.89	<.001^a^
Cooperativeness (C) (median [IQR])	26 (11)	28.5 (6.25)	0.034^a^
Self‐transcendence (ST) (median [IQR])	16.680 (13)	19.636 (12.3)	0.104
Gender, female *n* (%)	20 (80%)	13 (60%)	0.118

Abbreviations: C, cooperativeness; HA, harm avoidance; HDRS, Hamilton Depression Rating Scale; NS, novelty seeking; P, persistence; RD, reward dependence; SD, self‐directness; ST, self‐transcendence.

^a^ We observed significant differences in the education year (*p* = 0.021), HDRS (*p* < 0.001), and several character traits. NS (*p* = 0.018), HA (*p* = 0.035), P (*p* = 0.010), SD (*p* < 0.001), and C (*p* = 0.034) showed significant differences between groups. Based on normal distribution, parametric and nonparametric tests were used. Normally distributed data investigated with Student's *t*‐test and presented as mean‐SD; non‐normally distributed data investigated with Mann–Whitney *U* test and presented as median (IQR). The comparison of categorical data between the two or more groups was done using the chi‐square test.

### 2.2 fMRI Recording and Analysis

Resting‐state fMRI was performed using a Signa Explorer device (General Electric Company, USA). Patients were instructed to remain motionless and to keep their eyes open throughout the recording. This lasted approximately 12.5 min and involved 255 volumes (TR 3000 ms, TE 30 ms, FA 90°), FOV of 256 × 256 × 156 mm (FH × AP × RL), a voxel size of 4 × 4 × 4 mm, a flip angle of 90°, and 39 slices. The anatomical T1 image of the sagittal section was recorded using 156 slices, a TR/TE of 1/3.7, FOV of 256 × 256 × 156 mm (FH × AP × RL), and voxel sizes of 1 × 1 × 1 mm. In our fMRI analysis, we harnessed the capabilities of the FMRIB Software Library (FSL) tools, specifically tailored for the Linux Mint 18.3 Sylvia operating system (ver.6.0.0). Initial DICOM single slice images were seamlessly converted to NIFTI format using the dcm2niix command‐line tool for subsequent processing. Employing the FSL “fsl_anat,” we conducted brain extraction, while preprocessing steps with the FSL FEAT tool were collectively applied across all subjects. These steps included alignment of functional images to the center image, motion correction, and the application of a high‐pass filter with a time constant of 150 s well below the 0.01–0.1 Hz range characterizing resting‐state networks. A smoothing process with a 5‐mm FWHM kernel followed suit. Independent Component Analysis (ICA) unfolded with the FSL “melodic” tool. To purify functional data from artifacts stemming from movement, respiratory and cardiovascular sources, and device‐dependent slow signal fluctuations, ICA components underwent meticulous manual labeling based on their time course, frequency content, and spatial distribution. The FSL “fsl_regfilt” tool was then employed to expunge labeled artifact components from the functional data. Post‐preprocessing, individual anatomical images were registered to the MNI152 standard brain using the FSL “applywarp” tool. Group‐level ICA ran on standard brain images, yielding ICA components. The FSL “dual_regression” tool facilitated the calculation of individual time series and spatial maps of ICA components, deploying a two‐stage regression approach. Data distribution underwent scrutiny via the permutation method (5000 times), utilizing a two‐sample unpaired *t*‐test design matrix for inter‐group comparisons. Corrected *p* values, factoring in multiple comparisons, adhered to a significance threshold of *p* < 0.05.

In addition to ICA‐based approaches, a seed‐based correlation analysis was conducted to explore connectivity patterns of specific functional networks. For this purpose, we employed resting‐state ROIs from the Stanford Functional Imaging in Neuropsychiatric Disorders Lab (FIND Lab) (Shirer et al. 2012). These ROIs included the following 14 functionally defined seed regions: anterior salience, auditory, basal ganglia, dorsal DMN, high visual, language, left executive control network (LECN), posterior salience, precuneus, primary visual, right executive control network (RECN), sensorimotor, ventral DMN, and visuospatial. These seeds were selected due to their established roles in large‐scale brain networks commonly implicated in neuropsychiatric disorders. By using these predefined, anatomically constrained masks, the seed‐based approach allowed us to evaluate network‐specific connectivity variations in a hypothesis‐driven manner.

### 2.3 Statistics

Jamovi (version 2.3.19.0) was used for statistical analyses. Descriptive values were reported as mean ± standard deviation. The Shapiro–Wilk test was used to check the normality of the variables. The Mann–Whitney *U* test was used to compare the depressive and control groups in terms of HDRS scores, age, and years spent in education. Spearman correlation analysis was performed to identify the association between the extraction values (putamen and frontal pole) and clinical scores in the depression and control groups.

## 3 Results

### 3.1 Descriptive Statistics

Demographic and clinical characteristics of the patients in the two groups (MDD and control) are presented in Tables 1. There were no statistically significant differences between the groups in terms of any demographic or clinical characteristics (*p* > 0.05, Shapiro–Wilk test). Results from comparisons of HamD scores between the depression (d) and control (c) groups are presented in Table 1 (mean ± standard deviation). Statistically significant differences in HamD scores were observed between the depression and control groups (Table 1) in terms of total NS (depression, 18.8 ± 5.57; control, 15.27 ± 4.06), HA (depression, 23.88 ± 6.97; control, 19.41 ± 7.14), P (depression, 4.36 ± 1.93; control, 5.77 ± 1.63), SD (depression, 19.56 ± 6.53; control, 27.75 ± 6.89), and C (depression, 23.84 ± 7.08; control, 27.82 ± 5.97) scores (*p* < 0.05, Mann–Whitney *U* test). While 20 female patients were in the MDD group, 13 female participants were in the control (*p* = 0.118).

**TABLE 2:** Clusters of fMRI activity.

Cluster region	Voxels	1‐p‐MAX	1‐p‐MAX X (vox)	1‐p‐MAX Y (vox)	1‐p‐MAX Z (vox)	1‐p‐COG X (vox)	1‐p‐COG Y (vox)	1‐p‐COG Z (vox)	COPE‐MAX	COPE‐MAX X (vox)	COPE‐MAX Y (vox)	COPE‐MAX Z (vox)	COPE‐MEAN
Right frontal pole	33	0.964	29	88	38	29	87.5	38.5	4.91	29	88	38	4.17
Right putamen	57	0.971	33	64	31	33.4	64.3	31.2	5.51	33	64	31	4.08

Categorical TCI assessment: SD personality traits were diagnosed significantly more in the controls than in the MDD patients (*p* < 0.05 Fisher's exact test; likelihood ratio: 7.16, *p* = 0.007) compared to other traits (*p* > 0.05), Table 3.

### 3.2 fMRI Results

**FIGURE 1:**
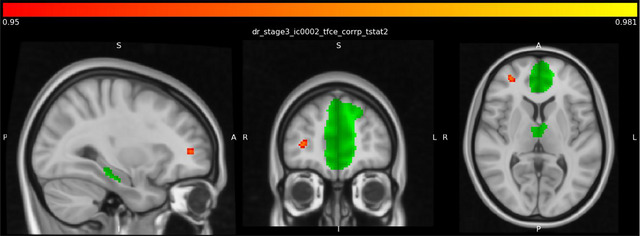
Higher right frontal pole connectivity with dorsal DMN in the control group than in the depression group. The green color represents the dorsal DMN regions as ROI. Meanwhile, the red color indicates higher connections with the dorsal DMN in control groups compared to the depression group.

**FIGURE 2:**
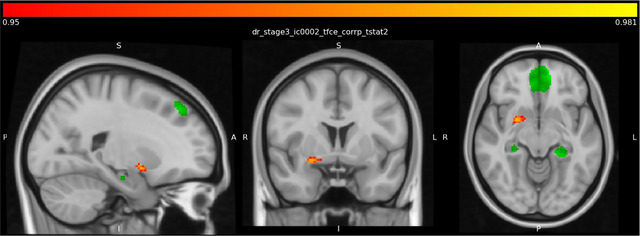
Higher right hemisphere putamen connectivity with dorsal DMN in the control group than in the depression group. The green color represents the dorsal DMN regions as ROI. Meanwhile, the red color indicates higher connections with the dorsal DMN in control groups compared to the depression group.

Analysis revealed significantly increased resting‐state functional connectivity of the dorsal DMN with the right frontal pole (Figure 1, Table 2) and putamen (Figure 2, Table 2) in the control group compared to the depression group.

Based on the significant areas described above, we extracted putamen and frontal pole voxel region data separately for each individual and applied additional correlation analysis to evaluate the relationship between clinical scores and specific connectivity patterns.

### 3.3 Correlations Between Temperament Scores, Resting‐State Activity, and HDRS Scores

SD was the only temperament trait significantly and negatively correlated with HDRS scores (*p* < 0.001, rho = −0.628, Table 4). Interestingly, HDRS scores were also negatively correlated with putaminal activity (*p* = 0.002, rho = −0.447, Table 4), while the SD trait was positively correlated with putaminal activity (*p* = 0.002, rho = 0.435). There were significant differences between HDRS scores and HA (*r* = 0.369^*^, *p* = 0.015), SD (*r* = −0.534^***^, *p* < 0.001), C (*r* = −0.0481^**^, *p* = 0.001) scores after adjusting for age, education year, and gender (Table 5). Significant differences also persisted between HDRS scores and putamen (rho = −0.424^**^, *p* = 0.005) and frontal pole (rho = −0.390, *p* = 0.010) connectivity between SD scores and putamen (rho = 0.409^*^, *p* = 0.006) after adjusting for age, education year, and gender (Table 5).

**TABLE 3:** The differentiation of TCI in the groups.

TCI	MDD (*N* = 25)	Controls (*N* = 22)	*p* ^a^
NS	4	2	0.066
HA	4	2	0.066
RD	3	2	0.186
P	3	4	0.156
SD	2	6	0.026
ST	5	4	0.239
C	4	2	0.199

^a^ The comparison of categorical data between the two or more groups was done using the chi‐square test.

**TABLE 4:** Correlation analysis between the extracted fMRI values and clinical scores.

Correlation matrix		HDRS	NS	HA	RD	P	SD	C	ST	putamen	frontal pole
HDRS	Spearman's rho	—									
	*p* value	—									
NS	Spearman's rho	0.239	—								
	*p* value	0.106	—								
HA	Spearman's rho	0.439	−0.023	—							
	*p* value	0.002^**^	0.88	—							
RD	Spearman's rho	−0.104	0.028	0.065	—						
	*p* value	0.486	0.852	0.665	—						
P	Spearman's rho	−0.396	−0.321	−0.199	0.232	—					
	*p* value	0.006^**^	0.028^*^	0.179	0.116	—					
SD	Spearman's rho	−0.628	−0.375	−0.595	0.016	0.526	—				
	*p* value	< 0.001^***^	0.009^**^	< 0.001^***^	0.917	< 0.001^***^	—				
C	Spearman's rho	−0.39	−0.295	−0.407	0.432	0.428	0.426	—			
	*p* value	0.007^**^	0.044	0.005^**^	0.002^**^	0.003^**^	0.003^**^	—			
ST	Spearman's rho	−0.244	0.061	−0.056	0.309	0.314	0.064	0.281	—		
	*p* value	0.099	0.685	0.709	0.035^*^	0.032^*^	0.669	0.056	—		
Putamen	Spearman's rho	−0.447	−0.392	−0.194	−0.127	0.277	0.435	0.173	0.026	—	
	*p* value	0.002^**^	0.007^**^	0.192	0.394	0.06	0.002^**^	0.244	0.86	—	
Frontal pole	Spearman's rho	−0.342	−0.218	−0.196	−0.042	0.242	0.236	0.219	−0.029	0.549	—
	*p* value	0.019^*^	0.141	0.187	0.781	0.102	0.11	0.139	0.849	< 0.001^***^	—

^*^ *p* < 0.05, ^**^ *p* < 0.01, and ^***^ *p* < 0.001.

**TABLE 5:** Partial correlation analysis (adjusted for age, education year, and gender) between the extracted fMRI values and clinical scores.

	HDRS	NS	HA	RD	P	SD	C	ST	Putamen	Frontal pole
HDRS	—	0.293 *p* = 0.057	0.369^*^ *p* = 0.015	−0.119 *p* = 0.448	−0.390^**^ *p* = 0.010	−0.534^***^ *p* < 0.001	−0.481^**^ *p* = 0.001	−0.206 *p* = 0.185	−0.424^**^ *p* = 0.005	−0.390^*^ *p* = 0.010
NS		—	−0.128 *p* = 0.414	0.116 *p* = 0.458	−0.298 *p* = 0.052	−0.437^**^ *p* = 0.003	−0.157 *p* = 0.314	0.044 *p* = 0.782	−0.366^*^ *p* = 0.016	−0.283 *p* = 0.066
HA			—	0.161 *p* = 0.303	−0.059 *p* = 0.709	−0.428^**^ *p* = 0.004	−0.264 *p* = 0.087	−0.087 *p* = 0.893	−0.160 *p* = 0.306	−0.130 *p* = 0.404
RD				—	0.310^*^ *p* = 0.043	−0.022 *p* = 0.888	0.534^***^ *p* < 0.001	0.416^**^ *p* = 0.006	−0.058 *p* = 0.710	0.109 *p* = 0.488
P					—	0.443^**^ *p* = 0.003	0.445^**^ *p* = 0.003	0.313^*^ *p* = 0.041	0.238 *p* = 0.125	0.293 *p* = 0.057
SD						—	0.421^**^ *p* = 0.005	−0.100 *p* = 0.523	0.409^**^ *p* = 0.006	0.192 *p* = 0.218
C							—	0.369^*^ *p* = 0.015	0.161 *p* = 0.302	0.329^*^ *p* = 0.031
ST								—	−0.021 *p* = 0.894	−0.037 *p* = 0.814
Putamen									—	0.685^***^ *p* < 0.001
Frontal pole										—

*Note*: Controlling for education year, age, and gender.

^*^ *p* < 0.05, ^**^ *p* < 0.01, and ^***^ *p* < 0.001.

## 4 Discussion

This is the first study to compare the network correlates of temperament and character traits in MDD patients and healthy controls and to show that some personality traits may be associated with different functional network characteristics in MDD. The patients with MDD had considerably different personality and temperament features to those of the control volunteers, with HA, P, C, and SD being the primary personality and character traits that differed substantially in the MDD group. This confirmed previous reports that certain personality traits are more common in MDD patients (Richardson et al. 2020; Sacchet et al. 2017). Interestingly, categorical assessment showed the control individuals were significantly more frequently diagnosed with the SD personality trait than the MDD patients. Individuals with SD traits also exhibited significantly lower depression scores compared to individuals without SD traits. Our correlation analysis confirmed this interesting finding by showing that SD was the only personality trait that was significantly correlated with depression severity and putaminal network activity. To be more specific, in evaluating the correlation among personality traits (that were found to be significantly different between depression and the control group [NS, P, HA, C, SD]), none of the personality traits except of SD were significantly associated both with HDRS scores and putaminal activity. Our detailed statistical analysis showed that NS was not significantly correlated with HDRS scores. Furthermore, P, HA, and C were not correlated with putaminal activity (see Table 4) which led us to conclude that SD was the only parameter that was significantly associated with depression severity and putaminal functional activity.

This finding was both surprising and unique in demonstrating that some specific brain network alterations are correlated with impaired SD personality features. This was defined as “willpower” by Cloninger in the early 1990s, described as “a purposeful life perspective with self‐awareness and responsibility” (Cloninger 1994).

From the clinical perspective, our findings are incompatible with previous research suggesting that only HA plays a dominant role in MDD (Chien and Dunner 1996; Cloninger et al. 1993; Cloninger 2004; K. Josefsson et al. 2011). This may be connected to the multi‐finality process, in which each character and temperament trait interact early in the causal chain, ultimately shaping the development and prognosis of depression. Alternatively, this dynamic interplay of personality characteristics might possibly be complicated by the interaction of certain key neurotransmitters, such as serotonin for HD, although to the best of our knowledge, no particular one has been explicitly confirmed.

Although our network findings seem to accord with some pivotal brain network studies involving healthy individuals and patients with depression, these have either not sufficiently evaluated the network correlates of personality trait features or have not assessed the network features of personality traits only in both conditions.

Limited depression studies have identified DMN activity and its altered functional connectivity as characteristics of MDD (Hamilton et al. 2015). For instance, Korgaonkar et al. attributed a special role to the degree of intra‐ and inter‐network connectivity (involving the DMN and VAN) in the depression treatment response (Korgaonkar et al. 2020), while Chou et al. showed greater DMN activation in individuals who have greater risk at developing depression (Chou et al. 2023). Another novel study by Saccaro et al. showed significantly lower *N*‐acetyl aspartate levels in frontal lobe in depressive patients compared to healthy controls which is in line with the possible involvement of DMN in depression (Saccaro et al. 2024). However, those authors did not sufficiently evaluate the link between depression severity, brain network correlates, and personality and temperament traits.

Nevertheless, our results together with those from Cloninger, Korgaonkar et al., and others support the idea that some specific network features may determine the response of the network to stressful life events, which may in turn have significant effects on the development and severity of depression (Cloninger 1994; Cloninger et al. 1993; Korgaonkar et al. 2020).

It should be noted that our findings also suggest a role of DMN in healthy individuals, one related to emotional positive states, dopaminergic activity, and the extroversion trait (Brooks et al. 2020; Simon et al. 2020; Toschi et al. 2018; Tuominen et al. 2013). In that context, a specific role can be attributed to the putamen with its significant dopamine source which helps it to function as a dynamic part of this network that may be disrupted in depressive patients.

A good example of this appeared in a recent study by Sacchet et al., who revealed a significantly decreased putaminal volume in depressive patients (Sacchet et al. 2017), a finding which fits well with our observation of decreased dorsal DMN‐putaminal activity in MDD patients. These findings also accord well with our own findings and with recent research providing valuable evidence for the association of DMN and putamen activity with the SD trait (Gardini et al. 2009).

Considering all these findings, our research findings seem to be unique in verifying the network correlates of SD in patients with MDD and healthy controls. They also appear to extend our current understanding of the personalized antidepressant treatment perspective by linking personality to specially altered network activity in patients with MDD, thus yielding one of the missing pieces of the puzzle.

### 4.1 Limitations

There are several limitations to the present study, not least the relatively small sample size. Second, our depression group was not drug‐naive, which may, over the long term, have influenced brain connectivity changes. However, our SD‐non‐SD participants exhibited no difference in terms of depression severity, or treatment type and response, and no association was determined between the use of antidepressant medication and character scores. This is also suggested by previous data showing that neither depressive episodes nor antidepressant treatment may produce long‐term changes in SD or other traits (Agosti and McGrath 2002; Hellerstein et al. 2000; Mundt et al. 2002).

## 5 Conclusion

To conclude, we have revealed that decreased right putamen connectivity with dorsal DMN was correlated with SD personality trait and clinical depression scores in the MDD group. This suggest that the SD trait might be an important characteristic trait that might interact with the development and severity of depression. Despite its limitations, this is one of the few studies to provide compelling evidence of an anomalous connectome in SD traits in MDD. Despite this positive data, however, the role of personality traits both on depression neurobiology and underlying specific network correlates is an unexplored issue while our results might provide a novel dimension to therapeutic approaches.

## Author Contributions

**Burak Yulug**: conceptualization, writing–original draft, methodology, resources, visualization. **Burak Uygur**: conceptualization, visualization. **Dila Sayman**: data curation, investigation, writing–original draft. **Seyda Cankaya**: data curation, methodology, supervision. **Behcet Ayyıldız**: software, formal analysis. **Sevilay Ayyıldız**: software, formal analysis. **Ece Ozdemir Oktem**: investigation, supervision. **Aynur Akturk**: data curation, investigation. **Ahmet Ozsimsek**: validation. **Ali Behram Salar**: formal analysis, software. **Mehmet Ozansoy**: writing–review and editing, validation. **Lutfu Hanoglu**: writing–review and editing, supervision. **Ozlem Altay**: writing–original draft. **Halil Aziz Velioglu**: formal analysis, software, writing–original draft. **Adil Mardinoglu**: funding acquisition, writing–review and editing, supervision.

## Conflicts of Interest

The authors declare no conflicts of interest.

## Peer Review

The peer review history for this article is available at https://publons.com/publon/10.1002/brb3.70921.

## Supporting information




**Supplementary Materials**: brb370921‐sup‐0001‐SuppMat.pdf

**Supplementary Materials**: brb370921‐sup‐0002‐SuppMat.pdf

**Supplementary Materials**: brb370921‐sup‐0003‐SuppMat.pdf

## Data Availability

The data that support the findings of this study are available on request from the corresponding author. The data are not publicly available due to privacy or ethical restrictions.
