# Supplementary material for: The Role of Willpower in Major Depressive Disorder: An fMRI Study
Source: Brain Behav. 2025 Oct 20;15(10):e70921. doi: 10.1002/brb3.70921 (PMC12537831; doi:10.1002/brb3.70921)
Supplement: Supplementary file 1 — Supplementary Materials: brb370921‐sup‐0001‐SuppMat.pdf [file BRB3-15-e70921-s003.pdf]

TEMPERAMENT AND CHARACTERISTICS INVENTORY

CLONINGER

1. I often try new things just for fun or thrills, even if most people think it is a waste of time.
2. I usually am confident that everything will go well, even in situations that worry most people.
3. I am often moved deeply by a fine speech or poetry.
4. I often feel that I am the victim of circumstances.
5. I can usually accept other people as they are, even when they are very different from me.
6. I believe that miracles happen.
7. I enjoy getting revenge on people who hurt me.
8. Often, when I am concentrating on something, I lose awareness of the passage of time.
9. Often, I feel that my life has little purpose or meaning.
10. I like to help find a solution to problems so that everyone comes out ahead.
11. I could probably accomplish more than I do, but I don't see the point in pushing myself harder than it is necessary to get by.
12. I often feel tense and worried in unfamiliar situations, even when others feel there is little to worry about.
13. I often do things based on how I feel at the moment without thinking about how they were done in the past.
14. I usually do things my own way- rather than giving in to the wishes of other people.
15. I often feel so connected to the people around me that it is like there is no separation between us.
16. I generally don't like people who have different ideas from me.
17. In most situations my natural responses are based on good habits that I have developed.
18. I would do almost anything legal in order to become rich and famous, even if I would lose the trust of many old friends.
19. I am much more reserved and controlled that most people.
20. I often have to stop what I am doing because I start worrying about what might go wrong.
21. I like to discuss my experiences and feelings openly with friends instead of keeping them to myself.
22. I have less energy and get tired more quickly than most people.

23. I am most often called “absent-minded” because I get so wrapped up in what I am doing that I lose track of everything else.
24. I seldom feel free to choose what I want to do.
25. I often consider another person’s feelings as much as my own.
26. Most of the time, I would prefer to do something a little risky (like riding in an automobile over steep hills and sharp turns— rather than having to stay quiet and inactive for a few hours.
27. I often avoid meeting strangers because I lack confidence with people I do not know.
28. I like to please other people as much as I can.
29. I like old “tried and true” ways of doing things much better than trying “new and improved” ways.
30. Usually, I am not able to do things according to their priority of importance to me because of lack of time.
31. I often do things to help protect animals and plants from extinction.
32. I often wish that I was smarter than everyone else.
33. It gives me pleasure to see my enemies suffer.
34. I like to be very organized and set up rules for people whenever I can.
35. It is difficult for me to keep the same interests for a long time because my attention often shifts to something else.
36. Repeated practice has given me good habits that are stronger than most momentary impulses or persuasion.
37. I am usually so determined that I continued to work long after other people would have given up.
38. I am fascinated by the many things in life that cannot be scientifically explained.
39. I have many bad habits that I wish I could break.
40. I often wait for someone else to provide a solution to my problems.
41. I often spend money until I run out of cash or get into debt from using too much credit.
42. I think I will have very good luck in the future.
43. I recover more slowly than most people from minor illnesses or stresses.
44. It wouldn’t bother me to be alone all the time.
45. Often I have unexpected flashes of insight or understanding while relaxing.
46. I don’t care very much whether other people like me or the way I do things.

47. I usually try to get just what I want for myself because it is not possible to satisfy everyone anyway.
48. I have no patience with people who don’t accept my views.
49. I don’t seem to understand most people very well.
50. You don’t have to be dishonest to succeed in business.
51. I sometimes feel so connected to nature that everything seems to be part of one living organism.
52. In conversations I am much better as a listener than as a talker.
53. I lose my temper more quickly than most people
54. When I have to meet a group of strangers, I am more shy than most people
55. I am more sentimental than most people.
56. I seem to have a “sixth sense” that sometimes allows me to know what is going to happen.
57. When someone hurts me in any way, I usually try to get even.
58. My attitudes are determined largely by influences outside my control.
59. Each day, I try to take another step toward my goals.
60. I often wish I was stronger than everyone else.
61. II like to think about things for a long time before I make a decision.
62. I am more hard-working than most people.
63. I often need naps or extra rest periods because I get tired so easily.
64. I like to be of service to others.
65. Regardless of any temporary problem that I have to overcome, I always think it will turn out well.
66. It is hard for me to enjoy spending money on myself, even when I have saved plenty of money.
67. I usually stay calm and secure in situations that most people would find physically dangerous.
68. I like to keep my problems to myself.
69. I don’t mind discussing my personal problems with people whom I have known briefly or slightly.
70. I like to stay at home better than to travel or explore new places.
71. I do not think it is smart to help weak people who cannot help themselves.
72. I cannot have any peace of mind if I treat other people unfairly, even if thay are unfair to me.
73. People will usually tell me how they feel.

74. I often wish I could stay young forever.
75. I am usually more upset than most people by the loss of a close friend.
76. Sometimes, I have felt like I was part of something with no limits or boundaries in time and space.
77. I sometimes feel a spiritual connection to other people that I cannot explain in words.
78. I try to be considerate of other people’s feelings, even when they have been unfair to me in the past.
79. I like it when people can do whatever they want without strict rules and regulations.
80. I would probably stay relaxed and outgoing when meeting a group of strangers, even if I were told they were unfriendly.
81. Usually, I more worried than most people that something might go wrong in the future.
82. I usually think about all the facts in detail before I make a decision.
83. I feel it is more important to be sympathetic and understanding of other people than to be practical and tough-minded.
84. I often feel a strong sense of unity with all the things around me.
85. I often wish I had special powers like Superman.
86. Other people control me too much.
87. I like to share what I have learned with other people.
88. Religious experiences have helped me understand the real purposes of my life.
89. I often learn a lot from people.
90. Repeated practice has allowed me to become good at many things that help me to be successful.
91. I am usually able to get other people to believe me, even when I know that what I am saying is exaggerated or untrue.
92. I need much extra rest, support, or reassurance to recover from minor illnesses or stress.
93. I know there are principles for living that no one can violate without suffering in the long run.
94. I don’t want to be richer than everyone else.
95. I would gladly risk my own life to make the world a better place.
96. Even after thinking about something a long time, I have learned to trust my feelings morehtan my logical reasons.
97. Sometimes, I have felt my life was being directed by a spiritual force greater than any human being.

98. I usually enjoy being mean to anyone who has been mean to me.
99. I have a reputation as someone who is very practical and does not act on emotion.
100. It is very easy for me to organize my thoughts while talking to someone.
101. I often react so strongly to unexpected news that I say or do things that I regret.
102. I am strongly moved by sentimental appeals (like when asked to help crippled children).
103. I usually push myself harder than most people do because I want to do as well as I possibly can
104. I have so many faults that I don’t like myself very much.
105. I have too little time to look for long-term solutions for my problems.
106. I often cannot deal with problems because I just don’t know what to do.
107. I often wish I could stop the passage of time.
108. I hate to make decisions based only on my first impressions.
109. I prefer spending money rather than saving it.
110. I can usually do a good job of stretching the truth to tell a funnier story or to play a joke on someone.
111. Even after there are problems in a friendship, nearly always try to keep it going anyway.
112. If I am embarrassed or humiliated, I get over it very quickly.
113. It is extremely difficult for me to adjust to changes in my usual way of doing things because I get so tensed, tired or worried.
114. I usually demand very good practical reasons before I am willing to change my old ways of doing things.
115. I need a lot of help from other people to train me to have good habits.
116. I think that extra-sensory perception (ESP, like telepathy or precognition) is really possible.
117. I would like to have warm and close friends with me most of the time.
118. I often keep trying the same thing over and over again, even when I have not had much success in a long time.
119. I nearly always stay relaxed and carefree, even when nearly everyone else is fearful.
120. I find sad songs and movies pretty boring.
121. Circumstances often force me to do things against my will
122. it is hard for me to tolerate people who are different from me
123. I think that most things that are called miracles are just chance
124. I would rather be kind than to get revenge when someone hurts me

125. I often become so fascinated with what I'm doing that I get lost in the moment- like I'm detached from time to place.
126. I do not think I have a real sense of purpose for my life
127. I try to cooperate with others as much as possible
128. I am satisfied with my accomplishments, and have little desire to do better
129. I often feel tense and worried in unfamiliar situations, even when others feel there is no danger at all
130. I often follow my instincts, hunches, or intuition without thinking through all the details
131. Other people often think that I am too independent because I wont do what they want
132. I often feel a strong spiritual or emotional connection with all the people around me
133. It is usually easy for me to like people who have different values from me
134. I try to do as little work as possible, even when other people expect more of me
135. Good habits have become "second nature" to me—they are automatic and spontaneous actions nearly all the time
136. I don't mind the fact that other people often know more than I do about something
137. I usually try to imagine myself " in other people's shoes", so I can really understand them
138. Principles like fairness and honesty have little role in some aspects of my life
139. I am better at saving money than most people
140. I seldom let myself get upset or frustrated: when things don't work out, I simply move on to other activities
141. Even when most people feel it is not important, I often insist on things being done in a strict and orderly way.
142. I feel very confident and sure of myself in almost all social situations
143. My friends find it hard to know my feelings because I seldom tell them about my private thoughts
144. I hate to change the way I do things, even if many people tell me there is a new and better way to do it
145. I think it is unwise to believe in things that cannot be explained scientifically
146. I like to imagine my enemies sufferings
147. I am more energetic and tire less quickly than most people
148. I like to pay close attention to details in everything I do

149. I often stop what I am doing because I get worried, even when my friends tell me everything will go well
150. I often wish I was more powerful than everyone else
151. I usually am free to choose what I will do
152. often I become so involved in what I am doing that I forget where I am for a while
153. Members of a team rarely get their fair share.
154. Most of the time I would prefer to do something risky (like hang-gliding or parachute jumping) rather than having to stay quiet and inactive for a few hours
155. Because I spend too much money on impulse, it is hard for me to save money—even for special plans like a vacation
156. I don’t go out of my way to please other people
157. I am not shy with strangers at all
158. I often give in to the wishes of friends
159. I spend most of my time doing things that seem necessary but not really important to me
160. I don’t think that religious or ethical principles about what is right and wrong should have much influence in business decisions
161. I often try to put aside my own judgments so that I can better understand what other people are experiencing
162. Many of my habits make it hard for me to accomplish worthwhile goals.
163. I have made real personal sacrifices in ordering r to make the world a better place- like trying to prevent war, poverty and injustice.
164. I never worry about terrible thing that might happen in the future.
165. I almost never get so excited that I lose control of myself
166. I often give up a job if it takes much longer than I thought it would
167. I prefer to start conversations, rather than waiting for others to talk to me
168. Most of the time I quickly forgive anyone who does me wrong
169. My actions are determined largely by influences outside my control
170. I often have to change my decisions because I had a wrong hunch or mistaken first impression
171. I prefer to wait for someone else to take the lead in getting things done
172. I usually respect the opinions of others

173. I have had experiences that made my role in life so clear to me that I felt very excited and happy
174. It is fun for me to buy things for myself
175. I believe that I have experienced extra – sensory perception myself
176. I believe that my brain in not working properly
177. My behaviour is strongly guided by certain goals that I have set for my life.
178. It is usually foolish to promote the success of other people.
179. I often wish I could live forever
180. I usually like to stay cool and detached from other people
181. I am more likely to cry at a sad movie than most people
182. I recover more quickly than most people from minor illnesses or stress
183. I often break rules and regulations when I think I can get away with it
184. I need much more practice in developing good habits before I will be able to trust myself in many tempting situations
185. I wish other people didn’t talk as much as they do.
186. Everyone should be treated with dignity and respect, even if they seem to be unimportant or bad
187. I like to make quick decisions so I can get o with what has to be done
188. I usually have good luck in whatever I try to do
189. I am usually confident that I can easily do things that most people would consider dangerous (such as driving an automobile fast on a wet or icy road).
190. I see no point in continuing to work on something unless there is a good chance of success
191. I like to explore new ways to do things
192. I enjoy savings money more than spending it on entertainment or thrills
193. Individual rights are more important than the needs of any group
194. I have had personal experiences in which I felt in contact with a divine and wonderful spiritual power
195. I have had moments of great joy in which I suddenly had a clear, deep feeling of oneness with all that exists
196. Good habits make it easier for me to do things the way I want
197. Most people seem more resourceful than I am
198. Other people and conditions are often to blame for my problems

199. It gives me pleasure to help others even if they have treated me badly
200. I often feel like I am a part of the spiritual force on which all life depends
201. Even when I am with friends ,I prefer not to “open up” very much
202. I usually can stay “ on the go” all day without having to push myself
203. I nearly always think about all the facts in detail before I make a decision, even when other people demand a quick decision
204. I am not very good at talking my way out of trouble when I am caught doing something wrong
205. I am more of a perfectionist than most people
206. Whenever something is right or wrong is just a matter of opinion.
207. I think my natural responses now are usually consistent with my principles and long term goals
208. I believe that all life depends on some spiritual order or power that cannot be completely explained
209. I think I would stay confident and relaxed when meeting strangers, even if I were told they are angry at me
210. People find it easy to come to me for help, sympathy , and warm understanding
211. I am slower than most people to get excited about new ideas and activities.
212. I have trouble telling a lie, even when it is meant to spare someone else’s feelings.
213. There are some people I don’t like.
214. I don’t want to be more admired than everyone else.
215. Often when I look at an ordinary thing, something wonderful happens—I get the feeling that I am seeing it fresh for the first time.
216. Most people I know look out only for themselves, no matter who else gets hurt.
217. I usually feel tense and worried when I have to do something new and unfamiliar.
218. I often push myself to the point of exhaustion or try to do more than I really can.
219. Some people think I am too stingy or tight with my money.
220. Reports of mystical experiences are probably wishful thinking.
221. My will power is too weak to overcome very strong temptations, even if I know I will suffer as a consequence.
222. I hate to see anyone suffer.
223. I know what I want to do in life.

224. I regularly take time to consider whether what I am doing is right or wrong.
225. Things often go wrong for me unless I am very careful.
226. If I am feeling upset, I usually feel better around friends than when left alone.
227. I don’t think it is possible for one person to share feelings with someone else who hasn’t had the same experiences.
228. It often seems to other people like I am in another world because I am so completely unaware of things going around me.
229. I wish I were better looking that everyone else.
230. I have lied a lot on this questionnaire
231. I usually stay away from social situations where I would have to meet strangers, even if I am assured that they will be friendly
232. I love the blooming of flowers in the spring as much as seeing an old friend again
233. I usually look at a difficult situation as a challenge or opportunity
234. People involved with me have to learn how to do things my way
235. Dishonesty only causes problems if you get caught
236. I usually feel much more confident an energetic than most people, even after minor illnesses or stress.
237. I like to read everything when I am asked to sign any papers.
238. When nothing new is happening, I usually start looking for something that is thrilling or exciting.
239. Sometimes I get upset.
240. Occasionally I talk about people behind their backs.
