# Supplementary material for: The Role of Willpower in Major Depressive Disorder: An fMRI Study
Source: Brain Behav. 2025 Oct 20;15(10):e70921. doi: 10.1002/brb3.70921 (PMC12537831; doi:10.1002/brb3.70921)
Supplement: Supplementary file 2 — Supplementary Materials: brb370921‐sup‐0002‐SuppMat.pdf [file BRB3-15-e70921-s002.pdf]

Türk Psikiyatri Dergisi 2005; 16(3):190-204

Mizaç ve Karakter Envanteri'nin Türkçe Formunun Faktör Yapısı, Geçerlik ve Güvenilirliği

Dr. Haluk ARKAR[1], Dr. Oya SORİAS[2], Dr. Zeliha TUNCA[3], Dr. Cennet ŞAFAK[4], Dr. Tunç ALKIN[3], Dr. Berna BİNNUR AKDEDE[1], Dr. Seda ŞAHİN[5], Dr. Yıldız AKVARDAR[1], Dr. Özden SARI[6], Dr. Ayşegül ÖZERDEM[1], Dr. Can CİMİLLİ[3]

ÖZET

Amaç: Cloninger, kişiliğin yapısını ve gelişimini tanımlamak için, genel bir psikobiyolojik kuram geliştirmiştir. Bu model, dört mizaç boyutu (yenilik arayışı, zarardan kaçınma, ödül bağımlılığı ve sebat etme) ve üç karakter boyutunu (kendini yönetme, iş birliği yapma ve kendini aşma) içermektedir. Kişinin kendi doldurduğu bir ölçek olan TCI (Temperament and Character Inventory) mizaç ve karakter boyutlarını ve bunların 25 altboyutunu ölçmek üzere geliştirilmiştir. Bu çalışmanın amacı, TCI'nin psikometrik özelliklerini bir Türk örnekleminde sınamaktı.

Yöntem: Çalışmanın örneklemi, hayatlarının hiç bir döneminde herhangi bir psikolojik/psikiyatrik tedavi almamış 470 sağlıklı gönüllü ve 544 psikiyatri hastasından oluştu.

Bulgular: Ayrı ayrı olarak araştırılan, mizaç ve karakter boyutlarının faktör yapıları, keşfetmekten heyecan duyma (NS1), sebat etme ve kendini kabullenme (SD4) hariç, öngörülen kuramsal yapı ile uyuşmaktaydı. Ölçeklerin iç tutarlılığı genel olarak kabul edilebilir düzeydeydi, ancak, ödül bağımlılığı (0.55) ve sebat etme'de (0.56) zayıftı. Korelasyonlar ve grup karşılaştırmaları TCI'nin yapı geçerliğine katkıda bulunmaktadır.

Sonuç: Bu çalışmanın bulguları, TCI'nin Türkçe versiyonunun tatmin edici psikometrik özelliklere sahip olduğunu doğrulamaktadır. TCI, hem klinik uygulamalarda hem de araştırmalarda kişiliği değerlendirmek için kullanılabilecek yararlı bir araç olarak görünmektedir.

Anahtar Sözcükler: Mizaç, Karakter, Kişilik, TCI

SUMMARY: Factorial Structure, Validity, and Reliability of the Turkish Temperament and Character Inventory

Objective: To assess the factorial structure, reliability and validity of the Turkish version of the Temperament and Character Inventory (TCI), a 240-item, self-report, paper-and-pencil test, and true-false format inventory based on Cloninger's psychobiological model of personality. It measures the four higher-order temperament dimensions and three character dimensions

Method: Using samples consisting of 470 healthy volunteers and 544 psychiatric patients, psychometric features were explored.

Results: The internal consistency of the scales was good (Cronbach alpha coefficients between 0.68 and 0.84), but weak for Reward dependence (0.55) and Persistence (0.56). The factor structures of the temperament and character dimensions, explored separately, were in agreement with the hypothesized constructs, except for the scales NS1 (Novelty Seeking 1 = exploratory excitability) and SD4 (Self-directedness 4 - self-acceptance). The present study also confirmed that the TCI scales were weakly related among themselves. On the whole, psychiatric patients had higher harm avoidance and lower self-directedness, persistence, cooperativeness, and self-transcendence scores than the normal subjects. Gender differences were also found for different dimensions.

Conclusion: The findings of this study suggest that the TCI can be applied in the investigation of psychiatric and normal populations.

Key Words: Temperament, Character, Personality, TCI

[6]Uzm., Psk., [1]Doç.,[7]Doç., [3]Prof., Dokuz Eylül Ü Psikiyatri AD., [2]Prof., Ege Ü Psikoloji Bl., [4]Uzm., Psk., Konak Ekrem Hayri Üstündağ Doğum Hastanesi, [5]Psk., Alsancak Özel Eğitim Merkezi, İzmir.

GİRİŞ

Cloninger, kişiliğin yapısını ve gelişimini tanımlamak için, genel bir psikobiyolojik kuram geliştirmiştir (Cloninger 1987, Cloninger ve ark. 1993). Bu model, genetik olarak birbirinden bağımsız, yaşam boyunca orta düzeyde durağan, sosyokültürel etkiler karşısında değişmez oldukları ve algısal bellekte kavramöncesi yanlılıkları içerdikleri varsayılan dört mizaç boyutu (yenilik arayışı, zarardan kaçınma, ödül bağımlılığı ve sebat etme) ve yetişkinlikte olgunlaştıkları ve kendilik kavramları hakkında içgörü öğrenmesi ile kişisel ve sosyal etkinliği etkiledikleri varsayılan üç karakter boyutunu (kendini yönetme, iş birliği yapma ve kendini aşma) içermektedir.

Mizaç boyutları, yenilik, tehlike veya ceza ve ödül'e yanıt olarak çağrışımsal öğrenmedeki bireysel farklılıklara göre tanımlanmaktadır. Mizaç faktörlerinden biri, yenilik arayışı (novelty seeking), yeniliğe yanıt olarak sık araştırıcı aktivite, dürtüsel karar verme, ödül alma olasılığı belirdiğinde aşırılık ve taşkınlık, çabuk kolay sinirlenme ve engellenmeden aktif kaçınma gibi davranışların etkinleşmesindeki veya başlamasındaki bir kalıtsal yanlılık, eğilim olarak görülebilir. İkinci mizaç faktörü, zarardan kaçınma (harm avoidance), gelecekte olabilecek sorunlar için kötümser endişeler, belirsizlik korkusu ve yabancılardan utanma gibi pasif kaçıngan davranışlar ve kolayca yorulma gibi davranışların ketlenmesinde veya durdurulmasındaki bir kalıtsal yanlılık, eğilim olarak görülebilir. Üçüncü mizaç faktörü, ödül bağımlılığı (reward dependence), kendini aşırı duygusallık, sosyal bağlılık ve diğerlerinin onayına bağımlılık gibi gösteren davranışların devamlılığı ve sürekliliğindeki bir kalıtsal yanlılık, eğilim olarak görülebilir. Önceleri ödül bağımlılığının bileşenlerinden biri olarak düşünülen, dördüncü mizaç faktörü, sebat etme (persistence), engellenme, yorgunluk ve aralıklı pekiştirilmeye rağmen davranışın sürekliliğindeki bir kalıtsal yanlılık, eğilim olarak görülebilir. Mizaç boyutları özgül bir merkezi monoaminerjik sistem aktivitesi ile korelasyon göstermektedir: yenilik arayışı düşük dopaminerjik aktivite ile, zarardan kaçınma yüksek serotonerjik aktivite ile ve ödül bağımlılığı düşük noradrenerjik aktivite ile (Cloninger, 1986; 1987).

Karakter, içgörü öğrenmesi ve kendimiz, diğer insanlar ve diğer nesneler hakkındaki kavramlarımıza dayalı olarak, gönüllü hedefler ve değerlerdeki bireysel farklılıklara karşılık gelmektedir. Kendilik kavramlarının üç yönü; bir kişinin kendini ne ölçüde bir otonom kişi (Kendini yönetme -Self-directedness), toplumun entegre bir parçası (İş birliği yapma-Cooperativeness) ve evrenin bütünleşmiş bir parçası (Kendini aşma-Self-transcendence) olarak tanımladığına göre farklılaşmaktadır (Cloninger, Svrakic ve Przybeck, 1993).

Mizaç, duygulara dayalı becerilerin ve alışkanlıkların bütünleştirilmesinin altında yatan duyumsama, bağlantı kurma ve motivasyon süreçlerine karşılık gelmektedir. Karakter ise, kavramsal öğrenmeye dayanan sembolizasyon ve soyutlaştırma süreçlerine karşılık gelmektedir. Özgül olarak, mizaç ve karakter, iki tip bellek ve öğrenmeye; önermesel (propositional) ve işlemsel (procedural), dayalı olarak kavramsallaştırılabilir. Mizaç (kişiliğin "duygusal özü"), kortikositriatolimbik sistem tarafından düzenlenen işlemsel belleği içermektedir. Karakter (kişiliğin "kavramsal özü"), sembolizasyon ve soyutlama yüksek bilişsel işlevlerini içeren önermesel bellek ile ilişkilidir. Bu iki temel bellek ve öğrenme sistemi işlevsel olarak ayrıştırılabilir. Örneğin, striatal lezyonlarla karakterize Parkinson hastalığı olan kişiler, işlemsel öğrenmede bozulmalar gösterirken, önermesel öğrenmede göstermezler.Tam tersi olarak, medial temporal lob'daki lezyonlarla karakterize amnestik sendromlu kişiler, önermesel öğrenmede bozulmalar gösterirler, işlemsel öğrenmede bozulma göstermezler (Cloninger ve Svrakic, 2000).

Cloninger ve arkadaşları, Cloninger'in psikobiyolojik kişilik kuramına dayalı olarak, kişiliğin yedi boyutunu ölçen, Mizaç ve Karakter Envanteri (Temperament and Character Inventory; TCI) adı verilen kapsamlı bir ölçüm aracı geliştirdiler (Cloninger ve ark., 1993, 1994). Mizaç ve Karakter Envanteri dört mizaç ve üç karakter özelliğini değerlendirmektedir. Psikolojik ve psikiyatrik araştırma ve uygulamaların farklı alanlarında uluslararası olarak kullanımı son 10 yıl içinde büyük bir artış göstermiştir. Doğru/yanlış olarak doldurulan 240 maddeden oluşan, 30-45 dakika arasında doldurulabilen, 17 yaş ve üzeri kişilere uygulanabilen kişinin kendi doldurduğu (self report) bir ölçektir.

Bu çalışma TCI'nın Türkiye'de kullanımını sınamak amacıyla yürütülmüştür. Psikometrik özellikler (iç tutarlılık, geçerlik ve faktör yapısı) psikiyatri hastaları ve sağlıklı gönüllülerin oluşturduğu bir örneklemde araştırıldı.

TABLO 1. Örneklemin Demografik Özellikleri.

	Örneklem					
	Hasta (s=544)		Normal (s=470)		Toplam (s=1014)	
	S	%	S	%	S	%
Cinsiyet						
Kadın	365	67	332	70.6	697	68.7
Erkek	179	33	138	29.4	317	31.3
Yaş						
17-27	159	29.2	157	33.4	316	31.2
28-37	127	23.3	142	30.2	269	26.5
38-47	118	21.7	116	24.7	234	23.1
48-57	84	15.4	35	7.4	119	11.7
58+	56	10.3	20	4.3	76	7.5
Yerleşim yeri						
Köy	1	0.2	12	2.6	13	1.3
Kasaba	19	3.5	37	7.9	56	5.5
Kent	109	20	162	34.5	271	26.7
Büyükkent	415	76.3	259	55.1	674	66.5
Medeni durum						
Bekar	189	34.7	186	39.6	375	37
Nişanlı	4	0.7	9	1.9	13	1.3
Evli	294	54	236	50.2	530	52.3
Boşanmış	33	6.1	20	4.3	53	5.2
Dul	24	4.4	19	4	43	4.2
Eğitim durumu						
İlkokul	97	17.8	37	7.9	134	13.2
Ortaokul	63	11.6	31	6.6	94	9.3
Lise	259	47.6	229	48.7	488	48.1
Üniversite	119	21.9	165	35.1	284	28
Lisansüstü	6	1.1	8	1.7	14	1.4

S Sayı

YÖNTEM

Örneklem

Çalışmanın örneklemi, Dokuz Eylül Üniversitesi Hastanesi Psikiyatri Kliniği'nde ayaktan veya yatarak tedavi olan veya tedavi için ilk kez başvuran 544 psikiyatrik rahatsızlığı olan gönüllü ile hayatlarının hiç bir döneminde herhangi bir psikolojik/psikiyatrik tedavi almamış 470 sağlıklı gönüllünün oluşturduğu iki gruptan oluşmuştur. Grupların demografik özellikleri Tablo 1'de verilmiştir.

Hayatlarının hiç bir döneminde herhangi bir psikolojik/psikiyatrik tedavi almamış olma kriterine göre çalışmaya alınan sağlıklı gönüllülerin oluşturduğu normal grubunun yaş ortalaması 34.1, standart sapması 11.5 (yaş aralığı 18–72) idi. Çalışmaya katılım gönüllülük temeline dayanıyordu. Örneklem ulaşılabilirliğe göre seçildi ve ücret ödeme veya katılımcının kendi istekliliği gibi yanlılıklar seçimi etkilemedi. Normal grubunda 332 kadın ve 138 erkek vardı. Yaş dilimi açısından, en kalabalık grup 17-27 yaş grubuydu. 48-57 ve 58 – üstü yaş dilimlerinde kişi sayısı nisbeten azdı (48–57

TABLO 2. Yaş Grupları ve Cinsiyet'e Göre TCI Ölçeklerinin Ortalama, Standart Sapma ve Cronbach Alfaları.

			Yaş										
			17-27		28-37		38-47		48-57		58+		
Boyut			Ort.	SS	Ort.	SS	Ort.	SS	Ort.	SS	Ort.	SS	α
YA	Hasta	Kadın	20.63	4.77	17.55	4.73	17.25	4.86	16.64	3.48	15.72	4.72	0.68
		s	105		88		79		61		32		
		Erkek	18.65	6.30	18.34	5.19	19.46	6.83	17.96	4.27	16.54	4.97	
		s	54		39		39		23		24		
	Normal	Kadın	20.29	5.02	18.79	4.17	18.23	4.93	17.92	4.82	14.63	3.44	
		s	112		104		80		25		11		
		Erkek	18.38	4.42	17.95	4.24	17.36	4.47	13.90	4.77	13.89	3.76	
		s	45		38		36		10		9		
ZK	Hasta	Kadın	23.50	5.74	22.85	7.13	22.04	6.90	21.16	5.47	21.13	4.44	0.84
		Erkek	21.44	6.96	23.31	5.34	18.44	6.41	18.61	7.00	19.46	5.55	
	Normal	Kadın	15.70	5.62	16.28	6.07	17.01	5.74	17.28	7.93	20.00	5.00	
		Erkek	15.27	5.61	15.66	6.06	17.22	6.24	19.00	6.77	16.78	5.14	
ÖB	Hasta	Kadın	14.32	3.55	14.34	2.93	14.10	3.00	14.62	3.18	13.44	2.47	0.55
		Erkek	12.85	2.90	12.05	2.86	13.82	2.34	13.52	2.94	13.50	2.83	
	Normal	Kadın	14.62	3.40	14.03	3.45	14.65	3.11	14.12	3.50	13.45	2.02	
		Erkek	12.62	3.26	13.34	3.64	12.92	3.31	11.90	3.11	13.56	3.05	
SE	Hasta	Kadın	4.42	1.76	4.86	1.86	5.05	1.62	5.26	1.95	4.78	1.84	0.56
		Erkek	4.41	2.07	4.08	1.97	5.23	1.66	4.26	2.14	4.67	1.63	
	Normal	Kadın	4.88	2.13	5.23	1.71	5.31	1.82	5.28	1.62	4.73	1.49	
		Erkek	4.96	2.17	6.11	1.71	4.83	1.90	4.50	1.96	5.11	1.90	
KY	Hasta	Kadın	22.83	6.78	24.66	7.89	24.75	6.57	26.23	6.01	27.66	5.24	0.82
		Erkek	24.30	7.09	25.05	8.35	26.90	7.37	28.17	7.39	27.13	7.47	
	Normal	Kadın	27.90	6.29	28.56	6.68	27.78	5.63	28.56	6.97	28.82	3.34	
		Erkek	27.33	6.77	28.55	5.56	27.61	6.01	27.20	6.84	33.44	7.57	
İY	Hasta	Kadın	25.42	7.18	27.57	5.45	27.41	5.70	27.89	5.26	27.03	4.90	0.80
		Erkek	25.96	6.83	24.49	7.98	26.79	5.26	26.70	7.57	27.13	5.67	
	Normal	Kadın	27.88	6.20	28.30	5.57	28.98	5.33	28.76	6.11	29.55	2.88	
		Erkek	26.29	6.59	27.71	5.75	26.42	6.05	24.80	4.37	29.00	6.16	
KA	Hasta	Kadın	16.37	5.38	16.65	6.04	17.81	6.01	19.44	6.15	16.63	5.78	0.80
		Erkek	16.83	6.35	16.18	5.67	17.23	6.19	17.83	5.57	17.96	6.17	
	Normal	Kadın	18.12	5.57	17.95	5.72	18.58	5.96	17.48	5.54	18.82	5.91	
		Erkek	17.98	6.02	18.45	5.01	18.03	4.66	18.30	5.40	15.11	5.23	

Ort: Ortalama, SS: Standart Sapma, YA: Yenilik Arayışı, ZK: Zarardan Kaçınma, ÖB: Ödül Bağımlılığı, SE: Sebat Etme, KY: Kendini Yönetme, İY: İşbirliği Yapma, KA: Kendini Aşma.

yaş dilimi tüm normal grubunun % 7'sini ve 58 ve üstü olanlar ise %4.3'ünü oluşturuyorlardı). Yarısı (%50.2) evli kimselerdi. Medeni durum açısından ikinci büyük yüzdeyi (%39.6) bekarlar oluşturdu.

TABLO 3. TCI Ölçeklerinin Bırbirleriyle ve Yaşla Korelasyonları.

	YA	ZK	ÖB	İY	KY	İY	KA
ZK	-0.23						
ÖB	0.13	-0.09					
SE	-0.17	-0.23	0.12				
KY	-0.14	**-0.49**	0.11	0.18			
İY	-0.08	-0.26	**0.41**	0.15	**0.45**		
KA	0.08	-0.11	0.14	0.22	-0.21	0.07	
YAŞ	-0.24	0.03	-0.01	0.05	0.11	0.06	0.04

ZK: Zarardan Kaçınma, YA: Yenilik Arama, ÖB: Ödül Bağımlılığı, SE: Sebat Etme, KY: Kendini Yönetme, İY: İşbirliği Yapma, KA: Kendini Aşma.
Not. 0.40'ın üzeri korelasyonlar koyu olarak verilmiştir.

Normal grubunun büyük çoğunluğu kent kökenliydi (%55'i büyükkent ve %35'i kent). Normal grubunda eğitim seviyesi olarak ağırlık %48.7 ile lise mezunlarındaydı. Lise mezunlarını %35 ile üniversite mezunları takip etti.

Dokuz Eylül Üniversitesi Hastanesi Psikiyatri Kliniği'nde ayaktan veya yatarak tedavi olan veya tedavi için ilk kez başvuran 544 psikiyatri hastası, hasta grubunu oluşturdu. 365 kadın ve 179 erkek'ten oluşan hasta grubunun yaş ortalaması 37.6, standart sapması 13.9 (yaş aralığı; 18–80) idi. Psikiyatri hastalarının büyük bir çoğunluğu (%93) ayaktan tedavi gören hastalardı. Yatarak tedavi olan yalnızca 39 hasta vardı. Hasta grubunun % 35'ini, psikiyatrik/psikolojik sorunları için tedavi aramak amacıyla ilk kez başvuranlar oluşturdu. Hastaların %65'i ise bir süredir psikiyatrik tedavilerini sürdüren kişilerdi. Organik beyin bozukluğu tanısı olan, zeka geriliği olan ve testleri güvenilir bir şekilde dolduramayacak kadar konfuze, ajite ve açık bir şekilde psikotik olan hastalar çalışmaya alınmadı. Psikiyatri hastalarının Eksen I tanıları kendilerini muayene eden ve tedavilerini sürdüren psikiyatristler tarafından konuldu. DSM-IV tanı ölçütlerine göre; duygudurum bozukluğu (% 44.7); anksiyete bozukluğu (%28.1); psikotik bozukluk (%9.4); uyum bozukluğu (%7.2); yukarıda belirtilmeyen diğer Eksen I tanıları (%10.6) konmuştur. Hasta grubu da normal grubu gibi çoğunlukla kadınlardan (%67 ve %70.6) oluştu. Yaş dilimi açısından, normal grubunda olduğu gibi, en kalabalık grup 17-27 yaş grubuydu. Yarısı (%54) evli kimselerdi. Medeni durum açısından ikinci büyük yüzdeyi (%35) bekarlar oluşturdu. Hasta grubunda her eğitim düzeyinden katılımcı bulunmakla birlikte, ağırlık %47 ile lise mezunlarındaydı.

Araç

Mizaç ve Karakter Envanteri (Temperament and Character Inventory; TCI): Envanter, doğru/yanlış olarak doldurulan 240 maddeden oluşan, kendi bildirim tarzı bir ölçektir. 12'si mizaç (Keşfetmekten heyecan duyma (YA1, 11 madde), Dürtüsellik (YA2, 10 madde), savurganlık (YA3, 9 madde), Düzensizlik (YA4, 10 madde), Beklenti Endişesi (ZK1, 11 madde), Belirsizlik korkusu (ZK2, 7 madde), Yabancılardan çekinme (ZK3, 8 madde), Çabuk yorulma (ZK4, 9 madde), Duygusallık (ÖB1, 10 madde), Bağlanma (ÖB3, 8 madde), Bağımlılık (ÖB4, 6 madde), Sebat etme (SE, 8 madde) ve 13'ü karakter (Sorumluluk alma (KY1, 8 madde), Amaçlılık (KY2, 8 madde), Beceriklilik (KY3, 5 madde), Kendini kabullenme (KY4, 11 madde), Uyumlu ikincil huylar (KY5, 12 madde), Sosyal onaylama (İY1, 8 madde), Empati duyma (İY2, 7 madde), Yardımseverlik (İY3, 8 madde), Acıma (CO4, 10 madde), Erdemlilik (İY5, 9 madde), Kendilik kaybı (KA1, 11 madde), Kişiler ötesi özdeşim (KA2, 9 madde) ve Manevi kabullenme (KA3, 13 madde) olmak üzere, 25 altölçekten (lower-order) ve 7 (higher-order) ölçekten oluşmaktadır. Ölçekler altölçeklerin toplamından oluşmaktadır (örneğin, YA = YA1 + YA2 + YA3 + YA4). TCI çok çeşitli dillere çevrilmiş ve çok çeşitli kültürlerde psikometrik özellikleri sınanmıştır (Brandstrom ve arkadaşları (1998) tarafından İsveç'te, Pelissolo ve Lepine (2000) tarafından Fransa'da, Duijsens ve arkadaşları (2000) tarafından Hollanda'da, Gutierrez ve arkadaşları (2001) tarafından İspanya'da, Tanaka ve arkadaşları (1997) tarafından Japonya'da, Hansenne ve arkadaşları (2001) tarafından Belçika'da, Richter ve arkadaşları (1999) tarafından Almanya'da, Waller ve arkadaşları (1991) tarafından İngiltere'de,

TABLO 4. TCI Mizaç Altölçeklerinin Faktör Yapısı (Pattern Matrix).

Mizaçlar	Faktör 1	Faktör 2	Faktör 3
YA1	**-.57**	.19	**.41**
YA2	.11	**.68**	-.13
YA3	-.10	**.67**	.21
YA4	-.12	**.68**	-.01
ZK1	**.77**	.04	.22
ZK2	**.74**	-.14	.13
ZK3	**.75**	.01	-.17
ZK4	**.75**	.23	.09
ÖB1	.12	-.08	**.63**
ÖB3	-.28	.17	**.66**
ÖB4	.16	-.09	**.47**
SE	-.32	**-.55**	.18

YA: Yenilik Arama, ZK: Zarardan Kaçınma, ÖB: Ödül Bağımlılığı, SE: Sebat Etme.

Kozeny ve Höschl (1999) tarafından Çek Cumhuriyetinde ve Sung ve arkadaşları (2002) tarafından Kore'de). Psikiyatrik araştırmanın farklı alanlarındaki bir çok çalışmada uluslararası olarak kullanılmıştır ve kullanılagelmektedir. Köse tarafından Türkçe'ye çevrilmiş ve tersine çevirme işlemi Sayar tarafından yapılmış ve bu çeviri Cloninger tarafından onaylanmıştır. Ölçeğin güvenilirlik ve geçerliliğine dair ilk bulgular Köse ve arkadaşları (2004) tarafından bildirilmiştir.

İşlem

Katılımcılara Mizaç ve Karakter Envanteri (TCI) tek tek uygulandı. Envanterin başında gerekli yönergeler verildi. Kullanılan aracın ilk sayfasında çalışma ile ilgili genel bir giriş, çalışmacıların adresi ve katılımın gönüllülük esasına dayandığına dair bilgi vardı. Bu sayfa, aynı zamanda, demografik özelliklere ilişkin soruları da içeriyordu. İstatistiksel analizlere başlamadan önce, tüm değişkenler ayrı ayrı olarak, verilerin doğru bir şekilde girilip girilmediği, boş değerler olup olmadığı, çok değişkenli analizlerin varsayımlarına ve dağılımlarına uyup uymadıkları açısından SPSS FREQUENCIES ile sınandı. Olgularda boş değer yoktu. Mahalanobis uzaklığı kriteri ($p < 0.001$ ile) kullanılarak, olgular arasında çıkıntı değer (outlier) bulunmadı. Normallik ve doğrusallık kontrol edildi ve tatmin edici bulundu.

TCI ölçek ve altölçeklerinin iç tutarlılık güvenirliği, Cronbach alfa yöntemi kullanılarak değerlendirildi. TCI ölçeklerinin arasındaki ilişkinin derecesini görmek için, birbirleriyle olan korelasyonları hesaplandı. TCI kişilik boyutları ile yaş arasındaki ilişkileri değerlendirmek için, tüm örneklemde TCI ölçekleri ile yaş arasında Pearson momentler çarpımı korelasyon katsayıları hesaplandı. Psikiyatri hastaları ve sağlıklı gönüllülerin oluşturduğu iki grup, faktör analizinin gerektirdiği örneklem büyüklüğüne ulaşmak için birleştirildi (N= 1014). Ölçeğin faktör yapısını görmek için, verilere "Kaiser normalizasyonu ve oblimin" dönüştürmesine göre Temel Bileşenler (Principal Components) faktör analizi yapıldı. Psikiyatri hastaları ile sağlıklı normaller arasındaki farklılıkları test etmek için, 2 (psikiyatri hastaları vs. sağlıklı normaller) x 2 (kadın vs. erkek) çok değişkenli varyans analizi (MANOVA) yapıldı. Psikiyatri hastası ve sağlıklı normal gruplarına ait olmanın yordayıcıları olarak, TCI'in 25 alt ölçeği kullanılarak diskriminant analizi gerçekleştirildi. Analizler, SPSS'in uygun alt programları kullanılarak gerçekleştirildi.

BULGULAR

TCI ölçeklerinin, psikiyatri hastaları ve sağlıklı gönüllülerin, cinsiyete ve beş yaş grubuna (17-27, 28-37, 38-47, 48-57 ve 58 ve üstü) göre ortalama ve standart sapmaları Tablo 2'de verilmiştir

Güvenilirlik

TCI ölçek ve altölçeklerinin iç tutarlılık güvenilirliği, Cronbach alfa yöntemi kullanılarak hesaplandı. TCI ölçeklerinin Cronbach alfa değerleri Tablo 2'de verilmiştir. TCI ölçeklerinin Cronbach alfa değerleri, mizaç ölçeklerinde 0.55 ile 0.84 ve

TABLO 5. TCI Karakter Altölçeklerinin Faktör Yapısı (Pattern Matrix).

Karakterler	Faktör 1	Faktör 2	Faktör 3
KY1	.06	-.31	**.67**
KY2	.03	.02	**.76**
KY3	-.11	.04	**.83**
KY4	**.47**	**-.44**	-.04
KY5	.11	.02	**.71**
İY1	**.68**	.02	.09
İY2	**.44**	.19	.25
İY3	**.60**	.07	.10
İY4	**.82**	.05	-.15
İY5	**.60**	-.05	.03
KA1	-.06	**.76**	-.10
KA2	.26	**.78**	.02
KA3	.00	**.70**	-.02

KY: Kendini Yönetme, İY: İşbirliği Yapma, KA: Kendini Aşma.

karakter ölçeklerinde 0.80 ile 0.84 arasındadır. En düşük Cronbach alfa katsayıları Ödül Bağımlılığı (0.55) ve Sebat etme'de (0.56) bulunmuştur. Karakter ölçeklerinin üçü de 0.80 ve üzeri değerdedir.

TCI altölçeklerinin Cronbach alfa değerleri (YA1, .49; YA2, .50; YA3, .67; YA4, .31; ZK1, .64; ZK2, .61; ZK3, .71; ZK4, .69; ÖB1, .49; ÖB3, .58; ÖB4, 0.31; KY1, .64; KY2, .60; KY3, .51; KY4, .69; KY5, .57; İY1, .58; İY2, .36; İY3, .35; İY4, .79; İY5, .39; KA1, .63; KA2, .64; KA3, .70), genel olarak, İş birliği yapma ölçeği hariç, her bir ölçek ile tutarlıdır. İş birliği yapma ölçeğinin Cronbach alfa değeri 0.80 iken, altölçekleri çok geniş bir varyasyon göstermiştir. Yardımseverlik (İY3) altölçeği en düşük Cronbach alfa değerine sahiptir (0.35). Yine, empati duyma (İY2) altölçeğinin (0.36) ve Erdemlilik (İY5) altölçeğinin (0.39) Cronbach alfa değerleri oldukça düşüktür. Zarardan Kaçınma'nın altölçekleri, nisbeten durağan (0.64 ile 0.71 arasında değişen) Cronbach alfa değerleri gösterdiler. Kendini Yönetme altölçekleri, 0.51 olan beceriklilik (KY3) altölçeği hariç, 0.57 ile 0.69 değerleri arasındadır. Yenilik Arama altölçekleri de, İşbirliğine Yatkınlık altölçeklerine benzer bir şekilde geniş bir varyasyon göstermiştir. Özet olarak, 24 altölçekten 11'i 0.60 değerinin altındadır.

TCI Ölçeklerinin Birbirleriyle Korelasyonları

TCI ölçeklerinin arasındaki ilişkinin derecesini görmek için, birbirleriyle olan korelasyonları hesaplandı (bakınız Tablo 3). TCI mizaç ve karakter ölçeklerinin birbirleriyle korelasyonlarına bakıldığında, Kendini Yönetme, Zarardan Kaçınma ile negatif yönde anlamlı ilişki ($r = -0.49$) gösterdi. İş Birliği Yapma, hem Ödül Bağımlılığı ($r = 0.41$) hem de Kendini Yönetme ($r = 0.45$) ile pozitif yönde anlamlı ilişki gösterdi. Yani, kendini yönetmenin düşük oluşu, zarardan kaçınmanın yüksek oluşunu beraberinde getirmektedir. İş birliği yapmanın yüksek oluşu ise, hem ödül bağımlılığının hem de kendini yönetmenin yüksek oluşunu beraberinde getirmektedir. Ölçekler arasındaki diğer korelasyonların hepsi zayıf ilişki (0.26 ile 0.07 arasında) gösterdi.

TCI Ölçeklerinin Yaş ile Korelasyonları

Tablo 3'de görüldüğü gibi, yaş, Yenilik Arayışı (YA, $r = -0.24$, $p < 0.001$) ile anlamlı seviyede negatif ve Kendini Yönetme (KY, $r = 0.11$, $p < 0.05$) pozitif korelasyon gösterdi. Yaş arttığında, yenilik arayışı azalmakta buna karşılık kendini yönetme artmaktadır.

Faktör Yapısı

Verilere "Kaiser normalizasyonu ve oblimin" dönüştürmesine göre Temel Bileşenler (Principal Components) faktör analizi yapıldı. Psikiyatri hastaları ve sağlıklı gönüllülerin oluşturduğu iki grup, faktör analizinin gerektirdiği örneklem büyüklüğüne ulaşmak için birleştirildi ($N = 1014$). TCI'nin 25 altölçeğinin birlikte analize girdiği, yedi-faktörlü çözüme göre ortaya çıkan örüntü basit değildir. Yedi-faktörlü çözümün öngörülen TCI faktör

TABLO 6. Anlamlı MANOVA Sonuçları.

Kaynak	Faktör	F	df	P
Yenilik Arayışı	Grup* cinsiyet	7.91	1,1010	<0.01
Zarardan Kaçınma	Grup	148.43	1,1010	<0.001
	Cinsiyet	6.01	1,1010	<0.05
	Grup*cinsiyet	3.88	1,1010	<0.05
Ödül Bağımlılığı	Cinsiyet	37.50	1,1010	<0.001
Sebat Etme	Grup	14.39	1,1010	<0.001
Kendini Yönetme	Grup	38.43	1,1010	<0.001
İş Birliği Yapma	Grup	6.98	1,1010	<0.01
	Cinsiyet	8.86	1,1010	<0.005
Kendini-Aşma	Grup	5.02	1,1010	<0.05

yapısını tekrar ettiğini söylemek güçtür. Yalnızca Yenilik Arayışı, Zarardan Kaçınma ve Kendini Aşma bir faktöre yük vermişlerdir. Sebat Etme ölçeği, en azından bu örneklemde, kesin olarak kişilik modelinin ayrı bir boyutu değildir. Mizaç ve karakter boyutları aynı faktörlerde binişmektedir. Kendini Yönetme ve İş Birliği Yapma tam olarak tanımlanamamıştır.

Yedi-faktörlü çözüme göre ortaya çıkan faktör yapısı Cloninger'in kişilik modeli ile tam bir tutarlılık göstermediği için, verilere, mizaç ve karakter altölçekleri için ayrı ayrı olarak, "oblimin" dönüştürmesine göre Temel Bileşenler faktör analizi tekrarlandı. Ortaya çıkan faktörler Tablo'4 ve 5'de verilmiştir. Genel olarak, hem mizaç hem de karakter boyutlarında, özgül bir boyutla ilişkili olan altölçekler en yüksek faktör yüklerini tek bir boyuta verdiler.

Mizaçlarda, üç faktörlü çözüme göre, Zarardan Kaçınma ve Ödül Bağımlılığı oldukça kuvvetli gözüktü. Yenilik Arayışı faktöründe, Dürtüsellik (YA2), Savurganlık (YA3), Düzensizlik (YA4) altölçekleri tutarlı bir şekilde yük verirken, Keşfetmekten heyecan duyma (YA1) altölçeği yalnızca çok zayıf bir yük sağladı. Keşfetmekten heyecan duyma (YA1), daha çok, negatif olarak Faktör 1'e (Zarardan Kaçınma) ve pozitif olarak Faktör 3'e (Ödül Bağımlılığı) yük verdi. Sebat etme en yüksek faktör yükünü negatif olarak Faktör 2'ye (Yenilik Arayışı) verdi. Üç faktör, toplam varyansın %24, %15 ve %11'ini (kümülatif olarak %50'sini) açıkladılar. TCI mizaç altölçeklerinin faktör yapısı Tablo 4'de görülebilir.

Karakterlerde, üç faktörlü çözüme göre, İş birliği Yapma ve Kendini Aşma oldukça sağlam gözüktü. Kendini Yönetme faktöründe, Sorumluluk alma (KY1), Amaçlılık (KY2), Beceriklilik (KY3) ve Uyumlu ikincil huylar (KY5) altölçekleri tutarlı bir şekilde yük verirken, Kendini kabullenme (KY4) altölçeği yük vermedi. Kendini kabullenme (KY4), daha çok, pozitif olarak Faktör 1'e (İş birliği Yapma) ve negatif olarak Faktör 2'ye (Kendini Aşma) yük verdi. Üç faktör, toplam varyansın %27, %16 ve %10'nunu (kümülatif olarak %53'ünü) açıkladılar. TCI karakter altölçeklerinin faktör yapısı Tablo 5'de görülebilir.

Grup ve Cinsiyet Karşılaştırmaları

TCI'a ilişkin olası grup ve cinsiyet farklılıklarını incelemek için, 2 (psikiyatri hastaları vs. sağlıklı normaller)* 2 (kadın vs. erkek) çok değişkenli varyans analizi (MANOVA) yapıldı. TCI'nın 7 ölçeği; Yenilik Arayışı (YA), Zarardan Kaçınma (ZK), Ödül Bağımlılığı (ÖB), Sebat Etme (SE), Kendini Yönetme (KY), İş Birliği Yapma (İY), Kendini Aşma (KA) bağımlı değişkendi. Yenilik Arayışı (YA) bağımlı değişkeninde, iki yönlü varyans analizi anlamlı "grup" ana etkisi ve "cinsiyet" ana etkisi göstermedi. Anlamlı "grup*cinsiyet" ortak etkisi elde edildi (F değerleri, serbestlik dereceleri ve olasılıklar için Tablo 6'ya bakınız). Ortak etki açısından, erkek sağlıklı gönüllülerde (ort. = 17.37) kadın sağlıklı gönüllülere (ort. = 18.96) kıyasla, yenilik arayışı özellikleri anlamlı olarak daha azdır.

Zarardan Kaçınma (ZK) bağımlı değişkeninde, iki yönlü varyans analizi anlamlı "grup" ve "cinsiyet" ana etkisi ve "grup*cinsiyet" ortak etkisi gösterdi (bakınız Tablo 6). Yani, psikiyatri hastalarında (ort. = 21.82), normallere (ort. = 16.39) kıyasla, zarardan kaçınma anlamlı olarak daha fazladır. Yine, kadınlar (ort. = 18.49), erkeklere (ort. = 17.94) göre daha fazla zarardan kaçınma içinde-

TABLO 7. TCI Altölçeklerinin Diskriminant İşlev Analizi Sonuçları.

	Diskriminant İşlevli Korelasyon Katsayıları	Diskriminant İşlevi İçin Standardize Katsayılar	
Yordayıcı Değişken	İşlev 1	İşlev 1	Univariate F (1,1012)
YA1	-.26	.10	23.95**
YA2	.17	.16	9.79*
YA3	.01	.02	0.05
YA4	-.05	-.00	0.72
ZK1	.64	.34	143.04**
ZK2	.42	.12	61.84**
ZK3	.36	-.08	46.58**
ZK4	.74	.57	192.86**
ÖB1	.05	.01	0.81
ÖB3	-.15	-.09	8.29*
ÖB4	.10	.07	3.30
SE	-.19	.07	12.21**
KY1	-.35	-.05	43.17**
KY2	-.33	.02	37.06**
KY3	-.44	-.08	69.38**
KY4	.09	.27	3.09
KY5	-.43	-.24	65.99**
İY1	-.21	-.07	15.94**
İY2	-.32	-.21	36.08**
İY3	-.06	.16	1.21
İY4	-.09	-.09	3.02
İY5	.06	.14	1.19
KA1	-.11	-.22	4.29
KA2	-.00	.35	0.00
KA3	-.17	-.25	9.86*
Canonical R	.51		
Eigenvalue	.35		

YA: Yenilik Arama, ZK: Zarardan Kaçınma, ÖB: Ödül Bağımlılığı, SE: Sebat Etme, KY: Kendini Yönetme, İY: İşbirliği Yapma, KA: Kendini Aşma.
**p < 0.001, *p < 0.01.

dirler. Ortak etki açısından, kadın psikiyatri hastalarında (ort. = 22.43) erkek psikiyatri hastalarına (ort. = 20.56) kıyasla, zarardan kaçınma anlamlı olarak daha fazladır.

Ödül bağımlılığı (ÖB) bağımlı değişkeninde, iki yönlü varyans analizi anlamlı “cinsiyet” ana etkisi gösterdi (bakınız Tablo 6). Anlamlı “grup” ana etkisi ve ortak etki bu değişkende gösterilemedi. Yani, kadınlar (ort. = 14.31), erkeklere (ort. = 12.99) göre daha fazla ödül bağımlılığı göstermektedir. Normal veya psikiyatri hastası olmanın bu bağımlı değişken üzerine anlamlı etkisi yoktu.

Sebat Etme (SE) bağımlı değişkeninde, iki yönlü varyans analizi anlamlı “grup” ana etkisi gös-

terdi. Anlamlı "cinsiyet" ana etkisi ve ortak etki bu değişkende gösterilemedi. Yani, psikiyatri hastaları (ort. = 4.73), normallere (ort. = 5.15) göre, anlamlı olarak daha az sebat etmektedirler.

Kendini Yönetme (KY) bağımlı değişkeninde, iki yönlü varyans analizi anlamlı "grup" ana etkisi gösterdi (bakınız Tablo 6). Anlamlı "cinsiyet" ana etkisi ve ortak etki bu değişkende gösterilemedi. Yani, psikiyatri hastalarında (ort. = 25.08), normallere (ort. = 28.15) kıyasla, kendini yönetme anlamlı olarak daha azdır. Cinsiyetin kendini yönetme üzerine anlamlı etkisi olmadığı görülmüştür.

İş Birliği Yapma (İY) bağımlı değişkeninde, iki yönlü varyans analizi anlamlı "grup" ana etkisi ve "cinsiyet" ana etkisi gösterdi (bakınız Tablo 6). Ortak etki gözlenmedi. Yani, psikiyatri hastalarının (ort. = 26.64), normallere (ort. = 28.40) göre, anlamlı olarak daha az, kadınların (ort. = 27.62), erkeklere (ort. = 26.38) göre daha fazla işbirliğine yatkın oldukları bulunmuştur.

Kendini Aşma (KA) bağımlı değişkeninde, iki yönlü varyans analizi anlamlı "grup" ana etkisi gösterdi (Bakınız Tablo 6). Anlamlı "cinsiyet" ana etkisi ve ortak etki bu değişkende gösterilemedi. Yani, psikiyatri hastalarında (ort. = 17.21), normallere (ort. = 18.09) kıyasla, kendini aşma anlamlı olarak daha azdır. Cinsiyetin kendini aşma üzerine anlamlı etkisi olmadığı görülmüştür.

Diskriminant Analizi

Psikiyatri hastası ve sağlıklı normal gruplarına ait olmanın yordayıcıları olarak, TCI'in 25 alt ölçeği kullanılarak diskriminant analizi gerçekleştirildi.

Tek diskriminant işlev hesaplandı, $\Lambda=0.74$, $\chi^2(25, N=1014)=29.73$, $p < 0.001$. Bu anlamlı Wilks' Lambda sonucu, yordayıcıların iki grubu anlamlı bir şekilde ayrıştırabildiğini göstermektedir.

Tablo 7'de diskriminant işlevli korelasyon katsayıları ve diskriminant işlevi için standart katsayılar verilmiştir. Bu katsayılara dayanarak, diskriminant işlevi ile en kuvvetli ilişkiyi Çabuk yorulma (ZK4) göstermektedir. Güçlü ilişki gösteren diğer yordayıcılar, Beklenti endişesi (ZK1), Uyumlu ikincil huylar (KY5) ve Empati duyma (İY2)'dır. Psikiyatri hastaları (ort.= 5.32), sağlıklı gönüllülere (ort.= 3.41) kıyasla, daha kolaylıkla yorulmaktadırlar. Psikiyatri hastaları (ort.= 7.29), sağlıklı gönüllülerden (ort.= 5.57) daha yoğun bir endişe ve kötümserlik içindedirler. Psikiyatri hastalarının (ort.= 7.86), sağlıklı gönüllülere (ortalama= 8.95) göre daha fazla uygunsuz alışkanlıklarının ve kişisel güvensizliklerinin olduğu söylenebilir. Psikiyatri hastalarının (ortalama=3.69), sağlıklı gönüllülere (ortalama= 4.24) göre daha sınırlı empati yeteneğine sahip oldukları yine söylenebilir.

Toplam 1014 kişiden oluşan örneklemin, grup üyeliği için sınıflandırma işleminin kullanımı ile, 735 kişi (%72.5) doğru bir şekilde sınıflandırılmıştır. 544 psikiyatri hastasının 397'si hasta olarak, 470 sağlıklı gönüllünün 338'i sağlıklı olarak sınıflandırılmıştır. Sınıflandırmanın duyarlılığı (sensitivity), %72; özgüllüğü (specificity), %73; olumlu yordayıcı değeri (positive predictive value), %70 ve olumsuz yordayıcı değeri (negative predictive value), %75'dir.

TARTIŞMA

Bu çalışmanın temel amacı, Mizaç ve Karakter Envanteri'nin Türkiye'de kullanılabilirliğini değerlendirmekti. Mizaç ve Karakter Envanteri'nin Türkçe versiyonunun psikometrik özellikleri, Amerika Birleşik Devletlerinde geliştirilen orijinal versiyonun ve çeşitli ülkelerde çalışılan versiyonların psikometrik özelliklerine benzerdir.

TCI'nin Türkçe versiyonunda ölçekler, düşük değerler alan Ödül Bağımlılığı ve Sebat etme hariç, kabul edilebilir düzeyde iç tutarlılık göstermiştir. İç tutarlılık güvenilirliği olarak, TCI ölçeklerinin Cronbach alfa değerleri, mizaç ölçeklerinde 0.55 ile 0.84, ve karakter ölçeklerinde 0.80 ile 0.84 arasındadır. En düşük Cronbach alfa katsayıları Ödül Bağımlılığı (0.55) ve Sebat etme'de (0.56) bulunmuştur. Sebat etme, önceleri ödül bağımlılığının altölçeklerinden biri olarak düşünülmüştür, ancak, ödül bağımlılığının diğer altölçekleriyle korelasyon göstermediği görüldüğü için, ölçek gözden geçirilmiş ve dördüncü mizaç faktörü olarak eklenmiştir. Ancak, ülkemizde Köse ve arkadaşları (2004) tarafından yapılan çalışmada ve diğer ülkelerde yapılan bir çok çalışmada (İsveççe versiyonu, Brandstrom ve ark., 1998; Almanca versiyonu, Richter ve ark., 1999; Hollandaca versiyonu, Duijsens ve ark., 2000; Fransızca versiyonu, Pelissolo ve Lepine, 2000; İspanyolca versiyonu, Gutierrez ve ark., 2001; Korece versiyonu, Sung ve ark., 2002), bağımsız bir değişken olarak yeterli psikometrik özellikleri göstermemiş ve iç tutarlılığı genel olarak düşük çıkmıştır. Ödül Bağımlılığı

ve Sebat etme'ye ilişkin güçlükler, kısmen, bu ölçeklerin madde sayılarının az oluşuna (sebat etme ölçeğinin yalnızca 8 maddesi vardır), kısmen de, orjinal TCI'den çeviri sorunlarına bağlı olabilir. TCI altölçeklerinin Cronbach alfa değerleri, genel olarak, İş Birliği Yapma ölçeği hariç, her bir ölçek ile tutarlıdır. İş Birliği Yapma (İY) ölçeğinin Cronbach alfa değeri 0.80 iken, altölçekleri çok geniş bir çeşitlilik göstermiştir (0.35 ile 0.79 arasında). Empati duyma (İY2, α =0.36) altölçeğinde "Keşke başkaları bu kadar çok konuşmasalar." ve Yardımseverlik (İY3, α =0.35) altölçeğinde "Bir ekibin üyeleri, paylarını nadiren dürüstçe alırlar." maddeleri altölçekleriyle zayıf iç tutarlılık göstermiştir. Zarardan Kaçınma'nın altölçekleri, nisbeten durağan (0.64 ile 0.71 arasında değişen) Cronbach alfa değerleri gösterdiler. Kendini Yönetme (KY) altölçekleri, 0.51 olan KY3 altölçeği hariç, 0.57 ile 0.69 değerleri arasındadır. Kendini Aşma altölçeklerinin Cronbach alfa değerleri 0.63 ile 0.70 arasındadır. Yenilik Arayışı altölçekleri de, İş Birliği Yapma altölçeklerine benzer bir şekilde geniş bir çeşitlilik göstermiştir (0.31 ile 0.67). Cronbach alfa değeri 0.31 olan Düzensizlik (YA4) altölçeğinde, "Her ne zaman olursa olsun, çok düzenli olmak ve kişiler için kurallar koymaktan hoşlanırım." maddesinin çıkarılması Cronbach alfa değerini 0.34'e çıkarmaktadır. Özetle, 24 altölçekten 11'i 0.60 değerinin altındadır. Bu iç tutarlılığı oldukça düşük ve zayıf olan altölçekler, tek başlarına kullanılmadıkları sürece, önemli bir sorun teşkil etmeyebilir. Klinik ve epidemiyolojik çalışmalardan elde edilecek bütün önemli çıkarsamaların, ağırlıklı olarak ölçeklere dayanacağını söylemek mümkün görünmektedir.

Çalışmanın temel amaçlarından biri, çeşitli kültürlerde bulunmuş olan kişilik boyutlarının kültürümüze genellenebilirliğini görmektir. Araştırma sonuçları TCI tarafından ölçülen kişilik boyutlarının genellenebilirliğini desteklemiştir. Dünyanın çok çeşitli yerlerinde yapılan çalışmalarda bulunan kişilik faktörleri genel olarak ülkemizde de bulunmuştur. Ancak, TCI'nin 25 altölçeğinin birlikte analize girdiği, "oblimin" dönüştürmesine göre yapılan Temel Bileşenler faktör analizinin, öngörülen TCI faktör yapısını tekrar ettiğini söylemek güçtür. Yedi-faktörlü çözüme göre ortaya çıkan örüntü basit değildir. Yalnızca Yenilik Arayışı, Zarardan Kaçınma ve Kendini Aşma bir faktöre yük vermiştir. Sebat etme ölçeği, en azından bu örneklemde, kesin olarak kişilik modelinin ayrı bir boyutu değildir. Mizaç ve karakter boyutları aynı faktörlerde binişmektedir. Kendini Yönetme ve İş Birliği Yapma tam olarak tanımlanamamıştır. Cloninger'in psikobiyolojik kuramı için yedi-faktörlü çözümü kullanan çalışmalar karışık sonuçlar bildirmektedir. Cloninger'in Amerika'da yapmış olduğu çalışma (1993) ve Hollanda'da yapılan çalışma (Duijsens ve ark., 2000) mizaç ve karakter boyutlarını birleşik olarak başarılı bir şekilde yedi faktörlü çözümü ortaya koymuştur. İsveççe versiyonu (Brandstrom ve ark., 1998), Almanca versiyonu (Richter ve ark., 1999), Fransızca versiyonu (Pelissolo ve Lepine, 2000), İspanyolca versiyonu (Gutierrez ve ark., 2001), Korece versiyonu (Sung ve ark., 2002) ve Japonca versiyonu (Kijima ve ark., 1996) kullanan çalışmalar ve yine Amerika'da yapılmış, Cloninger'in çalışmasının örneklemine göre daha geniş ve daha yaşlı bir örneklemde yapılmış bir çalışma, bir çözümde yedi faktörü çıkarmakta güçlük çektikleri için, birleşik bir yedi faktörlü çözüm yerine, mizaç ve karakterin faktör analizi sonuçlarını ayrı ayrı olarak bildirmişlerdir. Bu çalışmada da, yedi-faktörlü çözüme göre ortaya çıkan faktör yapısı Cloninger'in kişilik modeli ile tam bir tutarlılık göstermediği için, verilere, mizaç ve karakter altölçekleri için ayrı ayrı olarak, faktör analizi tekrarlanmıştır. Genel olarak, hem mizaç hem de karakter boyutlarında, özgül bir boyutla ilişkili olan altölçekler en yüksek faktör yüklerini tek bir boyuta vermişlerdir.

Mizaçlarda, üç faktörlü çözüme göre, Zarardan Kaçınma ve Ödül Bağımlılığı oldukça kuvvetli gözükmüştür. Yenilik Arayışı faktöründe, Dürtüsellik (YA2), Savurganlık (YA3), Düzensizlik (YA4) altölçekleri tutarlı bir şekilde yük verirken, Keşfetmekten heyecan duyma (YA1) altölçeği yalnızca çok zayıf bir yük sağladı. Keşfetmekten heyecan duyma altölçeği (YA1), daha çok, negatif olarak Zarardan Kaçınma'ya ve pozitif olarak Ödül Bağımlılığı'na yük verdi. Sebat etme en yüksek faktör yükünü negatif olarak Yenilik Arayışı'na verdi. Üç faktör, toplam varyansın %24, %15 ve %11'ini (kümülatif olarak %50'sini) açıkladılar. Ancak, bu üç faktör tarafından açıklanan varyans, daha önceki çalışmalara göre azdır. Benzer analizlerde, mizaç faktörleri tarafından açıklanan varyans, Brandstrom ve arkadaşları (1998) tarafından, %58.9, Richter ve arkadaşları (1999) tarafından, %62, Gutierrez ve arkadaşları (2001) tarafından, %60.3 ve Sung ve arkadaşları (2002) tarafından, %61.2 olarak bulunmuştur.

Mizaç boyutlarında, Keşfetmekten heyecan duyma altölçeği (YA1), Yenilik Arayışı'ndan daha

çok, Zarardan Kaçınma'ya (negatif olarak) yük verir görünmektedir. Benzer sonuçlar, Türkiye'deki çalışmada (Köse ve ark., 2004) ve Almanca versiyonu (Richter ve ark., 1999), Fransızca versiyonu (Pelissolo ve Lepine, 2000) ve Belçika dili versiyonunu (Hansenne ve ark., 2001) kullanan çalışmalarda da elde edilmiştir. Bu sonuçlar, araştırıcı eğilimlerin ketleyici özelliklerden önemli miktarda etkilendiğini ve Keşfetmekten heyecan duyma altölçeği (NS1)'in heterojen bir alt faktör olduğunu göstermektedir. Bu çalışmanın bir diğer beklenmedik bulgusu, Sebat etme ölçeğinin kişilik modelinin ayrı, izole bir boyutu olmadığı ve en yüksek faktör yükünü negatif olarak Yenilik Arayışı'na verdiğidir. Bir çok çalışma, Israrcılık'ın bağımsız bir değişken olarak yeterli psikometrik özellikleri göstermediğini bildirmektedir.

Karakterlerde, üç faktörlü çözüme göre, İş Birliği Yapma ve Kendini Aşma oldukça sağlam gözükmüştür. Kendini Yönetme faktöründe, Sorumluluk alma (KY1), Amaçlılık (KY2), Beceriklilik (SD3) ve Uyumlu ikincil huylar (KY5) altölçekleri tutarlı bir şekilde yük verirken, Kendini kabullenme (KY4) altölçeği yük vermemiştir. Kendini kabullenme (KY4), beklenmedik bir şekilde, daha çok, pozitif olarak İş Birliği Yapma'ya ve negatif olarak Kendini Aşma'ya yük vermiştir. Üç faktör, toplam varyansın %27, %16 ve %10'nunu (kümülatif olarak %53'ünü) açıkladılar. Benzer sonuç, Köse ve arkadaşları (2004) ve Amerikan örnekleminde, Svrakic ve arkadaşları (1993) tarafından da bildirilmiştir. Cloninger, bunu, kişinin kendi sınırlarını kabul etme yeteneğinin, diğer insanların sınırlarını kabul etme ve bunlara hoşgörü gösterme yeteneği ile bağlantılı olduğu şeklinde yorumlamaktadır.

TCI mizaç ve karakter ölçeklerinin korelasyon örüntülerine bakıldığında, Kendini Yönetme, Zarardan Kaçınma ile negatif yönde anlamlı ilişki ($r = -0.49$) göstermiştir. İş Birliği Yapma, hem Ödül Bağımlılığı ($r = 0.41$) hem de Kendini Yönetme ($r = 0.45$) ile pozitif yönde anlamlı ilişki göstermiştir. Kendini yönetmenin düşük oluşu, zarardan kaçınmanın yüksek oluşunu beraberinde getirmektedir. Bu ilişki, anksiyeteli kişilerin hedefler ve kişisel değerler seçme ve kendilerini oldukları gibi kabul etme konusunda güçlük çektikleri anlamına gelmektedir. İşbirliğine yatkınlığın yüksek oluşu ise, hem ödül bağımlılığının hem de kendini yönetmenin yüksek oluşunu beraberinde getirmektedir. Bu korelasyonlar, bu boyutların kişilerarası ilişkilerle bağlantılı olmalarından kaynaklanmaktadır. Benzer korelasyon örüntüsü, Amerikan (Cloninger ve ark., 1993), İsveç (Brandstrom ve ark., 1998), Alman (Richter ve ark., 1999), Fransız (Pelissolo ve Lepine, 2000), İspanyol (Gutierrez ve ark., 2001), Kore (Sung ve ark., 2002), Çek (Kozeny ve Höschl, 1999), Hollanda (Duijsens ve ark., 2000) ve Japon (Kijima ve ark., 1996) örneklemlerinde de elde edilmiştir (çalışmalarda Kendini Yönetme ile Zarardan Kaçınma'nın korelasyon katsayıları aralığı -0.39 ila -0.47, bu çalışmada -0.49; İş Birliği Yapma ile Ödül Bağımlılığı'nın korelasyon katsayıları aralığı 0.36 ila 0.57, bu çalışmada 0.41; İş Birliği Yapma ile Kendini Yönetme'nin korelasyon katsayıları aralığı 0.38 ila 0.57, bu çalışmada 0.45).

Psikobiyolojik kurama göre, mizaç boyutları erken çocukluk döneminde gözlenebilmektedir. Çünkü, bunlar kalıtsaldır ve yaşamın erken döneminde ortaya çıkarlar. Karakter boyutları ise yetişkinlikte olgunlaşır. Yaş ile TCI karakter boyutları arasındaki bağlantıyı analiz eden kesitsel bir çalışma (Cloninger ve ark., 1993) yukarıdaki varsayımı kısmen desteklemiştir. Yaş ile Kendini Yönetme ve İş Birliği Yapma arasında orta düzeyde pozitif korelasyon bulunmuş olması, karakterin yaşla gelişim gösterdiğini düşündürtmektedir. TCI mizaç ölçekleri ile yaş arasındaki ilişkiye dair veriler, Yenilik Arayışı puanlarının özellikle gençlerde daha yüksek olduğunu belirtmektedir. (Otter ve ark., 1995; Weyers ve ark., 1995). Bu bulgular, mizaç boyutlarının tamamının erken çocukluk döneminde ortaya çıkmadıklarını ve yaşam boyu durağan kalmadıklarını göstermektedir. Bu çalışmada, yaş, Yenilik Arama ($r = -0.24$) ile anlamlı seviyede negatif ve Kendini Yönetme ($r = 0.11$) pozitif korelasyon gösterdi. Yaş ile Yenilik Arayışı arasındaki ters yöndeki ilişki ilk olarak Cloninger (1986) tarafından ortaya atılmıştır. Yeni uyaranlara ilginin azalması, olgunluğun ve günlük sosyal ilişkilere katılımın artmasına eşlik etmektedir. Yenilik Arayışı puanları, yaklaşık olarak her on yılda bir puan azalmaktadır (Cloninger ve ark., 1991). Yaş ile Yenilik Arayışı arasındaki negatif ilişki bir çok çalışmada (Pelissolo ve Lepine, 2000; Brandstrom ve ark., 2001; Duijsens ve ark., 2000; Hansenne ve ark., 2001; Mendlowicz ve ark., 2000; Köse ve ark., 2004) bulunmuştur. Yaş'ın yüksek oluşu, beraberinde kendini yönetmenin yüksek oluşunu getirmektedir. Cloninger'in görüşlerine göre, yaşam boyunca kişilerin olgunlaşması, yaş ile Kendini Yönetme arasındaki ilişkinin önemi-

ni etkilemektedir. Bu çalışmanın bulguları, genel olarak, kişilik boyutlarının göreceli durağanlığını doğrulamaktadır. McCrae ve Costa (1988, aktaran, Somer, 1998), uzunlamasına olarak sürdürülen bir çok boylamsal çalışmanın kişilik özelliklerindeki ortalama düzeyin, yetişkinlikte 40 yıl gibi sürelerle tutarlılığını sürdürdüğüne ilişkin verileri bildiren çalışmalar bulunduğunu belirtmektedir. Araştırmacılar, kişilik özelliklerinin tutarlılığına ilişkin olarak düzenledikleri 6 yıllık boylamsal bir çalışma sonucunda, çeşitli kişilik boyutlarında oldukça yüksek test-tekrar test korelasyonları bulmuşlardır. Yazarlar, farklı boylamsal çalışmaların yaşlanmanın kendisinin kişilik üzerinde çok az bir etkiye sahip olduğunu ortaya koyduklarını ve bunun da yaşlanma süreci içerisinde hastalıklar, boşanmalar, işsizlik gibi çeşitli olumsuz olayların yaşanıyor olmasına rağmen elde edilmiş bir sonuç olduğuna dikkat çekmektedirler. Ayrıca, normal populasyonda çok büyük bir değişiklik gözlenmezken, kişilikteki çarpıcı değişikliklerin daha çok psikiyatrik veya nörofizyolojik bozuklukları olan kişilerde ortaya çıkabileceğini öne sürmektedirler.

Psikiyatri hastaları ile normalleri karşılaştırmak için yapılan varyans analizleri, Zarardan Kaçınma, Sebat etme, Kendini Yönetme, İş Birliği Yapma ve Kendini Aşma değişkenlerinde anlamlı farklılık verdi. Psikiyatri hastalarında, normallere kıyasla, tedbirlilik, utangaçlık, şüphecilik ve kötümserlik anlamlı olarak daha fazla ve azimlilik, sebatkarlık, sorumluluk, amaca yönelik olma, beceriklilik, sosyal kabul, empati, merhametli olma ve manevi kabullenme daha azdır. Bu bulgular, daha önce çeşitli kültürlerde yapılmış olan çalışmaların bulguları ile tutarlılık göstermektedir. Mizaç boyutu "Zarardan Kaçınma (ZK)" ve karakter boyutu "Kendini Yönetme (KY)", depresyon ve depresif duygulanım ile psikiyatri hastalarında (Richter ve ark., 2000; Svrakic ve ark., 1992) ve klinik olmayan örneklemlerde (Peirson ve Heuchert, 2001; Naito ve ark., 2000) tutarlı bir şekilde ilişkili bulunmaktadır. "Zarardan Kaçınma (ZK)"'dan yüksek puan ve "Kendini Yönetme (KY)"'den düşük puan almak, çeşitli psikiyatrik bozuklukların genel bir karakteristiği olarak kabul edilmektedir (Bulik ve ark., 1995). Afektif bozukluğu olan hastalar, anlamlı olarak yüksek "Zarardan Kaçınma (HA)", düşük "Kendini Yönetme (KY) ve düşük "İş Birliği Yapma (İY)" göstermektedirler (Hansenne ve ark., 1999). Yüksek "Zarardan Kaçınma (HA)", düşük "Kendini Yönetme (KY) ve düşük "İş Birliğine Yapma (İY)", anksiyete bozuklukları (Ball ve ark., 2002), sosyal fobi (Pelissolo ve ark., 2002), panik bozukluk (Starcevic ve ark., 1996), obsesif-kompulsif bozukluk (Kusunoki ve ark., 2000), şizofreni (yukarıdakilere ilaveten "Yenilik Arama (YA)" düşük, (Guillem ve ark., 2002) alkol bağımlılığı (yukarıdakilere ilaveten "Yenilik Arama (YA)" yüksek, Basiaux ve ark., 2000) ve yeme bozuklukları (yukarıdakilere ilaveten, "Yenilik Arama (YA)", bulimia nervozada yüksek, anoreksiya nervozada düşük, Fassino ve ark., 2002; Fassino ve ark., 2002) olan hastalarında karakteristiği olarak bulunmuştur. Bu bulgular, TCI'nin yapı geçerliliğine katkı sağlamaktadırlar ve TCI'nin psikiyatrik ve normal örneklemlerinin araştırılmasında kullanılabileceğini göstermektedir.

Psikiyatri hastası ve sağlıklı normal gruplarına ait olmanın yordayıcıları olarak, TCI'in 25 alt ölçeği kullanılarak gerçekleştirilen diskriminant analizi sonuçları, yordayıcıların iki grubu anlamlı bir şekilde ayırdedebildiğini göstermektedir. Diskriminant işlevi ile en kuvvetli ilişkiyi Çabuk yorulma (ZK4) göstermiştir. Diğer güçlü ilişki gösteren yordayıcılar, Beklentisel endişe (ZK1), Ahenkli alışkanlıklar (KY5) ve Empati'dir (İY2). Psikiyatri hastaları, sağlıklı gönüllülere kıyasla, daha kolaylıkla yorulmaktadırlar, daha yoğun bir endişe ve kötümserlik içindedirler ve daha fazla uygunsuz alışkanlıklarının ve kişisel güvensizliklerinin olduğu söylenebilir. Bu bulgular, TCI'nin yapı geçerliliğine ek bir katkı sağlamaktadırlar.

Sonuçlar, cinsiyetin Mizaç ve Karakter Envanterinin Ödül Bağımlılığı, İş Birliği Yapma ve Zarardan Kaçınma boyutlarının üzerine etkisi olduğunu da gösterdi. Kadınlar, erkeklere göre, kendilerine daha fazla sempatik, müşfik, anlayışlı, daha fazla empatik, merhametli, hoşgörülü, destekleyici ve daha fazla tedbirli, güvensiz, kötümser olmayı atfetmektedirler. Kadınlarda, yüksek ZK, ÖB ve İY, TCI kullanan birçok diğer çalışmada da bulunmuştur (Hansenne ve ark., 2001; Pelissolo ve Lepine, 2000; Brandstrom ve ark., 1998; Duijsens ve ark., 2000; Mendlowicz ve ark., 2000). Hansenne ve arkadaşları (2001), bu çalışmada da bulunduğu gibi, kadınlarda düşük KY puanları elde ederken, Pelissolo ve Lepine (2000) yüksek KY puanları bulmuşlardır. Bu cinsiyete özgü farklılıkların nedenlerine ilişkin çok çeşitli görüşler öne sürmek mümkündür. Bu farklılıklar, bir açıdan, ZK ve ÖB için, genetik olarak belirlenmiş etkileri yansıtabilir. Buss (1999, aktaran, Mendlowicz ve ark., 2000), kalıtsal kişilik özelliklerindeki bireysel farklılıkla-

rın, hayatta kalma ve cinsellik gibi üreme başarısı ile yakından ilişkili olan aktivitelerle bağlantılı olduğunu belirtmiştir. Cinsiyete özgü farklılıklara dair bilimsel bulguları gözden geçirdikten sonra, Buss, kadınların, erkeklere göre daha etkin ebeveynlik yapmalarını sağlayacak ilgi ve duygusal akıl okuma mekanizmaları ile evrimleştiklerini iddia etmektedir. Ödül bağımlılığı (ÖB) yüksek kişilerin duygusal, sosyal olarak duyarlı, şefkatli ve fedakar kişiler oldukları dikkate alınırsa, ödül bağımlılığının yüksek oluşunun kadınların ebeveynlik etkinliğini arttıran mekanizmalardan biri olabileceği düşünülebilir.

Sonuç olarak, çalışmacılar, Köse ve arkadaşlarının (2004) yaptıkları çalışmanın bulgularını da dikkate alarak, TCI'nin Türkçe versiyonunun psikometrik özelliklerinin (faktör yapısı, iç tutarlılık, geçerlilik) tatmin edici olduğunu ve ülkemizde kullanılabileceğini düşünmektedir. TCI, hem klinik uygulamalarda hem de araştırmalarda kişiliği değerlendirmek için kullanılabilecek yararlı bir araç olarak görünmektedir.

KAYNAKLAR

Ball S, Smolin J, Shekhar A (2002) A psychobiological approach to personality: examination within anxious outpatients. J Psychiatr Res, 36: 97-103.

Basiaux P, Le Bon O, Dramaix M ve ark. (2001) Temperament and character inventory (TCI) personality profile and sub-typing in alcoholic patients: a controlled study. Alcohol Alcohol, 36: 584-587.

Branstrom S, Schlette P, Pryzbeck TR ve ark. (1998) Swedish normative data on personality using the Temperament and Character Inventory (TCI). Compr Psychiatry, 39: 122-128.

Bulik CM, Sullivan PF, Joyce PR ve ark. (1995) Temperament, character, and personality disorder in bulimia nervosa. J Nerv Mental Disease, 183: 593-598.

Cloninger CR (1986) A unified biosocial theory of personality and its role in the development of anxiety states. Psychiatr Dev, 3: 167-226.

Cloninger CR (1987) A systematic method for clinical description and classification of personality variants. Arch Gen Psychiatry, 44, 573-588.

Cloninger CR, Przybeck TR, Svrakic DM (1991) The Tridimensional Personality Questionnaire: US normative data. Psychol Rep, 69: 1047-1057.

Cloninger CR, Svrakic DM (2000) Personality disorders. In B. J. Sadock and V. A. Sadock (Eds.), Comprehensive Textbook of Psychiatry, 7th. edn., Baltimore: Williams &Williams.

Cloninger CR, Svrakic DM, Przybeck TR (1993) A psychobiological model of temperament and character. Arch Gen Psychiatry, 50: 975-990.

Cloninger CR, Przybeck TR, Svrakic DM, Wetzel RD (1994) The Temperament and Character Inventory (TCI): a guide to its development and use. St Louis, Missouri, USA: Center for Psychobiology of Personality.

Duijsens IJ, Spinhoven P, Goekoop JG ve ark. (2000) The Dutch temperament and character inventory (TCI): dimensional structure, reliability and validity in a normal and psychiatric outpatient sample. Personality and Individual Differences, 28: 487-499.

Fassino S, Abbate-Daga G, Amianto F ve ark. (2002) Temperament and character profile of eating disorders: a controlled study with the temperament and character inventory. Int J Eat Disord, 32: 412-425.

Fassino S, Svrakic D, Abbate-Daga G ve ark. (2002) Anorectic family dynamics: temperament and character data. Compr Psychiatry, 43: 114-120.

Guillem F, Bicu M, Semkovska M ve ark. (2002) The dimensional symptom structure of schizophrenia and its association with temperament and character. Schizophr Res, 56: 137-147.

Gutierrez F, Torrens M, Boget T ve ark. (2001) Psychometric properties of the temperament and character inventory (TCI) questionnaire in a Spanish psychiatric population. Acta Psychiatr Scand, 103: 143-147.

Hansenne M, Le Bon O, Gauthier A ve ark. (2001) Belgian normative data of the temperament and character inventory. Eur J of Psychol Assess, 17: 56-62.

Kijima N, Saito R, Takeuchi M ve ark. (1996) Cloninger's seven-factor model of temperament and character and Japanese version of Temperament and Character Inventory (TCI). Jpn J Psychiatr Diagnoses, 7: 379-399.

Kozeny J, Höschl C (1999) The temperament and character inventory: psychometric integrity of the Czech version. Studia Psychologica, 41: 123-132.

Köse S, Sayar K, Ak İ ve ark. (2004) Mizaç ve Karakter Envanteri (Türkçe TCI): Geçerlik, güvenirliği ve faktör yapısı. Klinik Psikofarmakoloji Bülteni, 14: 107-131.

Kusunoki K, Sato T, Taga C ve ark. (2000) Low novelty-seeking differentiates obsessive-compulsive disorder from major depression. Acta Psychiatr Scand, 101: 403-405.

Mendlowicz MV, Jean-Louis G, Gillin JC ve ark. (2000) Sociodemographic predictors of temperament and character. J Psychiatr Res, 34: 221-226.

Naito M, Kijima N, Kitamura T (2000) Temperament and character inventory (TCI) as predictors of depression among Japanese college students. J Clin Psychol, 56: 1579-1585.

Otter C, Huber J, Bonner A (1995) Cloninger's Tridimensional Personality Questinnaire: reliability in an English sample. Personality and Individual Differences, 18: 471-480.

Peirson AR, Heuchert JW (2001) The relationship between personality and mood: comparison of the BDI and the TCI. Personality and Individual Differences, 30: 391-399.

Pelissolo A, Lepine JP (2000) Normative data and factor structure of the Temperament and Character Inventory (TCI) in the French version. Psychiatry Res, 94: 67-76.

Pelissolo A, Andre C, Pujol H ve ark. (2002) Personality dimensions in social phobics with or without depression. Acta Psychiatr Scand, 105: 94-103.

Richter J, Brandström S, Przybeck T (1999) Assessing personality: the temperament and character inventory in a cross-cultural comparison between Germany, Sweden, and the USA. Psychol Rep, 84: 1315-1330.

Richter J, Eisemann M, Richter G (2000) Temperament and character during the course of unipolar depression among inpatients. Eur Arch Psychiatry Clin Neurosci, 250: 40-47.

Somer O (1998) Beş-faktör kişilik modeli. Türk Psikoloji Yazıları, 1: 35-62.

Starcevic V, Uhlenhuth EH, Fallon S ve ark. (1996) Personality dimensions in panic disorder and generalized anxiety disorder. J Affect Disord, 37: 75-79.

Sung SM, Kim JH, Yang E ve ark. (2002) Reliability and validity of the Korean version of the temperament and character inventory. Compr Psychiatry, 43: 235-243.

Svrakic DM, Przybeck TR, Cloninger CR (1992) Mood states and personality traits. J Affect Disord, 24: 217-220.

Svrakic DM, Whitehead C, Przybeck TR ve ark. (1993) Differential diagnosis of personality disorders by the seven factor model of temperament and character. Arch Gen Psychiatry, 50: 991-999.

Tanaka E, Kijima N, Kitamura T (1997) Correlations between the the temperament and character inventory and the self-rating depression scale among Japanese students. Psychol Rep, 80: 251-254.

Waller N, Lillienfeld SO, Tellegen A ve ark. (1991) The Tridimensional Personality Questionnaire: structural validity and comparison with the Multidimensional Personality Questionnaire. Multivariate Behav Res, 26: 1-23.

Weyers P, Krebs H, Janke W (1995) Reliability and construct validity of the German version of Cloninger's Tridimensional Personality Questionnaire. Personality and Individual Differences, 19: 853-861.

RASİM ADASAL RUH SAĞLIĞI BİLİM ÖDÜLÜ

1. **Rasim Adasal Ruh Sağlığı Bilim Ödülü** kurucusu olduğu *Ankara Üniversitesi Tıp Fakültesi Psikiyatri Anabilim Dalı* ve *Türkiye Sosyal Psikiyatri Derneği* işbirliğiyle düzenlenmiştir. 1999 yılından beri verilmektedir.
2. Ödül, modern psikiyatriyi Türkiye'de yerleştiren ve kamuoyunda yaptığı çalışmalarla toplumla psikiyatriyi bütünleştiren **Prof. Dr. Rasim ADASAL**'ın adına konulmuştur. Onun hizmetlerinin gelecek kuşaklara aktarılması ve tanıtılması amacını taşımaktadır. Böylelikle ruh hekimi ve topluma mal olmuş bir kişilik olarak onun anısını canlı tutmak amaçlanmıştır.
3. Ödül, verildiği yıl esas olmak üzere, son beş yıl içinde ruh sağlığı ve hastalıkları alanında ülkemizde yapılmış ve yurt dışında yayınlanmış klinik çalışmalara verilmektedir.
4. Ödül jürisi her yıl Ankara Tıp Fakültesi Psikiyatri Anabilim Dalı tarafından belirlenecek yedi kişiden oluşmaktadır. Ödül jürisi seçim sonucunu o yıl yapılan Ulusal Psikiyatri Kongresi'nde ilan edecektir.
5. Ödül için başvuru her yıl 01 Ocak-31 Temmuz tarihleri arasındadır.
6. Ödüle başvuran çalışmalar için daha önce ödül almamış olma koşulu vardır.
7. Ödül başvurusunda bulunan araştırmacının çalışmadaki ilk iki isimden biri olması ve özgeçmişi ile yayın listesini başvurusuna eklemesi gerekmektedir.
8. Başvuruların değerlendirilmesinden gözönüne alınacak temel ölçütler şunlardır:

   i. Özgünlük
   ii. Yöntemsel yetkinlik
   iii. Ülkemiz bilim aktivitesini temsil niteliği
   iv. Yayımlandığı dergi
9. Ödül jürisinde görev alanlar her çalışma için gerekçeli kişisel rapor hazırlayacak ve yapacakları sıralama değerlendirmede esas alınacaktır.
10. Ödül tutarı her yıl için ayrıca belirlenecek olup Türkiye Sosyal Psikiyatri Derneği tarafından karşılanacaktır.
