# Supplementary material for: The Role of Willpower in Major Depressive Disorder: An fMRI Study
Source: Brain Behav. 2025 Oct 20;15(10):e70921. doi: 10.1002/brb3.70921 (PMC12537831; doi:10.1002/brb3.70921)
Supplement: Supplementary file 3 — Supplementary Materials: brb370921‐sup‐0003‐SuppMat.pdf [file BRB3-15-e70921-s001.pdf]

Mizaç ve Karakter Envanteri (Türkçe TCI): Geçerlik, Güvenirliği ve Faktör Yapısı

Samet Köse[1], Kemal Sayar[2], İsmail Ak[3], Nazan Aydın[4], Ülgen Kalelioğlu[5]
İsmet Kırpınar[6], Robert A. Reeves[7], Thomas R. Przybeck[8], C. Robert Cloninger[8]

ÖZET:

Mizaç ve karakter envanteri (Türkçe TCI): Geçerlik, güvenirliği ve faktör yapısı*

Giriş: Cloninger kişiliğin iki temel bileşeni olan mizaç ve karakterdeki normal ve anormal varyasyonları açıklayan boyutsal bir psikobiyolojik kişilik modeli geliştirmiştir. Mizaç boyutu Yenilik Arayışı (NS), Zarardan Kaçınma (HA), Ödül Bağımlılığı (RD) ve Sebat Etme (P); karakter boyutu ise, Kendini Yönetme (SD), İş Birliği Yapma (C) ve Kendini Aşma (ST) alt ölçeklerinden oluşur. Mizaç ve Karakter Envanteri (TCI) kişiliğin yedi temel boyutunu ölçmeye yarayan, 240 maddeden oluşan "Doğru" ya da "Yanlış" şeklinde yanıtlanan bir kendini değerlendirme ölçeğidir. TCI önceden geliştirilmiş kişilik modellerini kuramsal ve ampirik açıdan desteklerken, klinik kullanımdaki sınırlılıkları da ortadan kaldırmıştır. Bu çalışmada Köse ve Sayar tarafından çevrilen Türkçe TCI'nin sağlıklı bir Türk örnekleminde psikometrik özellikleri ve faktör yapısı araştırılmıştır.
Yöntem: Çalışmanın örneklemini Karadeniz Teknik Üniversitesi Tıp Fakültesi ile Atatürk Üniversitesi Tıp Fakültesinde, hayatlarının hiçbir doneminde ruhsal rahatsızlık nedeniyle tedavi görmemiş sağlıklı gönüllü 683 denek oluşturmuştur. Deneklere Marlowe-Crowne Sosyal Arzu Edilirlik Ölçeği Kısa Formu ve ardından Türkçe TCI verilmiştir.
Bulgular: Mizaç boyutunda, Türk toplumu ortalama Yenilik Arayışı, Ödül Bağımlılığı puanları Amerikan toplumu ortalamalarından anlamlı olarak düşük, Zarardan Kaçınma puanları Amerikan toplumu ortalama değerlerinden anlamlı olarak yüksek bulundu. Karakter boyutunda; Kendini Yönetme, İş Birliği Yapma, Kendini Aşma puanları Amerikan toplumu ortalama değerlerinden anlamlı olarak düşük bulundu. Kendini Yönetme ile Zarardan Kaçınma, İş Birliği Yapma ile Ödül Bağımlılığı ve İş Birliği Yapma ile Kendini Yönetme arasında anlamlı korelasyon bulundu. Türkçe TCI ölçeklerinin Cronbach alfa değerleri mizaç boyutunda .60 ile .85, karakter boyutunda .82 ile .83 arasında bulundu. En düşük Cronbach alfa katsayıları Ödül Bağımlılığı (.60) ve Sebat Etme (.62) olarak bulundu. Oblimin dönüştürümlü faktör analizinde, mizaç boyutunda Yenilik Arayışı ve Zarardan Kaçınma alt ölçeklerinin oldukça güçlü yüklemede bulundukları saptandı. Ödül Bağımlılığı ve Sebat Etmenin göreceli olarak daha zayıf yüklemede bulunduğu saptandı. Karakter boyutunda, İşbirliği Yapma ve Kendini Aşmanın oldukça tutarlı ve güçlü yüklemede bulundukları gözlendi.
Sonuç: Türkçe TCI'nin geçerlik ve güvenirliği, ölçek puanlarının dağılımı, yeterli düzeyde iç tutarlık katsayısı ve yapı geçerliği tarafından desteklenmiştir. Bu öncül çalışma Türkçe TCI'nin Türk toplumunda Cloninger'in yedi-faktörlü kişilik modelini başarıyla ölçebileceğini göstermektedir. Türkçe TCI psikiyatrik hasta popülasyonunda klinik uygulamalarda, nörobiyolojik çalışmalar ve beyin görüntüleme çalışmalarında kişiliği ölçmede yararlı bir araç olarak görünmektedir.

Anahtar sözcükler: kişilik, mizaç, karakter, TCI, kültürler arası

Klinik Psikofarmakoloji Bülteni 2004;14:107-131

ABSTRACT:

Turkish version of the Temperament and Character Inventory (TCI): Reliability, validity, and factorial structure

Objective: Cloninger has developed a dimensional psychobiological model of personality that accounts for both normal and abnormal variation of two major components of personality: temperament and character. The Temperament and Character Inventory (TCI) is a 240-item self-administered questionnaire constructed to assess four temperament (Novelty Seeking, Harm Avoidance, Reward Dependence, and Persistence) and three character dimensions (Self-Directedness, Cooperativeness, and Self-Transcendence). In this study, we aimed to examine the psychometric properties of the Turkish version of the TCI in a healthy Turkish population and obtain normative data for the Turkish TCI.
Methods: The study was conducted in both Karadeniz Technical University School of Medicine and Atatürk University School of Medicine using a sample of 683 healthy volunteers. Participants were administered a short version of Marlowe-Crowne Social Desirability Scale and the Turkish TCI that was translated by Kose and Sayar and officially approved by Cloninger to be used in this validation study.
Results: Turkish sample had significantly lower mean scores on Novelty Seeking, Reward Dependence and higher mean scores on Harm Avoidance than the American sample. On character dimensions, the Turkish sample had significantly lower scores on Self-Directedness, Cooperativeness, and Self-Transcendence. Self-Directedness and Harm Avoidance, Cooperativeness and Reward Dependence, and Cooperativeness and Self-Directedness were intercorrelated. The Cronbach's coefficients were between .60 and .85 on temperament dimensions, and were between .82 and .83 on character dimensions. The lowest Cronbach's coefficients were found in Reward Dependence (.60) and Persistence (.62). A principal axis factor analysis with a four-factor solution by Oblimin rotation reproduced highest loadings on Novelty Seeking and Harm Avoidance and relatively weaker loadings on Reward Dependence and Persistence. A three-factor solution for character subscales reproduced highest loadings on Cooperativeness and Self-Transcendence.
Conclusions: The reliability and validity of the Turkish version of the TCI were supported by its reliable psychometric properties and construct validity. The Turkish version of the TCI successfully confirmed Cloninger's seven-factor model of personality. This pioneering work suggests that the Turkish TCI can be applied in clinical populations as well as in neurobiological and neuroimaging investigations.

Key words: personality, temperament, character, TCI, cross-cultural

Bull Clin Psychopharmacol 2004;14:107-131

[1]Uzm. Dr. Medical University of South Carolina, Department of Psychiatry and Behavioral Sciences, Charleston, SC, USA,
[2]Doç. Dr. Bakırköy Eğitim ve Araştırma Hastanesi Servis Şefi, Bakırköy, İstanbul-Türkiye
[3]Prof. Dr., KTÜ Tıp Fakültesi Psikiyatri AD,Trabzon-Türkiye, [5]Uzm. Dr. Devlet Hastanesi Psikiyatri Servisi, Trabzon-Turkey
[4]Doç. Dr., [6]Prof. Dr. Atatürk Üniversitesi Tıp Fakültesi Psikiyatri AD, Erzurum-Turkey,
[7]Phd, Prof. Dr. Augusta State University, Department of Psychology, Augusta, GA, USA,
[8]PhD, Yard. Doç, [9]MD, Prof. Dr. Washington University School of Medicine, Department of Psychiatry, St Louis, MO, USA

Yazışma Adresi / Address reprint requests to:
Samet Köse, MD, Medical University of South Carolina, Department of Psychiatry and Behavioral Sciences, Center for Advanced Imaging Research, 67 President Street Room No: 502 N, Charleston, SC 29425

Elektronik posta adresi / E-mail address:
kose@musc.edu

Kabul tarihi / Date of acceptance:
28 Mayıs 2004 / May 28, 2004

Giriş

Cloninger, kişiliğin iki temel bileşeni olarak mizaç ile karakterdeki normal ve anormal varyasyonları açıklayan boyutsal bir psikobiyolojik kişilik modeli geliştirmiş ve tanımlamıştır (1-3). Cloninger'in kişilik kuramı psikometrik kişilik çalışmaları kadar, ikizler ve ailelerde yapılan uzunlamasına gelişimsel çalışmalar, nörofarmakolojik ve nörodavranışsal öğrenme çalışmalarından elde edilen bilgilerin sentezi üzerine kurulmuştur. Cloninger'in psikobiyolojik kuramının, mizaç ve karakter boyutlarının detaylı tanımları Köse tarafından önceki bir gözden geçirme yazısında sunulmuştur (4).

Cloninger'in psikobiyolojik kişilik kuramı Yenilik Arayışı (NS), Zarardan Kaçınma (HA), Ödül Bağımlılığı (RD) ve Sebat Etme (P) olmak üzere dört boyutlu bir mizaç tanımlar. Bu mizaç özellikleri

*Bu çalışmanın ön bulguları 22-27 Ekim 2002 tarihleri arasında Marmaris'te yapılan 38. Ulusal Psikiyatri Kongre'sinde poster olarak sunulmuştur.

algıya dayalı huylar ve becerilerde bireysel farklılıkları yansıtır ve genetik olarak homojen ve birbirinden bağımsız olarak kalıtımsaldır.

Yenilik Arayışı (NS); davranışsal aktivasyon sistemi ile bağlantılıdır ve yeni bir uyarana yanıtta keşfedici bir etkinliğe doğru kalıtsal bir yatkınlık, dürtüsel karar verme, ödül ipuçlarına yaklaşımda aşırıya kaçma, çabucak öfkelenme ile engellenmeden aktif kaçınmayı içerir. Zarardan Kaçınma (HA), davranışsal inhibisyon sistemi ile ilişkilidir ve davranışın önlenmesi ya da durdurulmasına dair kalıtsal bir eğilimdir. Kendisini gelecek sorunlara ilişkin karamsar bir endişelilik hali, belirsizlik korkusu, yabancılardan çekinme ve çabuk yorulma gibi pasif kaçınma davraşları ile gösterir. Ödül Bağımlılığı (RD), davranışsal sürdürme sistemi ile ilişkilidir ve duygusallık, sosyal bağlanma, başkalarının onayına bağımlılık ile kendisini gösteren kalıtsal bir eğilimdir. Sebat Etme (P) ise, engellenme ve yorgunluğa karşın sebat etmeye olan kalıtsal eğilimdir. Sebat eden bireyler insanı engelleyen ödül yokluğu durumlarıyla karşılaştıklarında bu davranışın sönmesine karşı direnç sergilerler (1-4).

Cloninger'in modeli ayrıca Kendini yönetme (SD), İş Birliği Yapma (C) ile Kendini aşma (ST) olmak uzere üç boyutlu bir karakter tanımı içerir. İnsanın yaşı ilerledikçe bu karakter bileşenleri benlik kavramları ile uğraşma ya da erişkinlikle gelen kişisel veya sosyal etkinliliğin getirdikleriyle olgunlaşır. Karakter bileşenlerinin mizaç bileşenlerinden farklı olarak daha çok kültürel olarak kalıtıldıklarına inanılır. Kendini Yönetme (SD), kişinin kendi tercihleri konusunda sorumluluğunu kabul etmesi, bireysel açıdan anlamlı amaçların belirlenmesi ve sorunları çözmede beceri ve güvenin gelişimi ile kendini kabullenmeden oluşur. Kendini yöneten birey otonom bir bireydir, sorumludur, amaçları vardır, beceriklidir, kabul edicidir ve görev duygusu olan bir bireydir. İş Birliği Yapma (C) sosyal kabul, empati, yararlılık, sevecenlik ve erdemli-vicdanlı olmaktan oluşur. İş birliği yapan insanlar hoşgörülü, empati yeteneği olan, yararlı, sevecen ve erdemlidirler. Kendini Aşma (ST) kendilik kaybı, kişilerarası özdeşim ve manevi kabulden oluşur. Kendini aşan bireyler yaratıcı, bencil olmayan, inançlı, manevi duyguları olan, idealist bireylerdir (1-4).

Cloninger'in kişilik kuramı, kişiliği çok katlı düzeyde anlayabilmek için bize kapsamlı bir içgörü sunar. Bu düzeyler kişiliğin genetiği, davranışın nörobiyolojik temelleri, bilişsel duygusal yapı ve kişiliğin gelişimi, kişilik boyutlarındaki bireysel farklılıkların davranışsal karşılıkları ve kişilik yapılarıyla gelişimsel etmenlerin etkileşiminin psikiyatrik bozukluklara yatkınlığa yol açması gibi düzeylerdir (1-4).

Cloninger'in kişilik modeli davranış görünümleri ile nörotransmiterler arasında bağlantı kurma olanağı da verir. Yenilik Arayışı (davranışsal aktivasyon) ile dopamin, Zarardan Kaçınma (davranışsal inhibisyon) ile serotonin ve Ödül Bağımlılığı (davranışı sürdürme) ile norepinefrin, Sebat Etme (davranışta israr etme) ile glutamaterjik aktivite arasında ilişki olduğu bildirilmiştir (1-4,5).

Cloninger'in kişilik kuramını temel alarak geliştirdiği Mizaç ve Karakter Envanteri (Temperament and Character Inventory; TCI), 240 maddeden oluşan "Doğru" ya da "Yanlış" şeklinde yanıtlanan bir kendini değerlendirme ölçeğidir, zaman sınırlandırması yoktur, 20-30 dakikada doldurulabilir. Bu yedi-faktörlü kişilik envanterinin geçerlik ve güvenirliği hem genel popülasyonda hem de psikiyatrik hastalarda sınanmış ve replike edilmiştir. TCI içerik olarak 15 yaş ve üstü bireyler için uygulama alanına sahiptir. TCI'nin 125 maddeli kısa formu, 140 maddeli formu ve en yaygın olarak kullanılan 240 maddeli formu mevcuttur (3).

TCI çeşitli dillere çevrilmiş, çeşitli kültürlerde geçerlik ve güvenirliği gösterilmiştir. Şu ana değin İsveççe (6), Hollandaca (7,8), Çekçe (9), Almanca (10), Fransızca (11), Japonca (12), İspanyolca (13), Polonyaca (14), Korece (15), Çince (16) versiyonları geliştirilmiş ve psikometrik özellikleri bildirilmistir. İtalyanca versiyonu psikiyatrik hasta örnekleminde çalışılmış (17), geçerlik-güvenirligi henüz yapılmamıştır. TCI'nin ayrıca Belçika (18) ve Avustralya toplumlarında geçerlik güvenirliği bildirilmiştir (19). TCI'nin Türkçeye kazandırılma işlemi 2001 yılında Samet Köse ve Kemal Sayar tarafından gerçekleştirilmiş ve bu versiyon Türkçe TCI olarak Cloninger tarafından onaylanmıştır.

Bu çalışmanın amacı Türkçe TCI'nin Türk toplumu normatif değerlerine ulaşmak, ölçeğin iç tutarlık, geçerlik-güvenirlik ve faktör yapısını sağlıklı gönüllülerden oluşan bir örneklem grubunda araştırmaktır. Bu çalışma Türkiye'de TCI kullanılarak yapılan çalışmaların öncülüdür.

Yöntem

Örneklem: Çalışmanın örneklemi Trabzon Karadeniz Teknik Üniversitesi Tıp Fakültesi ile Erzurum Atatürk Üni-

versitesi Tıp Fakültesinde hayatlarının hiçbir doneminde ruhsal rahatsızlık nedeniyle tedavi görmemiş sağlıklı gönüllülerden oluştu. Çalışmaya 683 denek alındı (363 erkek, 320 kadın, yaş ortalaması 26.25±10.84 (yaş dağılımı 18-75).

Trabzon çalışma grubu 349 denekten oluştu (166 erkek, 183 kadın, yaş ortalaması 20.75±2.54 (yaş dağılımı 18-55). Erzurum çalışma grubu 334 denekten oluştu (197 erkek, 137 kadın, yaş ortalaması 32.15±12.73 (yaş dağılımı 19-75).

Örneklem grubu ulaşılabilirlik-elverişlilik ilkelerine göre seçildi, deneklere çalışmaya katılmaları nedeniyle herhangi bir ücret ödenmedi. Örneklem grubunun yaş, medeni durum, meslek, eğitim, yaşadıkları kent ve ekonomik durum dağılımları Tablo 1'de verilmiştir.

Çalışma hakkında bilgilendirildikten sonra çalışmaya katılanlardan etik kurul onayına uygun bir şekilde bilgilendirilmiş onay alındı. Yaşları 18 ile 75 arasında olan denekler, Türkçe okuma ve yazma yeterliliği olan denekler, eğitim durumu ölçeği anlamaya ve yanıtlamaya uygun olan denekler çalışmaya alındı. Majör depresif bozukluk, psikoz, anksiyete bozukluğu, obsesif–kompulsif bozukluk, post–travmatik stres bozukluğu, otizm, mental retardasyon, suisid girişimi gibi ciddi bir psikiyatrik hastalık öyküsü olan, madde kötüye kullanımı/ bağımlılığı olan, beyin hasarı ile sonuçlanan travma (bilinç kaybıyla sonuçlanan kafa travması, bir gece hastanede kalış ya da diğer nörolojik sekeller), serebrovasküler hastalık, tümörler, nöbetler (çocukluk çağı febril konvülziyonu dışında) menenjit, ensefalit ya da anormal beyin tomografisi (CT) ya da magnetik rezonans görüntüleme (MRI) öyküsü olan, çalışmanın yapıldığı zamandan 5 hafta öncesinden itibaren herhangi bir psikotrop ilaç kullanan denekler çalışmaya alınmadı.

Tablo 1. Örneklem grubunun demografik özellikleri

	n	%
Cinsiyet		
Kadın	320	46.9
Erkek	363	53.1
Yaş		
18-25	577	84.5
26-49	85	12.4
50-91	21	3.1
Medeni Durum		
Evli	154	22.5
Bekar	528	77.3
Boşanmış	1	.1
Eğitim Durumu		
İlkokul	21	3.1
Ortaokul	11	1.6
Lise	60	8.8
Üniversite	591	86.5
Mesleki Durum		
Ev hanımı	3	.4
Öğrenci	517	75.7
Memur	116	17.0
İşçi	20	2.9
Özel meslek	24	3.5
İşsiz	2	.3
Emekli	1	.1
Yerleşim Yeri		
Köy	12	1.8
Kasaba	19	2.8
Kent	652	95.5
Ekonomik Durum		
Düşük	38	5.6
Orta	547	80.1
Yüksek	98	14.3

Araçlar: Deneklere, sosyodemografik özelliklerini sorgulayan anket formu ve sağlık durumlarının değerlendirmesini sağlayan tıbbi öykü değerlendirme formu, sosyal olarak arzu edilir yanıtlar verilip verilmediğini sınamak amacıyla Marlowe-Crowne Sosyal Arzu Edilirlik Ölçeği Kısa Formu (20) ve ardından TCI verildi. Marlowe-Crowne Sosyal Arzu Edilirlik Ölçeği Samet Köse tarafından Türkçeleştirilerek kullanıldı. Sosyal olarak arzu edilir yanıtlar veren deneklerin TCI profilleri geçersiz kabul edilerek çalışma dışında tutuldu. Yine TCI'nin 230. maddesi olan "Bu anket formunda çok yalan söyledim" maddesini "Doğru" olarak yanıtlayan deneklerin TCI profilleri geçersiz kabul edildi ve çalışma dışında tutuldu.

Mizaç ve Karakter Envanteri (Temperament and Character Inventory; TCI). TCI'nin İngilizce formu (Version 9, 240 madde) Samet Köse tarafından Türkçe'ye çevrilmiş, tersine çevirme ("back-translation") işlemi ölçeği daha önce hiç görmemiş olan Kemal Sayar tarafından yapılmıştır("blind-translation"). Tersine çevrilen ölçek Samet Köse tarafından asıl ölçekle karşılaştırılıp, anlaşılmasında zorluk olan maddeler sadeleştirilerek değiştirilmiştir. TCI'nin Türkçe uyarlamasının kültürel geçerliliğini sağlamak için, kültürel açıdan anlaşılması zor maddelerin varlığı dikkatle incelenmiş, Türk toplumu normlarına uymayan bir maddeye rastlanmamıştır. Ölçeğin son şekli yazarlarca geçerlik-güvenirlik çalışmasında kullanılmak üzere Cloninger tarafından onaylanmıştır (Köse ve Sayar, 2001).

Ölçek "Doğru" ya da "Yanlış" şeklinde yanıtlanan 240 maddeden oluşan bir kendini değerlendirme ölçeğidir. TCI 7 üst-sıra (higher order) ölçekten oluşmaktadır. Sebat Etme (P) dışında tüm boyutlar, üç ve beş arasında

alt ölçeklere ayrılmıştır. Mizaç boyutunda Yenilik Arayışı (NS) 4 alt ölçeğe, Zarardan Kaçınma (HA) 4 alt ölçeğe, Ödül Bağımlılığı (RD) 4 alt ölçeğe, karakter boyutunda Kendini yönetme (SD) 5 alt ölçeğe, İşbirliği Yapma (C) 5 alt ölçeğe, Kendini aşma (ST) 3 alt ölçeğe ayrılmıştır.

Ölçekler alt ölçekler toplamından oluşmaktadır. Örneğin, Toplam Yenilik Arayışı (NS) puanı = NS1 + NS2 + NS3 + NS4 şeklinde hesaplanır. Mizaç boyutu 12 alt-sıra (lower-order) alt ölçekten oluşmaktadır. Yenilik Arayışı (NS, 40 madde), NS1 (Keşfetmekten heyecan duyma, 11 madde), NS2 (Dürtüsellik, 10 madde), NS3 (Savurganlık, 9 madde) ve NS4 (Düzensizlik, 10 madde) alt ölçeklerinden oluşmaktadır. Zarardan Kaçınma (HA, 35), HA1 (Beklenti endişesi, 11 madde), HA2 (Belirsizlik korkusu, 7 madde), HA3 (Yabancılardan çekinme, 8 madde) ve HA4 (Çabuk yorulma ve dermansızlık, 9 madde) alt ölçeklerinden oluşmaktadır. Ödül Bağımlılığı (RD, 24 madde), RD1 (Duygusallık, 10 madde), RD3 (Bağlanma, 8 madde) ve RD4 (Bağımlılık, 6 madde) alt ölçeklerinden oluşmaktadır. Sebat Etme (P, 8) alt ölçeği olmayan tek mizaç ölçeğidir. Karakter boyutu 13 alt-sıra (lower-order) alt ölçekten oluşmaktadır. Kendini yönetme (SD, 44 madde), SD1 (Sorumluluk alma, 8 madde), SD2 (Amaçlılık, 8 madde), SD3 (Beceriklilik, 5 madde), SD4 (Kendini kabullenme, 11 madde) ve SD5 (Uyumlu ikincil huylar, 12 madde) alt ölçeklerinden oluşmaktadır. İş Birliği Yapma (C, 42 madde), C1 (Sosyal onaylama, 8 madde), C2 (Empati duyma, 7 madde), C3 (Yardımseverlik, 8 madde), C4 (Acıma, 10 madde) ve C5 (Erdemlilik-vicdanlılık, 9 madde) alt ölçeklerinden oluşmaktadır. Kendini aşma (ST, 33), ST1 (Kendilik kaybı, 11 madde), ST2 (Kişiler ötesi özdeşim, 9 madde) ve ST3 (Manevi kabullenme, 13 madde) alt ölçeklerinden oluşmaktadır.

TCI ölçeğinin değerlendirilmesinde bazı maddeler tersine puanlanmaktadır. Bu maddelerin puanlanmasında Yanlış seçeneği işaretlenen maddeler 1 puan alır. Ayrıca TCI içinde yer aldıkları halde puanlanmayan maddeler de mevcuttur (Madde 69,75,101,111,118, 134,140,170,176,190,213,230,239,240).

İstatistiksel Yöntem: Mizaç ve Karakter Envanteri (TCI)'nin ölçek ve altölçek ortalama ve standard sapma değerleri çalışmacılar tarafından geliştirilen Windows Temelli Türkçe TCI programı (Türkçe TCI Programı. Versiyon 1.0. Samet Köse ve Korkmaz Başgök, 2003) ile hesaplandı. Tüm veriler analiz için SPSS for Windows 12.0 programına yüklendi. Türk toplumu ortalama değerleri Cloninger'in orijinal data değerleri ile One-Sample t test kullanılarak karşılaştırıldı. Korelasyon analizleri Pearson korelasyon testi ile incelendi. Ölçeğin iç tutarlığı Cronbach Alfa yöntemiyle değerlendirildi. Hipotez sınamalarında anlamlı alfa değeri $p<0.05$ olarak seçildi. Ölçeğin faktör yapısını irdeleyebilmek için önce yedi-faktörlü çözüme göre Kaiser normalizasyonlu oblimin dönüştürmesine göre Temel Eksen Faktör Analizi (Principal Axis Analysis) uygulandı. Ardından mizaç ve karakter boyutlarına ayrı ayrı Temel Eksen Faktör Analizi yapıldı. Ölçeğin ortogonal faktör yapısını sınaması ve ilerki çalışmalarda kullanılmak için faktör yapısı ayrıca Kaiser normalizasyonlu varimax dönüştürmesine göre Temel Bileşenler Faktör Analizi (Principal Component Analysis) ile incelendi.

Bulgular

TCI ortalamalarının Amerikan toplumu ortalamaları ile karşılaştırılması

TCI ölçeklerinin Türk ve Amerikan toplumu ortalama ve standart sapmaları Tablo 2'de verilmiştir.

Mizaç boyutunda; Türk toplumu ortalama Yenilik Arayışı (NS) ile Savurganlık (NS3), Düzensizlik (NS4) alt ölçekleri, Ödül Bağımlılığı (RD) ile Duygusallık (RD1), Bağlanma (RD3) ve Bağımlılık (RD4) alt ölçekleri puanları Amerikan toplumu ortalamalarından anlamlı olarak düşük bulundu ($p<0.001$). Zarardan Kaçınma (HA) ile Beklenti endişesi (HA1), Belirsizlik korkusu (HA2), Çabuk yorulma ve dermansızlık (HA4) alt ölçekleri puanları Amerikan toplumu ortalama değerlerinden anlamlı olarak yüksek bulundu ($p<0.001$).

Karakter boyutunda; Kendini yönetme (SD) ile Sorumluluk alma (SD1), Beceriklilik (SD3) ve Kendini kabullenme (SD4), İş Birliği Yapma (C) ile Sosyal onaylama (C1), Empati duyma (C2), Yardımseverlik (C3), Acıma (C4) ve Erdemlilik-vicdanlılık (C5), Kendini Aşma (ST) ile Manevi kabullenme (ST3) puanları Amerikan toplumu ortalamalarından anlamlı olarak düşük bulundu ($p<0.001$). Amaçlılık (SD2) ve Kişiler ötesi özdeşim (ST2) alt ölçekleri puanları Amerikan toplumu ortalama değerlerinden anlamlı olarak yüksek bulundu ($p<0.001$).

Brändström ve arkadaşlarının (1998) çalışmasında olduğu gibi ortalama ve standart sapmaları %10'un üstünde olan ölçek ve alt ölçekler dikkate alındığında, HA4

Tablo 2. Türk ve Amerikan toplumu deneklerinde TCI ölçek ve alt ölçeklerinin ortalama (M) ve standart sapmaları (SD)

	Türk (n=683)		Amerikalı (n=300)	
**Ölçek**	**M**	**SD**	**M**	**SD**
**Yenilik Arayışı**	18.5**	5.00	19.2	6.0
NS1 (Keşfetmekten heyecan duyma)	6.3	1.9	6.3	2.3
NS2 (Dürtüsellik)	3.6	1.9	3.7	2.2
NS3 (Savurganlık)	4.6**	2.1	5.0	2.3
NS4 (Düzensizlik)	3.9**	1.8	4.3	2.1
**Zarardan Kaçınma**	16.8**	6.4	12.6	6.8
HA1 (Beklenti endişesi)	5.6**	2.3	3.2	2.4
HA2 (Belirsizlik korkusu)	4.1**	1.9	3.6	2.0
HA3 (Yabancılardan çekinme)	3.4	2.2	3.3	2.3
HA4 (Çabuk yorulma ve dermansızlık)	3.6**	2.3	2.5	2.2
**Ödül Bağımlılığı**	14.1**	3.2	15.5	4.4
RD1 (Duygusallık)	6.9**	1.9	7.3	2.1
RD3 (Bağlanma)	4.5**	1.9	4.7	2.3
RD4 (Bağımlılık)	2.7**	1.4	3.5	1.6
**Sebat Etme**	4.8	1.9	5.6	1.9
**Kendini Yönetme**	29.1**	6.2	30.7	7.5
SD1 (Sorumluluk alma)	5.1**	1.9	5.8	2.0
SD2 (Amaçlılık)	6.0**	1.6	5.5	1.8
SD3 (Beceriklilik)	3.4**	1.3	4.0	1.2
SD4 (Kendini kabullenme)	5.7**	2.6	6.4	2.8
SD5 (Uyumlu ikincil huylar)	8.9	2.0	9.0	2.5
**İş Birliği Yapma**	29.4**	5.9	32.3	7.2
C1 (Sosyal onaylama)	6.3**	1.7	6.7	1.5
C2 (Empati duyma)	4.3**	1.5	5.3	1.4
C3 (Yardımseverlik)	4.8**	1.4	6.3	1.6
C4 (Acıma)	7.1**	2.7	7.6	2.8
C5 (Erdemlilik-vicdanlılık)	6.9**	1.4	6.5	2.0
**Kendini Aşma**	18.6*	5.4	19.2	6.3
ST1 (Kendilik kaybı)	5.9	2.2	5.9	2.7
ST2 (Kişiler ötesi özdeşim)	4.9**	2.1	4.6	2.4
ST3 (Manevi kabullenme)	7.8**	2.8	8.7	2.9

*p < 0.01
**p < 0.001

Tablo 3. Türkçe TCI ölçekler ve yaş arasında Pearson korelasyon değerleri

Ölçek	NS	HA	RD	P	SD	C	ST	Yaş
Yenilik Arayışı (NS)								
Zarardan Kaçınma (HA)	-.198**							
Ödül Bağımlılığı (RD)	-.002	.025						
Sebat Etme (P)	-.271**	-.094*	.003					
Kendini Yönetme (SD)	-.113**	-.437**	.073	.080*				
İş Birliği Yapma (C)	-.087*	-.182**	.365**	.072	.427**			
Kendini Aşma (ST)	.090*	.038	.113**	.182**	-.277**	.048		
Yaş	-.223**	-.004	.078*	.135**	-.123**	-.024	.034	

*p < 0.05
**p < 0.01

(Çabuk yorulma ve dermansızlık), RD4 (Bağımlılık), SD1 (Sorumluluk alma), SD3 (Beceriklilik), SD4 (Kendini kabullenme), C2 (Empati duyma), C3 (Yardımseverlik) ve ST3 (Manevi kabullenme) puanlarının Amerikan toplumu ortalama değerlerinden farklı olduğu saptandı (p<0.001).

TCI ölçeklerinin kendi aralarında ve yaş ile korelasyonu

Türkçe TCI mizaç ve karakter boyutları arasındaki korelasyon ve bunların yaş ile korelasyonu Tablo 3'de gösterilmiştir. Kendini Yönetme ile Zarardan Kaçınma (r= -.437, p< .01), İş Birliği Yapma ile Ödül Bağımlılığı (r= .365, p< .01) ve İş Birliği Yapma ile Kendini yönetme (r= .427, p< .01) korelasyon katsayıları Cloninger'in orijinal çalışmasındaki normatif değerlerle doğrudan karşılaştırma yapmak amacıyla seçilen .30 kesme değerinin üstünde bulunmuştur. Diğer tüm korelasyon katsayıları zayıf bir ilişki göstermiştir. Yaş ile Yenilik Arayışı (r= -

.223, p< .01) ve Kendini Yonetme (r= -.123, p< .05) arasında negatif yönde, Ödül Bağımlılığı (r= .078, p< .05) ve Sebat Etme (r= .135, p< .01) arasında pozitif yönde korelasyon saptandı. Yaş artarken Yenilik Arayışı ve Kendini yönetme puanları düşmekte, Ödül Bağımlılığı ve Sebat Etme puanları yükselmekteydi.

TCI ölçekleri üzerinde cinsiyetin etkisi

Örneklem grubunda kadın erkek dağılımı görece birbirine yakındı (320 kadın, %46.9; 363 erkek, %53.1). Mizaç boyutunda, kadınlarda Yenilik Arayışı (M= 18.93, t= 2.11, df= 681, p< .05), Zarardan Kaçınma (M= 17.79, t= 3.99, df= 681, p< .001), Ödül Bağımlılığı (M= 14.56, t= 3.39, df= 681, p< .01) puanları erkeklerden anlamlı olarak daha yüksek bulundu. Karakter boyutunda, İşbirliği Yapma puanları kadınlarda erkeklerden anlamlı olarak daha yüksek bulundu (M= 29.95, t= 2.40, df= 681, p< .05).

İç tutarlık, geçerlik ve güvenirlik

Türkçe TCI ölçek ve alt ölçeklerinin iç tutarlık, geçerlik ve güvenirliği Cronbach alfa yöntemi kullanılarak hesaplandı. TCI ölçek ve alt ölçeklerinin iç tutarlık katsayıları Tablo 4'de gösterilmiştir. TCI ölçeklerinin Cronbach alfa değerleri mizaç boyutunda .60 ile .85, karakter boyutunda .82 ile .83 arasında bulundu. En düşük Cronbach alfa katsayıları Ödül Bağımlılığı (.60) ve Sebat Etme (.62) olarak bulundu.

Türkçe TCI alt ölçeklerinin Cronbach alfa değerleri sırasıyla NS1 (.52), NS2 (.61), NS3 (.62), NS4 (.45), HA1 (.58), HA2 (.63), HA3 (.71), HA4 (.69), RD1 (.60), RD3 (.52), RD4 (.67), SD1 (.54), SD2 (.53), SD3 (.51), SD4 (.72), SD5 (.61), C1 (.63), C2 (.49), C3 (.61), C4 (.76), C5 (.44), ST1 (.70), ST2 (.69) ve ST3 (.59) olarak bulundu ve genel olarak her bir ölçek ile tutarlılık gösterdi. Erdemlilik-vicdanlılık (C5) en düşük Cronbach alfa değerine sahipti (.44). Yine Düzensizlik (NS4) alt ölçeği (.45) ve Empati duyma (C2) alt ölçeğinin (.49) Cronbach alfa değerleri görece düşük bulundu. Yenilik Arayışı altölçekleri .45 ile .62 arasında geniş bir değişkenlik gösterdi. Ödül Bağımlılığı alt ölçekleri .52 le .67 değerleri arasında değişkenlik göster-

Tablo 4. Türkçe TCI karşılaştırmalı Cronbach alfa değerleri

Ölçek	Madde sayısı	Türk (n=683)	Amerikalı (n=300)
**Yenilik Arayışı**	40	.74	.78
NS1 (Keşfetmekten heyecan duyma)	11	.52	.60
NS2 (Dürtüsellik)	10	.61	.62
NS3 (Savurganlık)	9	.62	.71
NS4 (Düzensizlik)	10	.45	.54
**Zarardan Kaçınma**	35	.85	.87
HA1 (Beklenti endişesi)	11	.58	.71
HA2 (Belirsizlik korkusu)	7	.63	.69
HA3 (Yabancılardan çekinme)	8	.71	.76
HA4 (Çabuk yorulma ve dermansızlık)	9	.69	.72
**Ödül Bağımlılığı**	24	.60	.76
RD1 (Duygusallık)	10	.52	.62
RD3 (Bağlanma)	8	.67	.72
RD4 (Bağımlılık)	6	.42	.57
**Sebat Etme**	8	.62	.65
**Kendini Yönetme**	44	.83	.86
SD1 (Sorumluluk alma)	8	.54	.70
SD2 (Amaçlılık)	8	.53	.58
SD3 (Beceriklilik)	5	.51	.57
SD4 (Kendini kabullenme)	11	.72	.75
SD5 (Uyumlu ikincil huylar)	12	.61	.75
**İş Birliği Yapma**	42	.82	.89
C1 (Sosyal onaylama)	8	.63	.64
C2 (Empati duyma)	7	.49	.47
C3 (Yardımseverlik)	8	.61	.63
C4 (Acıma)	10	.76	.86
C5 (Erdemlilik-vicdanlılık)	9	.44	.65
**Kendini Aşma**	33	.82	.84
ST1 (Kendilik kaybı)	11	.70	.73
ST2 (Kişiler ötesi özdeşim)	9	.69	.72
ST3 (Manevi kabullenme)	13	.59	.74

Tablo 5. Çeşitli dillerde TCI Cronbach alfa değerleri

Ölçek	İsveç (n=1300) Sağlıklı (1998)	Hollanda (n=148) Sağlıklı (1998)	Fransa (n=602) Sağlıklı (2000)	Japonya (n=461) Sağlıklı (2000)	İspanya (n=416) Psikiyatrik Hastalar (2001)	Kore (n=851) Üniversite Öğrencisi (2002)	Çin* (n=535) Sağlıklı (2003)	Almanya (n=509) Sağlıklı (1999)	Türkiye (n=683) Sağlıklı (2004)
NS	.78	.79	.75	.71	.75	.78	.69	.77	.74
HA	.85	.88	.87	.85	.86	.85	.81	.84	.85
RD	.62	.72	.68	.73	.67	.68	.56	.69	.60
P	.56	.65	.49	.64	.49	.60	.79	.57	.62
SD	.81	.83	.82	.83	.87	.87	.81	.84	.83
C	.75	.81	.81	.82	.83	.82	.73	.84	.82
ST	.84	.88	.84	.82	.85	.85	.83	.78	.82

*144-itemli TCI kullanılarak yapılmış çalışma

di. Zarardan Kaçınma alt ölçekleri .58 ile .71 arasında değişen kararlı Cronbach alfa değerleri gösterdiler. Kendini Yönetme alt ölçekleri .51 ile .72 değerleri arasında değişkenlik gösterdi. İş Birliği Yapma alt ölçekleri .44 ile .76 değerleri arasında en geniş değişkenlik gösterdiler. Son olarak, Kendini Aşma alt ölçekleri .59 ile .70 arasında değişkenlik gösterdi. 24 alt ölçekten sekizi standart .60 değerinin altında bulundu. Çeşitli kültürlerde yapılan TCI çalışmalarında ölçek ve alt ölçeklerin iç tutarlık katsayıları Tablo 5'de gösterilmiştir.

Türkçe TCI faktör yapısı

Türkçe TCI ölçek ve altölçeklerinin faktör yapısını sınamak için onaylayıcı faktör analizi ("confirmatory factor analysis") olarak temel eksen analizi uygulandı. Cloninger'in orijinal çalışmasına uygunluk göstermesi açısından Kaiser normalizasyonlu oblimin dönüştürümlü faktör analizi uygulandığında yedi faktör yerine altı faktörlü bir yapı elde edildi. Bulgular Tablo 6'da sunulmuştur.

Temel eksen analizi sonucu Türkçe TCI ölçeği için 6 faktör belirlendi. Faktör 1 SD, Faktör 2 C, Faktör 3 ST, Faktör 4 NS, Faktör 5 HA, Faktör 6 RD olarak belirlendi. Faktör 1'e HA1 ve HA4 negatif yönde yüklemede bulunduğu görüldü. Faktör 3'e RD1 pozitif yönde, SD1 ve SD4 ise negatif yönde yüklemede bulunduğu görüldü. Faktör 4'e P negatif yönde güçlü yüklemede bulunduğu görüldü. Faktör 5'e RD4 pozitif yönde yüklemede bulundu. Faktör 6'da RD1 ve RD4'ün görece düşük yüklemede bulunduğu, bunun yanı sıra C2 ve C3'ün yüklemede bulunduğu görüldü. HA'un iki alt ölçeğinin (HA1 ve HA4) negatif yönde SD'e, RD'in iki alt ölçeginin (RD1 ve RD4) sırasıyla ST'e ve HA'a pozitif yönde yüklemede bulunduğu görüldü.

Bu bulgularla ölçeğin faktör yapısının yedi-faktörlü TCI faktör yapısını tekrarlamadığı düşünüldü. Mizaç genetik olarak belirlenen ve algılara bağlı huylar ve beceriler iken, karakter kavramlara dayalı amaçlar ve değerlerdeki bireysel farklılıkları yansıtır. Cloninger'in psikobiyolojik kişilik modeli mizaç ve karakter ölçekleri arasındaki ilişkinin ikincil emosyonlar doğuracağını ve bunun da kişiliğin gelişiminde önemli olduğunu vurgulamaktadır.

Bu nedenle Türkçe TCI ölçeği mizaç ve karakter boyutları ayrı ayrı olarak oblimin dönüştürümlü faktör analizi ile incelendi. Bulgular Tablo 7a ve Tablo 7b'de sunulmuştur.

Mizaç boyutunda, Yenilik Arayışı (NS) ve Zarardan Kaçınma (HA) alt ölçeklerinin oldukça güçlü yüklemde bulundukları saptandı. Ödül Bağımlılığı (RD) ve Sebat Etme (P) alt ölçeklerinin göreceli olarak daha zayıf yüklemede bulunduğu saptandı. Keşfetmekten heyecan duyma (NS1) negatif olarak Faktör 1'e (Zarardan Kaçınma), pozitif olarak da Faktör 3'e (Ödül Bağımlılığı) yük sağlıyordu. Bağımlılık (RD4) negatif olarak Faktör 4'e (Sebat Etme) yük sağlıyordu. Bu dört faktör toplam varyansın sırasıyla %22.34, %16.03, %10.70 ve %8.75, kümülatif olarak da %57.81'ini açıklıyordu.

Bileşen korelasyon matrisinde Faktör 1 ile Faktör 2 arasında -.111, Faktör 1 ile Faktör 3 arasında -.139, Faktör 1 ile Faktör 4 arasında -.051 olarak, Faktör 2 ile Faktör 3 arasında -.023, Faktör 2 ile Faktör 4 arasında -.077, Faktör 3 ile Faktör 4 arasında .016 şeklinde düşük korelasyon bulundu.

Karakter boyutunda, İşbirliği Yapma (C) ve Kendini aşmanın (ST) oldukça tutarlı ve güçlü yüklemede bulundukları gözlendi. Kendini yönetme (SD), Kendini kabullenme (SD4) alt ölçeği dışında güçlü yük verdi. Ken-

Tablo 6. TCI mizaç ve karakter ölçeklerinin faktör yapı matrisi ("factor structure matrix")

Ölçek	Faktör 1 SD	Faktör 2 C	Faktör 3 ST	Faktör 4 NS	Faktör 5 HA	Faktör 6 RD
Özdeğer (Eigenvalue)	4.362	2.693	2.349	1.886	1.171	1.107
Varyans (%)	17.4	10.8	9.4	7.5	4.7	4.4
NS1 (Keşfetmekten heyecan duyma)	.298	.120	.174	.274	**-.439**	**.455**
NS2 (Dürtüsellik)	-.204	-.245	.055	**.664**	.034	-.173
NS3 (Savurganlık)	-.047	.097	-.033	**.658**	-.150	.188
NS4 (Düzensizlik)	-.127	-.348	.178	**.599**	-.237	.009
HA1 (Beklenti endişesi)	**-.400**	-.092	.105	-.193	**.536**	-.074
HA2 (Belirsizlik korkusu)	-.201	.004	-.029	-.212	**.767**	-.144
HA3 (Yabancılardan çekinme)	-.193	-.256	.002	.064	**.708**	-.356
HA4 (Çabuk yorulma ve dermansızlık)	**-.513**	-.172	.164	.091	**.578**	-.114
RD1 (Duygusallık)	-.137	.251	**.510**	-.264	.216	.340
RD3 (Bağlanma)	.075	.036	-.079	.074	-.173	**.765**
RD4 (Bağımlılık)	.152	.211	-.080	.043	**.476**	.357
Sebat Etme	.194	-.036	.320	**-.612**	-.172	-.031
SD1 (Sorumluluk alma)	**.610**	.367	**-.414**	-.043	-.276	.157
SD2 (Amaçlılık)	**.707**	.227	-.026	-.261	-.078	.192
SD3 (Beceriklilik)	**.722**	.158	-.066	.003	-.370	.083
SD4 (Kendini kabullenme)	.251	**.581**	**-.401**	.037	-.090	.070
SD5 (Uyumlu ikincil huylar)	**.666**	.227	-.119	-.200	.000	.197
C1 (Sosyal onaylama)	.210	**.696**	-.011	.005	-.111	.198
C2 (Empati duyma)	.288	**.512**	.118	.106	-.092	**.452**
C3 (Yardımseverlik)	.287	**.429**	-.086	-.062	.062	**.591**
C4 (Acıma)	.010	**.750**	.117	-.231	.013	.252
C5 (Erdemlilik-vicdanlılık)	.341	**.621**	.195	-.134	.201	.063
ST1 (Kendilik kaybı)	-.257	-.140	**.703**	.002	-.087	-.163
ST2 (Kişiler ötesi özdeşim)	-.115	.138	**.722**	-.274	-.123	.121
ST3 (Manevi kabullenme)	.109	-.020	**.686**	.238	.093	-.097

Mutlak değerleri .40 ve üstündeki yükleme değerleri koyu renkle gösterilmiştir.

Tablo 7a. TCI mizaç alt ölçeklerinin faktör yapı matrisi ("factor structure matrix")

Ölçek	Faktör 1	Faktör 2	Faktör 3	Faktör 4
Özdeğer (Eigenvalue)	2.681	1.923	1.283	1.050
Varyans (%)	22.34	16.03	10.70	8.75
NS1 (Keşfetmekten heyecan duyma)	**-.497**	.258	**.558**	.052
NS2 (Dürtüsellik)	.096	**.724**	-.173	-.036
NS3 (Savurganlık)	-.164	**.651**	.229	-.181
NS4 (Düzensizlik)	-.122	**.697**	.023	.192
HA1 (Beklenti endişesi)	**.692**	-.118	.074	.092
HA2 (Belirsizlik korkusu)	**.764**	-.213	-.144	-.156
HA3 (Yabancılardan çekinme)	**.682**	.020	-.396	-.186
HA4 (Çabuk yorulma ve dermansızlık)	.711	.171	-.077	-.105
RD1 (Duygusallık)	.324	-.232	**.633**	.111
RD3 (Bağlanma)	-.274	.139	**.619**	-.275
RD4 (Bağımlılık)	.151	-.181	.126	**-.791**
Sebat Etme	-.042	**-.535**	.235	**.574**

Kaiser normalizasyonu ile oblimin rotasyonu uygulanmıştır.
Mutlak değerleri .40 ve üstündeki yükleme değerleri koyu renkle gösterilmiştir.

dini kabullenmenin (SD4), pozitif yönde İşbirliği Yapma'ya (C), negatif yönde Kendini Aşma'ya (ST) yük verdiği görüldü. Sorumluluk almanın (SD1), negatif yönde Kendini Aşma'ya (ST) yük verdiği görüldü. Bu üç faktör toplam varyansın sırasıyla %25.50, %15.31 ve %9.96, kümülatif olarak da %50.76'ini açıklıyordu.

Bileşen korelasyon matrisinde Faktör 1 ile Faktör 2 arasında -.042, Faktör 2 ile Faktör 3 arasında -.153 olarak düşük, Faktör 1 ile Faktör 3 arasında .332 olarak yüksek korelasyon bulundu. Bu bulgu SD4'un ikili yüklemesinin yansıması olarak yorumlanmıştır.

Türkçe TCI ölçek ve altölçeklerinin faktör yapısını sınamak için ayrıca araştırıcı faktör analizi ("exploratory factor analysis") olarak Kaiser normalizasyonlu varimax

Tablo 7b. TCI karakter mizaç alt ölçeklerinin faktör yapı matrisi ("factor structure matrix")

Ölçek	Faktör 1	Faktör 2	Faktör 3
Özdeğer (Eigenvalue)	3.314	1.991	1.295
Varyans (%)	25.50	15.31	9.96
SD1 (Sorumluluk alma)	.352	**-.435**	**.667**
SD2 (Amaçlılık)	.302	-.034	**.733**
SD3 (Beceriklilik)	.172	-.079	**.761**
SD4 (Kendini kabullenme)	**.519**	**-.434**	.269
SD5 (Uyumlu ikincil huylar)	.271	-.121	.711
C1 (Sosyal onaylama)	**.685**	-.058	.252
C2 (Empati duyma)	**.595**	.051	.288
C3 (Yardımseverlik)	**.565**	-.145	.294
C4 (Acıma)	**.758**	.054	.044
C5 (Erdemlilik-vicdanlılık)	**.617**	.136	.290
ST1 (Kendilik kaybı)	-.121	**.739**	-.268
ST2 (Kişiler ötesi özdeşim)	.195	**.755**	-.060
ST3 (Manevi kabullenme)	-.013	**.704**	.012

Kaiser Normalizasyonu ile Oblimin dönüştürmesi uygulanmıştır.
Mutlak değerleri .40 ve üstündeki yükleme değerleri koyu renkle gösterilmiştir.

Tablo 8a. TCI mizaç alt ölçeklerinin faktör bileşen matrisi ("rotated component matrix")

Ölçek	Faktör 1	Faktör 2	Faktör 3	Faktör 4
Özdeğer (Eigenvalue)	2.681	1.923	1.283	1.050
Varyans (%)	22.34	16.03	10.70	8.75
NS1 (Keşfetmekten heyecan duyma)	**-.443**	.217	**.544**	-.062
NS2 (Dürtüsellik)	.124	**.736**	-.128	-.030
NS3 (Savurganlık)	-.115	**.640**	.260	.127
NS4 (Düzensizlik)	-.078	**.690**	.050	-.251
HA1 (Beklenti endişesi)	**.703**	-.080	.111	-.110
HA2 (Belirsizlik korkusu)	**.748**	-.166	-.105	.147
HA3 (Yabancılardan çekinme)	**.656**	.069	-.351	.162
HA4 (Çabuk yorulma ve dermansızlık)	**.722**	.214	-.021	.063
RD1 (Duygusallık)	.369	-.229	**.644**	-.110
RD3 (Bağlanma)	-.226	.112	**.619**	.267
RD4 (Bağımlılık)	.136	-.171	.142	**.803**
Sebat Etme	-.041	**-.547**	.194	**-.530**

Kaiser normalizasyonu ile varimax rotasyonu uygulanmıştır.
Mutlak değerleri .40 ve üstündeki yükleme değerleri koyu renkle gösterilmiştir.

dönüştürümlü temel bileşenler analizi uygulandı. TCI Mizaç alt ölçeklerinin faktör yapısı Tablo 8a'da gösterilmiştir. Varimax dönüştürümlü temel bileşenler analizinde ("principal component analysis"), TCI Mizaç boyutunda sırasıyla Zarardan Kaçınma (HA), Yenilik Arayışı (NS), Ödül Bağımlılığı (RD) ve Sebat Etme'nin (P) oldukça güçlü yük verdikleri gözlendi. Yenilik Arayışı ölçeğinde, diğer üç bileşenin yükleri oldukça tutarlı iken Keşfetmekten heyecan duyma (NS1) alt ölçeğinin zayıf bir yük sağladığı bulundu. NS1'in negatif yönde Faktör 1'e, pozitif yönde Faktör 3'e orta derecede yük verdiği gözlendi. Duygusallık (RD1) ve Bağlanma (RD3) alt ölçekleri Faktör 3'e güçlü biçimde yük verirken, Bağımlılık (RD4) bileşeninin yük vermediği gözlendi. RD4 güçlü biçimde Faktör 4'e yük verdi. Sebat Etme'nin (P) negatif biçimde Faktör 2'ye yük verdiği gözlendi. Bu dört faktör toplam varyansın sırasıyla %22.34, %16.03, %10.70 ve %8.75, kümülatif olarak da %57.81'ini açıklıyordu.

Bileşen dönüştürüm matris değerleri Faktör 1 ile faktör 2 arasında –.262, Faktör 1 ile Faktör 3 arasında -.297, Faktör 1 ile Faktör 4 arasında .128 şeklinde bulundu. Bileşen dönüştürüm matris değerleri Faktör 2 ile Faktör 3 arasında -.158, Faktör 2 ile Faktör 4 arasında .172 bulundu. Bileşen dönüştürüm matris değeri Faktör 3 ile Faktör 4 arasında .486 bulundu.

Varimax dönüştürümlü temel bileşenler analizinde, TCI Karakter boyutunda, İş Birliği Yapma (C) ve Kendini Aşma (ST) alt ölçeklerinin oldukça güçlü yük verdikleri gözlendi. Kendini Yönetme (SD) alt ölçeklerinden dördü oldukça tutarlı yüklemede bulunurken, Kendini kabullenmenin (SD4) düşük derecede yük verdiği gözlendi. SD4 Faktör 1'e pozitif, Faktör 3'e negatif yönde orta de-

Tablo 8b. TCI karakter alt ölçeklerinin faktör bileşen matrisi ("rotated component matrix")

Ölçek	Faktör 1	Faktör 2	Faktör 3
Özdeğer (Eigenvalue)	3.314	1.991	1.295
Varyans (%)	25.50	15.31	9.96
SD1 (Sorumluluk alma)	.251	**.604**	-.389
SD2 (Amaçlılık)	.191	**.715**	.021
SD3 (Beceriklilik)	.050	**.765**	-.022
SD4 (Kendini kabullenme)	**.493**	.152	**-.420**
SD5 (Uyumlu ikincil huylar)	.162	**.690**	-.069
C1 (Sosyal onaylama)	**.672**	.133	-.042
C2 (Empati duyma)	**.573**	.197	.071
C3 (Yardımseverlik)	**.538**	.193	-.126
C4 (Acıma)	**.783**	-.091	.055
C5 (Erdemlilik vicdanlılık)	**.596**	.201	.157
ST1 (Kendilik kaybı)	-.075	-.202	**.725**
ST2 (Kişiler ötesi özdeşim)	.220	-.040	**.757**
ST3 (Manevi kabullenme)	-.010	.070	**.711**

Kaiser Normalizasyonu ile Varimax dönüştürmesi uygulanmıştır.
Mutlak değerleri .40 ve üstündeki yükleme değerleri koyu renkle gösterilmiştir.

recede yük vermekteydi. Bu üç faktör toplam varyansın sırasıyla %25.50, %15.31 ve %9.96, kümülatif olarak da %50.76'ini açıklıyordu. Bileşen dönüştürüm matris değerleri Faktör 1 ile Faktör 2 arasında .646, Faktör 1 ile Faktör 3 arasında -.293 şeklinde bulundu. Bileşen dönüştürüm matris değeri Faktör 2 ile Faktör 3 arasında .864 bulundu. TCI karakter alt ölçeklerinin varimax dönüştürümlü faktör yapısı Tablo 8b'de gösterilmiştir.

Tartışma

Bu çalışmanın sonuçları mizaç boyutunda; Türk toplumu ortalama Yenilik Arayışı (NS) ve Savurganlık (NS3), Düzensizlik (NS4) alt ölçekleri, Ödül Bağımlılığı (RD), Duygusallık (RD1), Bağlanma (RD3) ve Bağımlılık (RD4) alt ölçekleri puanları Amerikan toplumu ortalamalarından anlamlı olarak düşük olduğunu, Zarardan Kaçınma (HA), Beklenti endişesi (HA1), Belirsizlik korkusu (HA2), Çabuk yorulma ve dermansızlık (HA4) alt ölçekleri puanlarının Amerikan toplumu ortalama değerlerinden anlamlı olarak yüksek olduğunu gösterdi. Karakter boyutunda; Kendini yönetme (SD) ve Sorumluluk alma (SD1), Beceriklilik (SD3), Kendini kabullenme (SD4), İş Birliği Yapma (C), Sosyal onaylama (C1), Empati duyma (C2), Yardımseverlik (C3), Acıma (C4) Erdemlilik vicdanlılık (C5), Kendini aşma (ST), Manevi kabullenme (ST3) puanları Amerikan toplumu ortalamalarından anlamlı olarak düşük bulundu. Amaçlılık (SD2) ve Kişiler ötesi özdeşim (ST2) alt ölçekleri puanları Amerikan toplumu ortalama değerlerinden anlamlı olarak yüksek bulundu.

Ortalama ve standart sapmaları %10'un üstünde olan ölçek ve alt ölçekler dikkate alındığında, HA4 (Çabuk yorulma ve dermansızlık), RD4 (Bağımlılık), SD1 (Sorumluluk alma), SD3 (Beceriklilik), SD4 (Kendini kabullenme), C2 (Empati duyma), C3 (Yardımseverlik), ST3 (Manevi kabullenme) alt ölçeklerinin Amerikan toplumu ortalama değerlerinin farklı olduğunu gösterdi. Bu bulgular İsveç, Hollanda, Fransa gibi Avrupa ülkelerinin ortalama değerlerine yakınlık göstermesi ve Amerikan toplumu ortalama değerlerinden farklı olması açısından anlamlıdır. Her dilde normatif değerlerin elde edilmesi ve karşılaştırmalarda o kültüre ait değerlerin kullanılmasını desteklemektedir. Özellikle karakter ölçeklerinin Türk ve Amerikan toplumlarında farklılıklar göstermesi kültürel farklılıklar ve bu kavramların tanımının Batılı olmayan toplumlarda farklı değerlendirilmesi ile açıklanabilir. Ayrıca örneklem grubunun farklı oluşturulması ile de açıklanabilir. Cloninger Amerikan toplumu normatif değerlerini bir alışveriş merkezine gelen 150 kadın, 150 erkek 300 sağlıklı gönüllü ile elde etmişti (2). Bizim çalışmamızda denekler iki ayrı üniversite bünyesinden elverişlilik ilkesine göre seçildi. Ayrıca Cloninger'in orijinal çalışmasına oranla örneklem grubumuzun daha çok gençlerden oluşmasının da bu farklılıkta payı olabileceği düşünüldü (Türk örneklem grubu yaş aralıkları; 18-25, n=577, %84.5, 26-49, n=85, %12.4, 50-91, n=21, %3, Amerikan örneklem grubu yaş aralıkları; 18-25, n=86, %29, 26-49, n=181, %60, 50-91, n=33, %11).

Benzer biçimde, Brändström ve arkadaşları İsveç toplumunda Kendini Aşma (ST) puanlarının Amerikan toplumuna oranla daha düşük saptanmasının, İsveç ve ABD toplumları arasındaki kültürel farklılıklardan kay-

naklandığını ileri sürdüler (6). De la Rie ve arkadaşları Hollanda toplumu ortalama Yenilik Arayışı (NS) ve Kendini Yönetme (SD) değerlerinin Amerikan toplumu değerlerinden yüksek olduğunu, Sebat Etme (P) ve Kendini Aşma (ST) değerlerinin ise daha düşük olduğunu gösterdiler (7). Kijima ve arkadaşlarının çalışmasında (12), Japon deneklerde Amerikan toplumuna oranla Zarardan Kaçınma (HA) yüksek, Ödül Bağımlılığı (RD) düşük, Kendini Yönetme (SD) ve İşbirliği Yapma (C) puanları düşük bulunmuştur. Japon toplumu geleneksel olarak hiyerarşik ve babaerkil olarak tanımlanır ve bağımsız bireylerden çok birbirine bağlı bireylerden oluşur. Kijima ve arkadaşları da Japon denekleri zarardan daha çok kaçınan ve daha az otonom bireyler olarak yorumladılar. İşbirliği Yapabilme (C) ve Ödül Bağımlılığı (RD) puanlarının düşük bulunmasını açıklamakta zorluk çekilmiş, Japon halkının kişiliğinin çatısı içinde bu durumun empatik ve sosyal olarak birbirine bağlı olunmasıyla açıklanabileceği ileri sürülmüştür (12). Benzer biçimde bireyselliğin değil de birbirine bağımlı olmanın yüceltildiği, kolektivist yönelimli bir toplum olan Türk toplumunda C2 (Empati duyma), C3 (Yardımseverlik), ST3 (Manevi kabullenme) puanlarının düşük oluşunu açıklamak zordur, bu kavramların tanımlarının Batılı olmayan toplumlarda farklı değerlendirilmesinden kaynaklanabileceği düşünülmektedir.

Türkçe TCI mizaç ve karakter boyutları arasındaki korelasyon örüntülerinde, Kendini Yönetme ile Zarardan Kaçınma arasında negatif yönde anlamlı bir ilişki, İş Birliği Yapma ile Ödül Bağımlılığı ve İş Birliği Yapma ile Kendini Yönetme arasında pozitif yönde anlamlı bir ilişki bulunmuştur. Diğer tüm korelasyon katsayıları zayıf bir ilişki göstermiştir. Kendini Yönetme puanlarının düşük oluşu ile Zarardan Kaçınma'nın yüksek oluşu anlamlıdır. Zarardan Kaçınma puanları yüksek bireyler sakıngan, pasif, kendine güveni olmayan, karamsar, kötümser bireyler olarak tanımlanmaktadır (2,3). Kendini Yönetme puanları düşük bireyler suçlayıcı, amaçsız, beceriksiz, kendi kendileriyle uğraşan, disiplinsiz bireyler olarak tanımlanmaktadırlar (2,3). Bu ilişki bu tür bireylerin kendi davranışlarının kendi istekleri etkisi dışında geliştiğini düşünen bireyler olduklarını, kendi başarısızlıklarından diğer insanları ve durumları sorumlu tutmaya eğilimli olduklarını düşündürmektedir. İş Birliği Yapma'ya yatkınlık ise, hem Ödül Bağımlılığı'nı, hem de Kendini Yönetme'yi beraberinde getirmektedir. Benzer korelasyon örüntüsü diğer tüm kültürlerdeki TCI çalışmalarında da elde edilmiştir. Yaş ile Yenilik Arayışı ve Kendini Yönetme arasında negatif yönde, Ödül Bağımlılığı ve Sebat Etme arasında pozitif yönde korelasyon saptandı. Yaş artarken Yenilik Arayışı ve Kendini Yönetme puanları düşmekte, Ödül Bağımlılığı ve Sebat Etme puanları yükselmekteydi. Cloninger'in kişilik kuramında mizaç boyutları kalıtsaldır, erken çocukluk dönemi ile ilişkilidir, karakter boyutları ise yaşla birlikte olgunlaşır. Yaş ile Yenilik Arayışı'nın ters yönde ilişkisi anlamlıdır, yeni uyaranlara ilgi azalması ile açıklanır, Yenilik Arayışı puanları her on yılda bir puan azalmaktadır (21). Çalışmamızda yaşın artmasıyla Kendini Yönetme puanlarının beklendiği gibi artmayıp azaldığı saptanmış, bu durumun örneklem grubunun büyük ölçüde gençlerden oluşması ve yaşlı bireylerin sayıca az olmalarına bağlanmıştır. Aynı şekilde yaş artarken Ödül Bağımlılığı ve Sebat Etme puanlarının yükselmesi bu iki ölçeğin diğer iki mizaç boyutu kadar yerleşmiş olmayışı ve ek alt ölçeklere gereksinimin varlığı ile açıklanmıştır. Bu bulgular Cloninger'in yaş ile TCI karakter boyutları arasında varolduğunu ileri sürdüğü bağlantıyı ılımlı düzeyde desteklemiştir (2).

Cinsiyetin TCI ölçekleri üzerindeki etkisi araştırıldığında, kadınlarda mizaç boyutunda Yenilik Arayışı, Zarardan Kaçınma ve Ödül Bağımlılığı puanlarının erkeklerden anlamlı olarak yüksek olduğu saptandı. Karakter boyutunda ise İşbirliği Yapma puanları kadınlarda erkeklere oranla daha yüksekti. Kadınlarda erkeklere oranla daha yüksek Ödül Bağımlılığı puanları olması gerek Amerikalı gerekse diğer kültürlerde tutarlılıkla saptanmış bir bulgudur. Cloninger bu bulgunun noradrenerjik sistemlerle ilişkili olduğunu ileri sürmüştür (1). Buss, kadın beyninin erkeklerinkine oranla daha erken evrime uğramalarının annelik becerisine katkısı olacağını vurgulamış ve bu olgunun hayatta kalma değeri olduğunu ileri sürmüştür (22). Cloninger'in orijinal çalışmasında kadınlar daha yüksek İşbirliği Yapma ve Manevi kabullenme (ST3) puanları gösterdiler (2). Diğer kültürlerdeki çalışmalarda, De La Rie ve arkadaşları Hollanda toplumunda Zarardan Kaçınma, İşbirliği Yapma ve Kendini Aşma puanlarının kadınlarda erkeklere oranla daha yüksek olduğunu bildirdiler (7). Yine Hollanda toplumunda sağlıklı denekler ve psikiyatri kliniğine ayaktan başvuranlarda yapılan bir çalışmada Duijens ve ark. kadınlarda erkeklere oranla daha yüksek

Zarardan Kaçınma, Ödül Bağımlılığı ve İşbirliği Yapma ve daha düşük Sebat Etme puanları saptadılar (8). Pelissolo ve Lepine, Fransız toplumunda kadınların erkeklere oranla daha yüksek Zarardan Kaçınma, Ödül Bağımlılığı ve Kendini Aşma, daha düşük Kendini Yönetme puanları olduğunu bildirdiler (11). Bu bulgular cinsiyetin mizaç ve karakter boyutuna etkilerini göstereceği gibi, kültürel farklılıklardan da kaynaklanabileceğini göstermiştir. Çalışmamızda cinsiyete dayalı bulgular Amerikan toplumundan çok Avrupa toplumlarının bulgularına paralellik göstermekteydi.

Türkçe TCI ölçek ve alt ölçeklerinin iç tutarlık, geçerlik ve güvenirliği kabul edilebilir düzeyde bulundu. Türkçe TCI ölçeklerinin Cronbach alfa değerleri mizaç boyutunda .60 ile .85, karakter boyutunda .82 ile .83 arasında bulundu. En düşük Cronbach alfa katsayıları Ödül Bağımlılığı (.60) ve Sebat Etme (.62) olarak bulundu. Kendini aşma alt ölçekleri .59 ile .70 arasında değişkenlik gösterdi ve 24 alt ölçekten yalnızca sekizi standart .60 değerinin altında bulundu. Sebat Etme (P), TCI öncülü TPQ'da Ödül Bağımlılığı'nın alt ölçeklerinden biri olarak yer alıyordu, diğer alt ölçeklerle yeterli korelasyon göstermediği düşünülerek dördüncü mizaç faktörü olarak TCI'ne eklendi (2,20). Diğer kültürlerde yapılan TCI çalışmalarında Sebat Etme iç tutarlılığının genel olarak düşük oluşu, diğer ölçeklerle zayıf bağlantılar göstermesi başlangıçta bu ölçeğin madde sayısının yalnızca 8 gibi düşük oluşuna, çeviri sorunlarına bağlanmış, bağımsız bir değişken olarak yeterli psikometrik özellik göstermediği düşünülmüş, ancak bu kısıtlamalar Mizaç ve Karakter Envanteri Gözden Geçirilmiş Formu (TCI-R) gelişimi sırasında gözönünde bulundurulmuş ve Ödül Bağımlılığı'na dördüncü bir alt ölçek eklenmiş, Sebat Etme ise 35 maddeden oluşan dört alt ölçekli bir ölçeğe dönüştürülmüştür (23). Mizaç ve Karakter Envanteri Gözden Geçirilmiş Formu (Türkçe TCI-R) Köse ve Sayar tarafından çevrilerek dilimize kazandırılmış ve bu çeviri Cloninger tarafından onaylanmıştır (2004), geçerlik-güvenirlik çalışması sürmektedir.

Türkçe TCI ölçek ve alt ölçeklerinin faktör yapısını sınamak için onaylayıcı faktör analizi ("confirmatory factor analysis") olarak temel eksen analizi seçilmesinin temel nedeni, Cloninger'in kişilik modelinde mizaç ve karakter boyutlarının yaşam boyu birbiriyle etkileşimde bulunmaları ve ortogonal faktör yapısıyla uyuşma göstermeyişleridir. Kaiser normalizasyonlu oblimin dönüştürümlü faktör analizi 25 alt ölçeğe birlikte uygulandığında, yedi faktörlü yapıyı tekrar etmediği gözlendi. Temel Eksen Analizi sonucu Türkçe TCI ölçeği icin 6 faktör belirlendi ve Sebat Etme (P) ayrı bir faktör olarak belirmedi. Yalnızca De la Rie ve arkadaşlarının yaptığı çalışmada (7), mizaç ve karakter boyutlarının bir arada yedi-faktörlü çözümü gösterilmiştir. Diğer tüm çalışmalarda yedi faktörlü çözüm çıkarmada başarısız olunmuş, mizaç ve karakter boyutları ayrı ayrı faktör analizine tabi tutulmuştur. Mizaç ve Karakter Envanteri (yedi boyutuyla) karmaşık ancak dinamik bir sistemi temsil eder, bu nedenle yaygın biçimde kullanılan faktör analizi için gerekli olan "basit yapı"nın ötesinde yer alırlar. Mizaç ve karakter boyutlarını ayrı ayrı almak bu basit yapıya yakınlaştıracak ve farklı gruplarda tekrarlanabilir faktörleri verecektir (Przybeck ve Cloninger, kişisel yazışma).

Mizaç genetik olarak belirlenen ve algılara bağlı huylar ve becerileri, karakter ise kavramlara dayalı amaçlar ve değerlerdeki bireysel farklılıkları yansıtır. Cloninger'in psikobiyolojik kişilik modeli mizaç ve karakter ölçekleri arasındaki ilişkinin ikincil emosyonlar doğuracağını ve bunun da kişiliğin gelişiminde önemli olduğunu vurgulamaktadır. Bizim çalışmamızda da gerek mizaç, gerekse karakter boyutlarında, her bir ölçekle ilgili alt ölçekler en yüksek faktör yüklerini tek bir boyuta vermişlerdir.

Mizaç boyutunda, üç faktörlü çözüme göre, Yenilik Arayışı ve Zarardan Kaçınma alt ölçeklerinin oldukça güçlü yüklemde bulundukları saptandı. Ödül Bağımlılığı ve Sebat Etme'nin göreceli olarak daha zayıf yüklemden bulunduğu saptandı. Keşfetmekten heyecan duyma (NS1) negatif olarak Zarardan Kaçınma'ya, pozitif olarak da Ödül Bağımlılığı'na yük sağlıyordu. Bağımlılık (RD4) negatif olarak Sebat Etme'ye yük sağlıyordu. Bu dört faktör toplam varyansın sırasıyla %22.34, %16.03, %10.70 ve %8.75, kümülatif olarak da %57.81'ini açıklıyordu. Bu değerler diğer dillerdeki çalışmalara yakın bulunmuştur. Mizaç boyutu faktörleri tarafından açıklanan varyans Brändström ve arkadaşları (6) tarafından %58.9, Richter ve arkadaşları (10) tarafından %61.8, Pelissolo ve Lepine (11) tarafından %53, Gutierrez ve arkadaşları (13) tarafından %60.3, Hansenne ve ark. tarafından (18) %53, Sung ve arkadaşları (15) tarafından %61.2, Parker ve arkadaşları (16) tarafından %54.5 olarak bulunmuştur.

Karakter boyutunda, İşbirliği Yapma (C) ve Kendini Aşma'nın (ST) oldukça tutarlı ve güçlü yüklemede bulundukları gözlendi. Kendini Yönetme (SD), Kendini ka-

bullenme (SD4) dışında güçlü yük verdi. Kendini kabullenme (SD4), pozitif yönde İşbirliği Yapma'ya, negatif yönde Kendini Aşma'ya yük verdi. Sorumluluk alma (SD1) negatif yönde Kendini Aşma'ya yük verdi. Cloninger bu bulguları "kişinin kendi sınırlılıklarını kabul etme yeteneği, diğer insanların sınırlamalarını kabul etme ve bunlara hoşgörü gösterme yeteneğiyle ilişkilidir" şeklinde yorumlamıştır. Bu üç faktör toplam varyansın sırasıyla %25.50, %15.31 ve %9.96, kümülatif olarak da %50.76'ini açıklıyordu. Bu değerler diğer dillerdeki çalışmalara göre düşük bulunmuştur. Karakter boyutu faktörleri tarafından açıklanan varyans Brändström ve arkadaşları (6) tarafından %48, Richter ve arkadaşları (10) tarafından %53, Pelissolo ve Lepine (11) tarafından %53, Gutierrez ve arkadaşları (13) tarafından %57.3, Hansenne ve ark. (18) tarafından %54, Sung ve arkadaşları (15) tarafından %56.1, Parker ve arkadaşları (19) tarafından %55.2 olarak bulunmuştur.

TCI ölçekleri arasındaki ilişki her ne kadar ortogonal olmasa da, Türkçe TCI ölçek ve alt ölçeklerinin faktör yapısını sınamak ve gelecekteki çalışmalarda karşılaştırmalı veri sağlaması için araştırıcı faktör analizi ("exploratory factor analysis") olarak Kaiser normalizasyonlu varimax dönüştürümlü temel bileşenler analizi uygulandı. TCI Mizaç boyutunda, Zarardan Kaçınma (HA), Yenilik Arayışı (NS), Ödül Bağımlılığı (RD) ve Sebat Etme'nin (P) oldukça güçlü yük verdikleri gözlendi. Yenilik Arayışı ölçeğinde, diğer üç bileşenin yükleri oldukça tutarlı iken Keşfetmekten heyecan duyma (NS1) alt ölçeğinin zayıf bir yük sağladığı bulundu. NS1'in negatif yönde Zarardan Kaçınma'ya, pozitif yönde Ödül Bağımlılığı'na orta derecede yük verdiği gözlendi. Duygusallık (RD1) ve Bağlanma (RD3) alt ölçekleri Ödül Bağımlılığı'na güçlü biçimde yük verirken, Bağımlılık (RD4) bileşeninin yük vermediği gözlendi. RD4 güçlü biçimde Sebat Etme'ye yük verdi. Sebat Etme'nin ise negatif biçimde Yenilik Arayışı'na yük verdiği gözlendi. Bu dört faktör toplam varyansın sırasıyla %22.34, %16.03, %10.70 ve %8.75, kümülatif olarak da %57.81'ini açıklıyordu. Bu değerler yukarda bildirilen diğer dillerdeki çalışmalara yakın bulunmuştur.

Varimax dönüştürümlü temel bileşenler analizinde, karakter boyutunda, İş Birliği Yapma (C) ve Kendini aşma (ST) ölçeklerinin oldukça güçlü yük verdikleri gözlendi. Kendini Yönetme (SD) alt ölçeklerinden dördü oldukça tutarlı yüklemede bulunurken, Kendini kabullenme (SD4) düşük derecede yük verdi. SD4 Faktör 1'e pozitif, Faktör 3'e negatif yönde orta derecede yük vermekteydi. Bu üç faktör toplam varyansın sırasıyla %25.50, %15.31 ve %9.96, kümülatif olarak da %50.76'ini açıklıyordu. Bu değerler yukarda bildirilen diğer dillerdeki çalışmalara yakın bulunmuştur.

Sonuç

Sonuç olarak, bu çalışma Türkçe TCI kullanılarak yapılan çalışmaların ilki ve öncülüdür. Türkçe TCI ölçeğinin geçerlik ve güvenirliği, ölçek puanlarının dağılımı, yüksek iç tutarlık katsayısı ve yapı geçerliği tarafından desteklenmiştir. Bu çalışma Türkçe TCI'nin Türk toplumunda Cloninger'in yedi-faktörlü kişilik modelini başarıyla ölçebileceğini göstermektedir. Ölçeğin faktör yapısı dört mizaç, üç karakter boyutunu tutarlı biçimde çıkarmıştır. Bu çalışmanın sonuçları TCI'nin yalnızca Batılı toplumlara sınırlı olmayıp, geniş bir uygulanabilirliği olduğunu ve bileşen yapısının evrenselliğini desteklemektedir. Türkçe TCI'nin Türk toplumunda sağlıklı deneklerde geçerlik-güvenirliğini gösteren bu çalışma, ölçeğin normal kişilik yapısını incelemenin yanı sıra psikiyatrik hasta popülasyonunda klinik uygulamalarda, gen polimorfizmi ve beyin görüntüleme çalışmalarında kullanımı açısından yararlı bir araç olduğunu göstermektedir.

Türkçe TCI kişiliğin genetik temelinden, davranışın nörobiyolojik temellerine, kişiliğin bilişsel ve emosyonel yapısı ve gelişiminden, kişilik boyutlarında bireysel farklılıkların davranışsal bağlarına, kişilik yapılarının gelişimsel etmenlerle etkileşiminden, psikiyatrik bozukluklara yol açmasına uzanan çok sayıda alanda kapsamlı bilgi sağlama potansiyeline sahiptir.

Ülkemizde Türkçe TCI çok sayıda merkezde psikiyatrik hasta popülasyonunda çalışılmaktadır. Boz ve arkadaşları kronik gerilim tipi baş ağrılı hastalarda Türkçe TCI'ni başarıyla kullanmışlardır (24). Köse ve arkadaşları Türkçe TCI'nin geçerlik güvenirliği ve anksiyete ve depresyon yordayıcısı olarak kullanılabilirliğini çalışmışlardır (25-27). Evren ve Köse yatarak tedavi gören bir grup erkek alkol bağımlısı hastada TCI ve aleksitimi ilişkisini araştırmışlardır (yayına hazırlanmakta). Gimzal ve arkadaşları obsesif-kompülsif bozuklukta, Çelikel ve Köse majör depresyonlu olgularda, Çetin ve Köse sağlıklı subay ve erlerde Türkçe TCI'nin psikometrik özelliklerini halen incelemektedir. Ayrıca Köse ve Sayar, Gözden Ge-

çirilmiş Türkçe TCI (TCI-R), Köse ve Çelikel ise halen Çocuklar İçin Türkçe TCI (Junior-TCI) ölçeklerinin geçerlik-güvenirliği ve faktör yapısını çalışmaktadırlar. Bu çalışmanın yayınlanmasının Türkçe TCI'ne olan mevcut ilgiyi daha da artıracağı düşünülmektedir.

Teşekkür / Acknowledgement: *Yazarlar TCI ölçek ve alt ölçek puanlarının hesaplanmasında ve Windows Temelli Türkçe TCI programının yazılımında katkılarından dolayı Korkmaz Başgök'e teşekkür ederler.*

Kaynaklar:

1. Cloninger CR. A systematic method for clinical description and classification of personality variants. A proposal. Arch Gen Psychiatry 1987; 44: 573-588

2. Cloninger CR, Svrakic DM, Przybeck TR. A psychobiological model of temperament and character Archives of General Psychiatry 1993; 50: 975–990

3. Cloninger CR, Przybeck TR, Svrakic DM, Wetzel RD. The Temperament and Caracter Inventory (TCI): A guide to its development and use. Center for Psychobiology of Personality. Department of Psychiatry, Washington University School of Medicine, 1994

4. Köse S. A Psychobiological Model Of Temperament And Character: TCI. Yeni Symposium 2003; 41: 86-97

5. Peirson AR, Heuchert JW, Thomala L, Berk M, Plein H, Cloninger CR. Relationship between serotonin and the temperament and character inventory. Psychiatry Res 1999; 89: 29-37

6. Brändström S, Schlette P, Przybeck TR, Lundberg M, Forsgren T, Sigvardsson S, Nylander PO, Nilsson LG, Cloninger CR, Adolfsson R. Swedish normative data on personality using the Temperament and Character Inventory Comprehensive Psychiatry 1998; 39: 122–128

7. De la Rie SM, Duijsens IJ, Cloninger CR. Temperament, character, and personality disorders. J Personal Disord 1998; 12: 362-372

8. Duijsens IJ, Spinhoven P, Goekoop JG. The Dutch temperament and character inventory (TCI): dimensional structure, reliability and validity in a normal and psychiatric outpatient sample. Personality and Individual Differences 2000; 28: 487-499

9. Kozeny J, Höschl C. The temperament and character inventory: psychometric ıntegrity of the Czech version. Studia Psychologica 1999; 41: 123-132

10. Richter J, Brandstrom S, Przybeck TR. Assessing personality: The temperament and character inventory in a cross-sectional comparison between Germany, Sweden, and the USA. Psychol Rep 1999; 84: 1315-1330

11. Pelissolo A, Lepine JP. Normative data and factor structure of the Temperament and Character Inventory (TCI) in the French version. Psychiatry Res 2000; 94: 67-76

12. Kijima N, Tanaka E, Suzuki N, Higuchi H, Kitamura T. Reliability and validity of the Japanese version of the Temperament and Character Inventory. Psychol Rep 2000; 86: 1050-1058

13. Gutierrez F, Torrens M, Boget T, Martin-Santos R, Sangorrin J, Perez G, Salamero M. Psychometric properties of the Temperament and Character Inventory (TCI) questionnaire in a Spanish psychiatric population. Acta Psychiatr Scand 2001; 103: 143-147

14. Zakrzewska M, Samochowiec J, Rybakowski F, Hauser J, Pelka-Wysiecka J. [Polish version of Temperament and Character Inventory (TCI): the analysis of reliability.] Psychiatr Pol 2001; 35: 455-465

15. Sung SM, Kim JH, Yang E, Abrams KY, Lyoo IK. Reliability and validity of the Korean version of the Temperament and Character Inventory. Compr Psychiatry 2002; 43: 235-243

16. Parker G, Cheah YC, Parker K. Properties of the temperament and character inventory in a Chinese sample. Acta Psychiatr Scand 2003; 108: 367-373

17. Fassino S, Abbate-Daga G, Leombruni P, Amianto F, Rovera G, Rovera GG. Temperament and character in italian men with anorexia nervosa: a controlled study with the temperament and character inventory. J Nerv Ment Dis 2001; 189: 788-794

18. Hansenne, M, Le Bon O, Gauthier A, Ansseau M. Belgian normative data of the temperament and character inventory. European Journal of Psychological Assessment 2001; 17: 56–62

19. Parker G, Hadzi-Pavlovic D, Parker K, Malhi G, Mitchell P, Wilhelm K, Austin MP. An Australian validation study of the temperament and character inventory. Acta Psychiatr Scand 2003; 108: 359-366

20. Reynold WM. Development of reliable and short forms of the Marlowe-Crowne Social Desirability Scale. Journal of Clinical Psychology 1982; 38: 119-125

21. Cloninger CR, Przybeck TR, Svrakic DM. The Tridimensional Personality Questionnaire: U.S. normative data. Psychol Rep 1991; 69(3 Pt 1): 1047-1057

22. Buss DM. Evolutionary psychology: the new science of mind. Boston: Allyn and Bacon, 1999

23. Brandstrom S, Richter J, Nylander PO. Further development of the Temperament and Character Inventory. Psychol Rep 2003; 93: 995-1002

24. Boz C, Sayar K, Velioglu S, Hocaoglu C, Alioglu Z, Yalman B, Ozmenoglu M. [Temperament and character profile of patients with chronic tension-type headache]. Turk Psikiyatri Derg. 2004; 15: 105-111

25. Köse S, Sayar K, Kalelioğlu Ü, Aydın N, Reeves RA, Cloninger CR. Mizaç ve Karakter Envanteri (TCI)'nin Geçerlik ve Güvenirliği. 38. Ulusal Psikiyatri Kongresi. 2002; 22-27 Ekim, Marmaris, Türkiye

26. Köse S, Kalelioğlu Ü, Sayar K, Reeves RA, Cloninger CR. Depresyon ve anksiyete belirleyicisi olarak Mizaç ve Karakter Envanteri (TC)'nin Kullanımı. 38. Ulusal Psikiyatri Kongresi. 2002;22-27 Ekim, Marmaris, Türkiye

27. Kalelioğlu Ü. Depresyon ve anksiyete belirleyicisi olarak Mizaç ve Karakter Envanteri (TC)'nin Kullanımı. Karadeniz Üniversitesi Tıp Fakültesi Psikiyatri Anabilim Dalı, Trabzon, Türkiye. Uzmanlık Tezi, 2003

EK-1
Mizaç ve Karakter Envanteri

(Türkçe TCI)

Bu anket formunda kişilerin kendi tutumlarını, görüşlerini, ilgilerini ya da kişisel duygularını tanımlarken kullanabilecekleri ifadeleri bulacaksınız.

Her ifade DOĞRU ya da YANLIŞ olarak yanıtlanabilir. İfadeleri okuyunuz ve hangi seçeneğin sizi en iyi tanımladığına karar veriniz. Sadece şu anda nasıl hissettiğiniz değil, ÇOĞU KEZ ya da genellikle nasıl davrandığınız ve hissettiğinizi tanımlamaya çalışınız.

Bu anket formunu kendi başınıza doldurunuz. Lütfen tüm soruları yanıtlayınız.

ANKET FORMU NASIL DOLDURULUR

Yanıtlamak için her sorudan sonra yalnızca "D" ya da "Y" seçeneğini işaretlemeniz yeterlidir.

ÖRNEK;	DOĞRU	YANLIŞ
Bu anket formunu nasıl dolduracağımı biliyorum.	D	Y

(Bu anket formunu nasıl dolduracağınızı biliyorsanız, ifadenin DOĞRU olduğunu göstermek için "D"yi yuvarlak içine alınız.)

* *

Her ifadeyi dikkatlice okuyunuz, ancak yanıtlarken çok zaman harcamayınız.

Lütfen bütünüyle yanıttan emin olmasanız bile, her ifadeyi yanıtlayınız.

Doğru ya da yanlış yanıtlar olmadığını unutmayınız ve sadece kendi kişisel görüş ve duygularınızı belirtiniz.

Copyright © 1987, 1992 C. R. Cloninger
Türkçe TCI © 2001 Samet Köse, Kemal Sayar

Türkçe TCI'nin eser sahipliği hakları Samet Köse ve Kemal Sayar'a aittir. Yazarların izni olmaksızın her türlü kullanımı eser sahipliğinden doğan hakların ihlalini oluşturur ve etik değildir.

		DOĞRU	YANLIŞ
1.	Çoğu kimse zaman kaybı olduğunu düşünse bile, sırf eğlence ya da heyecan olsun diye çoğu kez yeni şeyler denerim.	D	Y
2.	Çoğu kimseyi kaygılandıran durumlarda bile, genellikle her şeyin iyiye gideceğine güvenirim.	D	Y
3.	Çoğu kez güzel bir konuşma ya da şiirden derinden etkilenirim.	D	Y
4.	Çoğu kez koşulların kurbanı olduğumu düşünürüm.	D	Y
5.	Benden çok farklı olsalar bile, genellikle başkalarını olduğu gibi kabul ederim.	D	Y
6.	Mucizelerin olabileceğine inanırım.	D	Y
7.	Beni inciten kimselerden intikam almak hoşuma gider.	D	Y
8.	Bir şeye yoğunlaştığımda, çoğu kez zamanın nasıl geçtiğinin farkına varmam.	D	Y
9.	Çoğu kez yaşamımın, pek az bir amacı ya da anlamı olduğunu düşünürüm.	D	Y
10.	Herkesin karşısına çıkabilecek sorunlara çözüm bulmakta, yardımcı olmak isterim.	D	Y
11.	Belki de yaptığımdan daha fazlasını başarabilirim, ancak bir şeye ulaşmak için kendimi gereğinden fazla zorlamada bir yarar görmüyorum.	D	Y
12.	Başkaları endişelenecek bir şey olmadığını düşünseler bile, tanıdık olmayan ortamlarda çoğu kez gergin ve endişeli hissederim.	D	Y
13.	İşleri yaparken geçmişte nasıl yapıldığını düşünmeksizin, çoğu kez o anda nasıl hissettiğimi temel alarak yaparım.	D	Y
14.	İşleri başkalarının arzularına bırakmaktansa, genellikle kendi tarzıma göre yaparım.	D	Y
15.	Çoğu kez çevremdeki kimselerle öylesine bağlantılı olduğumu düşünürüm ki, sanki aramızda bir ayrılık yokmuş gibi gelir.	D	Y
16.	Benden farklı düşünceleri olan kimselerden, genellikle hoşlanmam.	D	Y
17.	Çoğu durumda doğal tepkilerim, geliştirmiş olduğum iyi alışkanlıklara dayanır.	D	Y
18.	Bir çok eski arkadaşımın güvenini kaybedecek olsam bile, zengin ve ünlü olmak için, yasal olan hemen her şeyi yapardım.	D	Y
19.	Çoğu kimseden daha çok tedbirli ve denetimliyimdir.	D	Y
20.	Bir şeylerin yanlış gidebileceğinden endişelendiğim için, çoğu kez yapmakta olduğum işi bırakmak zorunda kalırım.	D	Y
21.	Yaşantı ve duygularımı kendime saklamak yerine, arkadaşlarımla açıkça tartışmaktan hoşlanırım.	D	Y
22.	Çoğu kimseden daha az enerjiye sahibim ve daha çabuk yorulurum.	D	Y
23.	Yapmakta olduğum işe kendimi fazla kaptırıp başka her şeyi unuttuğumdan, çoğu kez “dalgın” olarak adlandırılırım.	D	Y
24.	Ne yapmak istediğimi seçmede kendimi nadiren serbest hissederim.	D	Y
25.	Bir başkasının duygularını, çoğu kez kendiminkiler kadar dikkate alırım.	D	Y
26.	Bir kaç saat sessiz ve hareketsiz kalmaktansa, çoğu zaman biraz riskli şeyler (sarp tepeler ve keskin virajlar üzerinde araba kullanmak gibi) yapmayı tercih ederim.	D	Y
27.	Tanımadığım kimselere güven duymadığım için, çoğu kez yabancılarla tanışmaktan kaçınırım.	D	Y
28.	Başkalarını edebileceğim kadar çok memnun etmekten hoşlanırım.	D	Y
29.	Bir iş yaparken “yeni ve geliştirilmiş” yollardan çok, eski “denenmiş ve doğru” yolları tercih ederim.	D	Y

30.	Zaman yokluğu nedeniyle, genellikle işleri önemlilik sırasına göre yapamam.	D	Y
31.	Çoğu kez hayvanları ve bitkileri yok olmaktan korumaya yarayacak işler yaparım.	D	Y
32.	Çoğu kez başka herkesten daha zeki olmayı dilerim.	D	Y
33.	Düşmanlarımın acı çektiğini görmek bana hoşnutluk verir.	D	Y
34.	Her ne zaman olursa olsun, çok düzenli olmak ve kişiler için kurallar koymaktan hoşlanırım.	D	Y
35.	Dikkatim coğu kez başka bir şeye kaydığı için, uzunca bir süre aynı ilgilerimi sürdürmem güçtür.	D	Y
36.	Tekrarlanmış uygulamalar bana, çoğu anlık dürtüler ya da telkinlerden daha güçlü olan, iyi alışkanlıklar kazandırdı.	D	Y
37.	Genellikle o kadar kararlıyımdır ki, başkaları vazgeçtikten sonra bile çalışmaya devam ederim.	D	Y
38.	Yaşamda, bilimsel olarak açıklanamayan bir çok şeye hayran olurum.	D	Y
39.	Bırakabilmeyi dilediğim bir çok kötü alışkanlığım var.	D	Y
40.	Sorunlarıma çoğu kez bir başkasının çözüm sağlamasını beklerim.	D	Y
41.	Çoğu kez nakit param bitinceye ya da aşırı kredi kullanıp borçlanıncaya değin para harcarım.	D	Y
42.	Gelecekte bir hayli şanslı olacağımı düşünürüm.	D	Y
43.	Hafif rahatsızlık ya da gerginlikten çoğu kimseye göre daha geç kurtulurum.	D	Y
44.	Yalnız olmak beni her zaman rahatsız etmez.	D	Y
45.	Gevşemekteyken, çoğu kez beklenmedik içgörü ya da anlayış parıltıları yaşarım.	D	Y
46.	Başkalarının benden ya da işleri yapma tarzımdan hoşlanıp hoşlanmadıklarına pek aldırmam.	D	Y
47.	Herkesi memnun etmek mümkün olmadığı için, genellikle kendim için ne istiyorsam sadece onu edinmeye çalışırım.	D	Y
48.	Görüşlerimi kabul etmeyen kimselere karşı sabırlı değilimdir.	D	Y
49.	Çoğu kimseyi pek de iyi anladığım söylenemez.	D	Y
50.	Ticarette başarılı olmak için sahtekar olmak zorunda değilsiniz.	D	Y
51.	Bazen kendimi doğayla öylesine bağlantılı hissederim ki, her şey tek bir canlı organizmanın parçasıymış gibi görünür.	D	Y
52.	Konuşmalarda konuşmacı olmaktan çok, iyi bir dinleyiciyimdir.	D	Y
53.	Çoğu kimseden daha çabuk öfkelenirim.	D	Y
54.	Bir grup yabancıyla tanışmak zorunda olduğumda, çoğu kimseden daha sıkılganımdır.	D	Y
55.	Çoğu kimseden daha duygusalımdır.	D	Y
56.	Bazen neler olacağını sezmeme olanak veren, bir “altıncı his”e sahipmişim gibi gelir.	D	Y
57.	Birisi beni bir şekilde incitti mi, genellikle acısını çıkartmaya çalışırım.	D	Y
58.	Tutumlarımı, büyük ölçüde denetimim dışındaki etkiler belirler.	D	Y
59.	Her gün amaçlarıma doğru bir adım daha atmaya çalışırım.	D	Y
60.	Çoğu kez başka herkesten daha güçlü olmayı dilerim.	D	Y
61.	Karar vermeden önce işler konusunda uzunca süre düşünmeyi tercih ederim.	D	Y

62.	Çoğu kimseden daha çok çalışırım.	D	Y
63.	Çok kolay yorulduğumdan, çoğu kez kısa uykulara ya da ek dinlenme dönemlerine gereksinim duyarım.	D	Y
64.	Başkalarının hizmetinde olmaktan hoşlanırım.	D	Y
65.	Üstesinden gelmem gereken geçici sorunlara aldırmaksızın, daima her şeyin yolunda gideceğini düşünürüm.	D	Y
66.	Çok miktarda para biriktirmiş olsam bile, kendim için para harcamaktan hoşlanmakta güçlük çekerim.	D	Y
67.	Çoğu kimsenin bedensel olarak tehlike hissettiği durumlarda, genellikle sakin ve güvenli kalırım.	D	Y
68.	Sorunlarımı kendime saklamaktan hoşlanırım.	D	Y
69.	Kişisel sorunlarımı, çok az ya da kısa bir süre tanıdığım kişilerle tartışmakta sakınca görmem.	D	Y
70.	Seyahat etmek ya da yeni yerler araştırmaktansa, evde oturmaktan hoşlanırım.	D	Y
71.	Kendilerine yardımı dokunamayan zayıf kimselere yardım etmenin zekice olduğunu düşünmüyorum.	D	Y
72.	Bana haksız davranmış olsalar bile, başkalarına haksızca davranırsam, zihinsel huzurum kalmaz.	D	Y
73.	İnsanlar genellikle bana nasıl hissettiklerini anlatırlar.	D	Y
74.	Çoğu kez sonsuza değin genç kalabilmeyi dilerim.	D	Y
75.	Yakın bir arkadaşı kaybedince, genellikle çoğu kimseden daha çok üzüntü duyarım.	D	Y
76.	Bazen sanki zaman ve mekanda sonu ve sınırı olmayan bir nesnenin parçasıymışım hissine kapılırım.	D	Y
77.	Bazen başkalarına karşı sözcüklerle açıklayamadığım bir bağlantı hissederim.	D	Y
78.	Bana geçmişte haksızlık yapmış olsalar bile, başka kimselerin duygularını dikkate almaya çalışırım.	D	Y
79.	Katı kurallar ve düzenlemeler olmaksızın, insanların her istediklerini yapabilmeleri hoşuma gider.	D	Y
80.	Bir grup yabancıyla buluşunca, bana dostça davranmadıkları söylenmiş olsa bile, rahat ve açık sözlü olmayı sürdürürdüm.	D	Y
81.	Gelecekte bir şeylerin kötüye gideceği konusunda, genellikle çoğu kimseden daha endişeliyimdir.	D	Y
82.	Bir karara varmadan önce genellikle her olguyu etraflıca düşünürüm.	D	Y
83.	Başkalarına karşı sempatik ve anlayışlı olmanın, pratik ve katı düşünceli olmaktan daha önemli olduğunu düşünürüm.	D	Y
84.	Çoğu kez etrafımdaki tüm nesnelere karşı güçlü bir bütünlük duyusu hissederim.	D	Y
85.	Çoğu zaman "Süperman" gibi özel güçlerimin olmasını dilerim.	D	Y
86.	Başkaları beni çok fazla denetliyor.	D	Y
87.	Öğrendiklerimi başkalarıyla paylaşmaktan hoşlanırım.	D	Y
88.	Dinsel yaşantılar, yaşamımın gerçek amacını anlamamda bana yardımcı olmuştur.	D	Y
89.	Çoğu kez başkalarından çok şey öğrenirim.	D	Y
90.	Tekrarlanmış uygulamalar, başarılı olmamda bana yardımı olan, pek çok şeyde iyi olmama olanak vermiştir.	D	Y

91. Söylediğimin abartılı ve gerçek olmadığını bilmeme rağmen, genellikle başkalarının bana inanmalarını sağlayabilirim.	D	Y
92. Hafif rahatsızlık ya da gerginlikten kurtulmak için daha fazla dinlenme, destek ya da güvenceye gereksinim duyarım.	D	Y
93. Yaşamak için ilkeler olduğunu ve hiç kimsenin sonunda acı çekmeksizin bunları çiğneyemeyeceğini bilirim.	D	Y
94. Başka herkesten daha çok zengin olmak istemem.	D	Y
95. Dünyayı daha iyi bir yer haline getirmek için, kendi yaşamımı severek riske ederdim.	D	Y
96. Bir şey hakkında uzunca süre düşündükten sonra bile, mantıksal nedenlerimden çok duygularıma güvenmeyi öğrendim.	D	Y
97. Bazen yaşamımın, herhangi bir insandan daha büyük bir manevi güç tarafından yönetildiğini düşünürüm.	D	Y
98. Bana adice davranan birisine, genellikle adice davranmaktan hoşlanırım.	D	Y
99. Son derece pratik ve duygularına göre hareket etmeyen birisi olarak tanınırım.	D	Y
100. Benim için, birisiyle konuşurken düşüncelerimi düzenlemek kolaydır.	D	Y
101. Beklenmedik haberlere çoğu kez öylesine güçlü tepki veririm ki, pişmanlık duyduğum şeyler söyler ya da yaparım.	D	Y
102. Duygusal yakarışlardan (kötürüm kalmış çocuklara yardım istendiğindeki gibi) oldukça çok etkilenirim.	D	Y
103. Yapabileceğimin en iyisini yapmak istediğim için, genellikle kendimi çoğu kimseden daha çok zorlarım.	D	Y
104. Öyle çok kusurluyum ki, bu yüzden kendimi pek de sevmem.	D	Y
105. Sorunlarıma uzun vadeli çözümler aramak için çok az zamanım var.	D	Y
106. Sırf ne yapılacağını bilmediğim için, çoğu kez sorunların gereğine bakamam.	D	Y
107. Çoğu kez zamanın akışını durdurabilmeyi dilerim.	D	Y
108. Yalnızca ilk izlenimlerime dayanarak karar vermekten nefret ederim.	D	Y
109. Biriktirmektense, para harcamayı yeğlerim.	D	Y
110. Bir öyküyü daha gülünç hale getirmek ya da birisine şaka yapmak için, doğruları eğip bükmeyi genellikle iyi beceririm.	D	Y
111. Sorunlar olsa bile, bir arkadaşlığın hemen her zaman sürüp gitmesine çalışırım.	D	Y
112. Utandırılır ya da aşağılanırsam, çok çabuk atlatırım.	D	Y
113. Çok gergin, yorgun ya da endişeli olacağım için, işleri genelde yürütme tarzımdaki değişmelere uyum sağlamam son derece güçtür.	D	Y
114. İşleri eski yapış tarzımda değişiklik yapmayı istemeden önce, genellikle çok iyi gerçekçi nedenler talep ederim.	D	Y
115. İyi alışkanlıklar edinebilmem için, beni eğiten kişilerden çok fazla yardıma gereksinim duyarım.	D	Y
116. Duygu-dışı algılamanın (telepati ya da önceden bilme gibi), gerçekten de mümkün olduğuna inanırım.	D	Y
117. Candan ve yakın arkadaşlarımın çoğu zaman benimle birlikte olmasını isterim.	D	Y
118. Uzun zaman pek başarılı olmasam bile, çoğu kez aynı şeyi defalarca yeniden denemeyi sürdürürüm.	D	Y
119. Hemen herkes korku dolu olsa bile, ben hemen her zaman rahat ve tasasız kalırım.	D	Y

120. Hüzünlü şarkı ve filmleri epeyce sıkıcı bulurum.	D	Y
121. Koşullar çoğu kez beni irademe karşı bir şeyler yapmaya zorlar.	D	Y
122. Benim için, benden farklı olan insanlara katlanmak güçtür.	D	Y
123. Mucize denilen çoğu şeyin, sadece şans eseri olduğunu düşünürüm.	D	Y
124. Birisi beni incitirse, intikam almaktansa kibar davranmayı yeğlerim.	D	Y
125. Çoğu kez yaptığım işin o kadar etkisinde kalırım ki, zaman ve mekandan kopmuş gibi o an içinde kaybolurum.	D	Y
126. Yaşamımın gerçek bir amacı ve önemi olduğunu sanmıyorum.	D	Y
127. Başkalarıyla olabildiğince iş birliği yapmaya çalışırım.	D	Y
128. Başarılarımdan dolayı içim rahattır ve daha iyisini yapmak için pek istekli değilimdir.	D	Y
129. Başkaları pek tehlike olmadığını düşünse de, tanıdık olmayan ortamlarda çoğu kez gergin ve endişeli hissederim.	D	Y
130. Tüm ayrıntıları bütünüyle düşünmeksizin, çoğu kez iç güdülerimi, önsezi ve sezgilerimi izlerim.	D	Y
131. Başkaları, benden istediklerini yapmayacağım için, çoğu kez benim aşırı bağımsız olduğumu düşünürler.	D	Y
132. Çoğu kez etrafımdaki tüm kişilerle güçlü manevi ve duygusal bağlantım olduğunu hissederim.	D	Y
133. Benden farklı değer yargıları olan insanları sevmek, genellikle benim için kolaydır.	D	Y
134. Başkaları benden daha çoğunu beklese bile, olabildiğince az iş yapmaya çalışırım.	D	Y
135. İyi alışkanlıklar benim için "ikinci mizaç" olmuştur ve hemen her zaman otomatik ve kendiliğinden davranışlardır.	D	Y
136. Başkalarının bir şey hakkında benden daha çok bilmesi gerçeğinden, çoğu zaman rahatsızlık duymam.	D	Y
137. Genellikle kendimi başkalarının yerinde hayal etmeye çalışır, böylece onları gerçekten anlayabilirim.	D	Y
138. Tarafsızlık ve dürüstlük gibi ilkeler yaşamımın bazı yönlerinde pek az rol oynarlar.	D	Y
139. Para biriktirmede çoğu kimseden daha iyiyimdir.	D	Y
140. Kendimi nadiren öfkelenmiş ve engellenmiş hisseder, işler yolunda gitmediğinde hemen başka etkinliklere dalarım.	D	Y
141. Çoğu kimse önemli olmadığını düşünse bile, çoğu kez işlerin değişmez ve düzenli biçimde yapılmasında ısrar ederim.	D	Y
142. Hemen her sosyal durumda, kendimi oldukça güvenli ve emin hissederim.	D	Y
143. Özel düşüncelerimden nadiren sözettiğimden, arkadaşlarım duygularımı anlamakta güçlük çekerler.	D	Y
144. Çoğu kimse bana yeni ve daha iyi bir yol olduğunu söylese bile, işleri yapış tarzımı değiştirmekten nefret ederim.	D	Y
145. Bilimsel olarak açıklanamayan şeylere inanmanın, akıllıca olmadığını düşünürüm.	D	Y
146. Düşmanlarımın acı çektiğini hayal etmekten hoşlanırım.	D	Y
147. Çoğu kimseden daha fazla enerjim var ve daha geç yorulurum.	D	Y
148. Yaptığım her işte ayrıntılara dikkat etmekten hoşlanırım.	D	Y
149. Endişeye kapıldığımdan, arkadaşlarım her şeyin yolunda gideceğini söyleseler bile, yapmakta olduğum işi bırakırım.	D	Y

150. Çoğu kez başka herkesten daha güçlü olmayı dilerim.	D	Y
151. Genellikle ne yapacağımı seçmede serbestimdir.	D	Y
152. Çoğu kez kendimi yapmakta olduğum işe o kadar kaptırırım ki, bir an nerede olduğumu unuturum.	D	Y
153. Bir ekibin üyeleri, paylarını nadiren dürüstçe alırlar.	D	Y
154. Bir kaç saat sessiz ve hareketsiz kalmaktansa, çoğu zaman riskli şeyler (planörle uçmak ya da paraşütle atlamak gibi) yapmayı tercih ederim.	D	Y
155. Çoğu zaman dürtüsel olarak o kadar çok para harcarım ki, tatil yapmak gibi özel planlar için bile para biriktirmek bana güç gelir.	D	Y
156. Başkalarını memnun etmek için kendi bildiğimden şaşmam.	D	Y
157. Yabancılarla birlikteyken hiç sıkılgan değilimdir.	D	Y
158. Çoğu kez arkadaşlarımın arzularına boyun eğerim.	D	Y
159. Zamanımın çoğunu, gerekli gibi görünen, ancak benim için gerçekte önemsiz olan şeylere harcarım.	D	Y
160. Ticari kararlarda, neyin doğru neyin yanlış olduğuna ilişkin dinsel ya da ahlaki ilkelerin çok etkili olması gerektiğini düşünmem.	D	Y
161. Çoğu kez başkalarının yaşantılarını daha iyi anlamak için, kendi yargılarımı bir kenara koymaya çalışırım.	D	Y
162. Alışkanlıklarımın çoğu, bana değerli amaçlara ulaşmada zorluk çıkarır.	D	Y
163. Dünyayı daha iyi bir yer yapmak için; savaş, yoksulluk ya da haksızlıkları önlemeye çalışmak gibi gerçekten de kişisel fedakarlıklar yaptım.	D	Y
164. Gelecekte olabilecek kötü şeyler hakkında hiç endişelenmem.	D	Y
165. Kendi denetimimi yitirecek kadar, hemen hiç heyecanlanmam.	D	Y
166. Düşündüğümden daha uzun sürerse, çoğu kez o işi bırakırım.	D	Y
167. Başkalarının benimle konuşmalarını beklemektense, konuşmaları kendim başlatmayı tercih ederim.	D	Y
168. Bana yanlış yapan birisini, çoğu zaman, çabucak bağışlarım.	D	Y
169. Davranışlarımı, büyük ölçüde denetimim dışındaki etkiler belirler.	D	Y
170. Yanlış bir önsezi ya da hatalı bir ilk izlenim nedeniyle, çoğu kez kararlarımı değiştirmek zorunda kalırım.	D	Y
171. İşlerin yapılması için, bir başkasının ön ayak olmasını beklemeyi tercih ederim.	D	Y
172. Genellikle başkalarının görüşlerine saygı duyarım.	D	Y
173. Yaşamdaki rolümün berraklaşmasına yol açan, kendimi çok coşkulu ve mutlu hissettiğim yaşantılarım olmuştur.	D	Y
174. Kendim için bir şeyler satın almak eğlendiricidir.	D	Y
175. Kendimin duygu dışı algılar yaşantıladığıma inanırım.	D	Y
176. Beynimin düzgün çalışmadığına inanırım.	D	Y
177. Davranışlarıma yaşamım için koyduğum belli amaçlar, güçlü şekilde yol gösterir.	D	Y
178. Genellikle başkalarının başarısına ön ayak olmak aptalcadır.	D	Y
179. Çoğu kez sonsuza değin yaşamak isterim.	D	Y
180. Genellikle soğukkanlı ve başkalarından ayrı kalmaktan hoşlanırım.	D	Y

181. Hüzünlü bir film seyrederken, ağlama ihtimalim çoğu kimseden daha fazladır.	D	Y
182. Hafif rahatsızlık ya da gerginlikten çoğu kimseye göre daha çabuk kurtulurum.	D	Y
183. Cezalandırılmayacağımı sandığımda, çoğu kez kuralları ve düzenlemeleri ihlal ederim.	D	Y
184. Çoğu ayartıcı durumda, kendime güvenebilmem için iyi alışkanlıklar geliştirmemi sağlayacak, çok sayıda uygulama yapmam gerekir.	D	Y
185. Keşke başkaları bu kadar çok konuşmasalar.	D	Y
186. Önemsiz ya da kötüymüş gibi görünseler bile, herkes itibar ve saygı ile muamele görmelidir.	D	Y
187. Yapılması gerekli olan işleri sürdürebilmek için, çabuk kararlar vermekten hoşlanırım.	D	Y
188. Yapmaya çalıştığım her şeyde, genellikle şansım açıktır.	D	Y
189. Çoğu kimsenin tehlikeli nitelendirebileceği şeyleri (ıslak ya da buzlu bir yolda hızlı araba kullanmak gibi), genellikle kolayca yapabileceğime dair kendime güvenirim.	D	Y
190. Başarılı olma şansı olmadıkça, bir şey üzerinde çalışmayı sürdürmede bir yarar görmüyorum.	D	Y
191. İşleri yaparken yeni yollar araştırmaktan hoşlanırım.	D	Y
192. Eğlence ya da heyecan için para harcamaktansa, biriktirmekten zevk alırım.	D	Y
193. Bireysel haklar, herhangi bir grubun gereksinimlerinden daha önemlidir.	D	Y
194. Kendimi, ilahi ve olağanüstü bir manevi güçle temas içinde hissettiğim yaşantılarım oldu.	D	Y
195. İçinde, ansızın varolan herşeyle berrak ve derinden bir aynılık duygusu edindiğim, epeyce coşkulu anlarım oldu.	D	Y
196. İyi alışkanlıklar, işleri istediğim şekilde yapmamı kolaylaştırır.	D	Y
197. Çoğu kimse benden daha çok çare bulucu görünür.	D	Y
198. Sorunlarım için, çoğu kez başkalarını ve koşulları sorumlu tutarım.	D	Y
199. Bana kötü davranmış olsalar bile, başkalarına yardım etmekten hoşnutluk duyarım.	D	Y
200. Çoğu kez, tüm yaşamın kendisine bağlı olduğu manevi bir gücün parçasıymışım hissine kapılırım.	D	Y
201. Arkadaşlarla birlikteyken bile, çok fazla "açılmamayı" tercih ederim.	D	Y
202. Kendimi zorlamaksızın, genellikle tüm gün boyunca bir şeylerle "meşgul" kalabilirim.	D	Y
203. Başkaları çabuk bir karar vermemi isteseler bile, karar vermeden önce hemen her zaman tüm olgular hakkında ayrıntılı düşünürüm.	D	Y
204. Bir şeyi yanlış yaptığımı anladığımda, sıkıntıdan öyle kolayca kurtulamam.	D	Y
205. Çoğu kimseden daha mükemmeliyetçiyimdir.	D	Y
206. Bir şeyin doğru mu yanlış mı olduğu, sadece bir görüş meselesidir.	D	Y
207. Şimdiki doğal tepkilerimin, genellikle ilkelerim ve uzun vadeli amaçlarımla tutarlı olduğunu düşünürüm.	D	Y
208. Tüm yaşamın, bütünüyle açıklanamayacak bir manevi düzen ya da güce bağlı olduğuna inanırım.	D	Y
209. Bana kızgın oldukları söylenmiş olsa bile, yabancılarla tanıştığımda güvenli ve rahat olacağımı düşünürüm.	D	Y
210. İnsanlar yardım, sempati ve sıcak bir anlayış bulmak için bana yaklaşmanın kolay olduğunu söylerler.	D	Y

211. Yeni düşünceler ve etkinliklerden heyecan duymada, çoğu kimseye gore daha geriyimdir.	D	Y
212. Bir başkasının duygularını incitmemek için söylenecek olsa bile, yalan söylemekte sıkıntı yaşarım.	D	Y
213. Hoşlanmadığım bazı insanlar var.	D	Y
214. Başka herkesten daha fazla hayran olunmak istemem.	D	Y
215. Sıradan bir şeye bakarken, çoğu kez olağan üstü bir şey olur ve sanki onu ilk kez görüyormuşum duygusuna kapılırım.	D	Y
216. Tanıdığım çoğu kimse, başka kimin incineceğine aldırmaksızın, yalnızca kendisini düşünür.	D	Y
217. Yeni ve alışılmadık bir şey yapmak zorunda olduğumda, genellikle gergin ve endişeli hissederim.	D	Y
218. Çoğu kez kendimi tükenmişlik sınırına dek zorlar ya da gerçekte yapabileceğimden daha fazlasını yapmaya çalışırım.	D	Y
219. Kimileri para konusunda aşırı cimri ya da eli sıkı olduğumu düşünür.	D	Y
220. Mistik yaşantı söylentileri, muhtemelen sadece birer hüsnü kuruntudan ibarettir.	D	Y
221. Sonucunda acı çekeceğimi bilsem bile, irade gücüm çok güçlü ayartmaları üstesinden gelmeyecek kadar zayıftır.	D	Y
222. Herhangi birisinin acı çektiğini görmekten nefret ederim.	D	Y
223. Yaşamımda ne yapmak istediğimi biliyorum.	D	Y
224. Yaptığım işin doğru mu yanlış mı olduğunu düşünüp taşınmak için, düzenli olarak zaman ayırırım.	D	Y
225. Çok dikkatli olmazsam, benim için işler çoğu kez ters gider.	D	Y
226. Kendimi keyifsiz hissettiğimde, yalnız kalmak yerine arkadaşlarla birlikte olduğumda, genellikle daha iyi hissederim.	D	Y
227. Aynı şeyi yaşantılamayan birisiyle duyguları paylaşmanın, mümkün olmadığını düşünürüm.	D	Y
228. Çevremde olup bitenlerden bütünüyle haberdar olmadığım için, başkalarına çoğu kez sanki başka bir dünyadaymışım gibi gelir.	D	Y
229. Keşke başka herkesten daha iyi görünüşlü olsam.	D	Y
230. Bu anket formunda çok yalan söyledim.	D	Y
231. Arkadaş canlısı oldukları söylenmiş olsa bile, genellikle yabancılarla tanışmak zorunda kalacağım sosyal ortamlardan uzak dururum.	D	Y
232. Baharda çiçeklerin açmasını, eski bir arkadaşı yeniden görmek kadar severim.	D	Y
233. Zor durumları, genellikle bir meydan okuma ya da fırsat olarak değerlendiririm.	D	Y
234. Benimle ilgili kimseler, işleri benim tarzıma göre yapmayı öğrenmek zorundadırlar.	D	Y
235. Sahtekar olmak, yalnızca yakalandığınızda sorun yaratır.	D	Y
236. Hafif rahatsızlık ve gerginlikten sonra bile, genellikle çoğu kimseden daha fazla güvenli ve enerjik hissederim.	D	Y
237. Herhangi bir kağıdı imzalamam istendiğinde, herşeyi okumaktan hoşlanırım.	D	Y
238. Yeni bir şeyler olmadığında, genellikle heyecan ya da coşku verici bir şey aramaya başlarım.	D	Y
239. Bazen keyifsiz olurum.	D	Y
240. Ara sıra insanların arkasından konuşurum.	D	Y

EK-2
TCI Derecelendirme Anahtarı

Tüm ölçekler için pozitif olarak puanlandırılan (D= 1, Y= 0) maddelerin altı çizilmemiş, negatif olarak puanlandırılan maddelerin altı çizilmiştir (D= 0, Y= 1). Aşağıdaki maddeler ise TCI'ın bir bölümü olarak puanlanmamıştır: 69, 75, 101, 111, 118, 134, 140, 170, 176, 190, 213, 230, 239, 240.

Yenilik Arayışı (**Novelty Seeking**)

NS1 Keşfetmekten heyecan duyma-Kayıtsız bir katılık (11 madde)
1, 29, 52, 70, 99, 114, 144, 167, 191, 211, 238

NS2 Dürtüsellik-İyice düşünme (10 madde)
13, 35, 61, 82, 108, 130, 148, 187, 203, 237

NS3 Savurganlık-Tutumluluk (9 madde)
19, 41, 66, 109, 139, 155, 174, 192, 219

NS4 Düzensizlik-Düzenlilik (10 madde)
34, 53, 79, 91, 110, 141, 165, 183, 204, 212

NS Toplam Yenilik Arayışı Puanı (40 madde): NS1 + NS2 + NS3 + NS4

Zarardan Kaçınma (**Harm Avoidance**)

HA1 Beklenti endişesi ve karamsarlık-Sınırsız iyimserlik (11 madde)
2, 20, 42, 65, 81, 112, 119, 149, 164, 188, 225

HA2 Belirsizlik korkusu (7 madde)
12, 26, 67, 129, 154, 189, 217

HA3 Yabancılardan çekinme (8 madde)
27, 54, 80, 100, 142, 157, 209, 231

HA4 Çabuk yorulma ve dermansızlık (9 madde)
22, 43, 63, 92, 113, 147, 182, 202, 236

HA Toplam Zarardan Kaçınma Puanı (35 madde): HA1 + HA2 + HA3 + HA4

Ödül Bağımlılığı (**Reward Dependence**)

RD1 Duygusallık (10 madde)
3, 28, 55, 83, 102, 120, 158, 181, 210, 224

RD3 Bağlanma (8 madde)
21, 44, 68, 117, 143, 180, 201, 226

RD4 Bağımlılık (6 madde)
14, 46, 71, 131, 156, 193

RD Toplam Ödül Bağımlılığı Puanı (24 madde): RD1 + RD3 + RD4

Sebat Etme (**Persistence**)

P Sebat etme (8 madde)
11, 37, 62, 103, 128, 166, 205, 218

Kendi Kendini Yönetme (**Self-Directedness**)

S1 Sorumluluk alma-Kınama (8 madde)
4, 24, 58, 86, 121, 151, 169, 198

S2 Amaçlılık-Amaçsızlık (8 madde)
9, 30, 59, 105, 126, 159, 177, 223

S3 Beceriklilik (5 madde)
40, 106, 171, 197, 233

S4 Kendini kabullenme- Kendisiyle çekişme (11 madde)
32, 60, 74, 85, 94, 107, 136, 150, 179, 214, 229

S5 Aydınlanmış ikinci mizaç (12 madde)
17, 36, 39, 90, 104, 115, 135, 162, 184, 196, 207, 221

S Toplam Kendi Kendini İdare Etme Puanı (44 madde): S1 + S2 + S3 + S4 + S5

İş Birliği Yapma (**Cooperativeness**)

C1 Sosyal kabullenme-Sosyal hoşgörüsüzlük (8 madde)
5, 16, 48, 89, 122, 133, 172, 234

C2 Empati duyma-Sosyal ilgisizlik (7 madde)
25, 49, 73, 137, 161, 185, 227

C3 Yardımseverlik-yardım sevmemezlik (8 madde)
10, 47, 64, 87, 127, 153, 178, 216

C4 Acıma-intikamcılık (10 madde)
7, 33, 57, 78, 98, 124, 146, 168, 199, 222

C5 Temiz kalplilik vicdanlılık-Kendi kendine yara sağlama (9 madde)
18, 50, 72, 93, 138, 160, 186, 206, 235

C Toplam İş Birliği Puanı (42 madde): C1 + C2 + C3 + C4 + C5

Kendi Kendini Aşma (**Self-Transcendence**)

ST1 Kendini kaybetme-Kendilik bilincinde yaşantı (11 madde)
8, 23, 45, 76, 96, 125, 152, 173, 195, 215, 228

ST2 Kişiler arası özdeşim-Kendi kendine ayrışma (9 madde)
15, 31, 51, 84, 95, 132, 163, 200, 232

ST2 Manevi kabullenme-Akılcı maddecilik (13 madde)
6, 38, 56, 77, 88, 97, 116, 123, 145, 175, 194, 208, 220

ST Toplam Kendi Kendini Aşma Puanı (33 madde): ST1 + ST2 + ST3

Copyright © 1992 C. R. Cloninger
Türkçe TCI © 2001 Samet Köse, Kemal Sayar
